# Electron hopping in conjugated molecular wires with application to solar cells

**DOI:** 10.1038/s41557-025-02034-0

**Published:** 2026-02-09

**Authors:** Fang Fang, Ang Li, Blaise L. Geoghegan, Thomas Webb, Yunfei Dang, Amanz Azaden, Troy L. Bennett, Kunrou Fu, Francesco Vanin, Adam J. Sills, Nicholas J. Long, Saif A. Haque, Maxie M. Roessler

**Affiliations:** 1 Department of Chemistry, Molecular Sciences Research Hub, Imperial College London, London, UK; 2 Centre for Processable Electronics, Molecular Sciences Research Hub, Imperial College London, London, UK

**Keywords:** Electronic materials, Surface assembly, Nanoscale devices

## Abstract

Electron transfer through molecular wires underpins numerous research fields, ranging from single-molecule electronics to fundamental biological processes and their application in (bio)electrocatalysis. Here we report a series of 1–3-nm-long ferrocene-terminated conjugated molecular wires, anchored to indium tin oxide electrodes, that exhibit an electron transfer mechanism dominated by hopping (with a *β* value of 0.043 Å^−1^). We show that the nature of the electrode, namely the small energy gap between the electron donor and acceptor, explains the unexpected electron transfer mechanism in these short wires. We demonstrate the applicability of these anchored molecular wires as the hole-extraction layer in a tin perovskite solar cell. We show improved performance in devices employing these molecular wires compared with the more conventional hole-extraction layers typically used in tin perovskite solar cells. This work not only opens avenues for mechanistic investigations of interfacial electron transfer using molecular wires but also showcases their potential impact in applications such as photovoltaics.

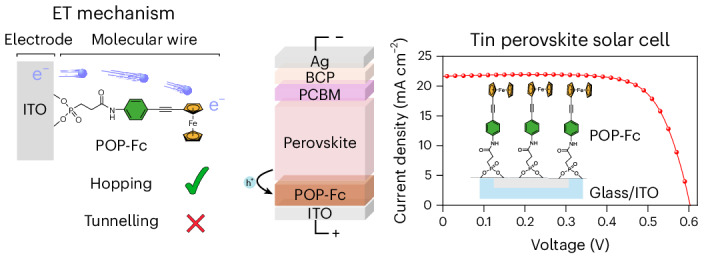

## Abstract (web summary)

Electron transfer in molecular wires is typically dominated by tunnelling at short lengths. Now it is shown that conjugated molecular wires anchored to indium tin oxide electrodes exhibit a hopping mechanism even at 1-nm lengths, enabling charge extraction in tin perovskite solar cells and improved device performance.

## Main

Understanding and achieving control over electron transfer (ET) between molecules and electrode surfaces is fundamental for developing materials with applications spanning organic electronics^1^, electrocatalysis^2,3^, solar photoconversion^4^ and biotechnology^5^. Molecular wires promise to advance this much-needed understanding and control by forming chemical circuitry between analyte and electrode to facilitate efficient ET over nanometre-scale distances^6,7^. The customizable molecular structure of molecular wires allows for the precise tuning of electronic and mechanical characteristics, resulting in targeted properties for their desired application^8^.

Interfacing molecular wires with materials, typically through the formation of self-assembled monolayers (SAMs)^9^, enables their integration into complex nanostructures, facilitating the development of nanoelectronics devices^10^. Their compatibility with biological systems makes them promising candidates for applications in biosensing^11^, as well as for mechanistic investigations of complex ET enzymes^12^. SAMs have been recognized as an effective tool for interfacial modification in solution-processed thin-film electronics^13,14^ and form a part of key strategies in the development of charge transport layers in perovskite solar cells (PSCs)^15^, which have been successfully adopted in efficient and stable positive-intrinsic-negative (p-i-n) PSCs with various perovskite bandgaps^16–18^. Additional benefits of the ultrathin nature of SAMs over other conventional materials include high optical transparency, mechanical flexibility and long-term stability^19^. Indium tin oxide (ITO) has emerged as a particularly versatile material in devices, with thin ITO films being found in, for example, solar cells^20^ and touch screens^21^. In contrast to thin films, three-dimensional (3D) porous ‘hierarchical’ structures derived from ITO nanoparticles have opened up numerous applications within electrocatalysis and bioelectrocatalysis^22,23^ due to their increased and tuneable surface area. Understanding ET between molecules and the electrode surface is fundamentally important and can indeed limit performance^3^. Several experimental methods for measuring molecular conductivity, including scanning tunnelling microscopy^24,25^ and conductive atomic force microscopy (AFM)^26,27^, have revealed how conjugated molecular wires interact with metal electrode surfaces. However, the ET mechanism on ITO remains unknown and the methods currently employed for ‘flat’ Au or Hg surfaces are incompatible with ‘rugged’ ITO surfaces.

Electrochemical techniques are well suited for investigating long-range ET between surfaces and redox-active molecules^28^. Amongst the diverse electrochemical methodologies available, cyclic voltammetry (CV) is particularly suitable to study the heterogeneous ET rates of interfaced redox molecules^29^. Molecular-wire SAMs ‘bridging’ redox-active molecules and the electrode surface have been recognized as an excellent tool to study ET at material surface junctions^14,30,31^. The ET facilitated by the monolayer in these structures, occurring between the electrode and the redox moiety, is equivalent to the donor–bridge–acceptor model, a concept studied in homogeneous and biological systems^7^. Moreover, the kinetics of charge transfer have been investigated for redox-active ferrocene moieties attached to electrodes via both saturated and conjugated molecular wires^28,32–34^. However, the ET process between hierarchical ITO electrodes and redox-active molecules, such as ferrocene, remains unexplored and yet timely, considering the multitude of promising applications with ITO-based molecular wires.

In this Article, we showcase how molecular wires can serve as a tool to understand ET at ITO molecular junctions. Using electrochemistry, we examined the ET kinetics of ferrocene attached to ITO electrodes via highly conjugated oligo(*p*-phenylene-ethynylene) (OPE)^35–37^ molecular wire bridges of variable length. We found that ET is dominated by a hopping rather than a tunnelling mechanism, even for the shortest molecular wire. We rationalize this surprising result, guided by the extensive work carried out on Au-derived surfaces^10,27,28,38^. Inspired by the numerous studies focused on developing and using molecular wires in single-molecule electronics^8,39^, and motivated by their unknown benefit to molecular electronic devices, we investigated how our shortest wire, with the most favourable ET characteristics, performs in an ITO-derived tin PSC. We observed improved solar energy to electrical power conversion efficiency in devices employing this molecular wire as the hole-extraction layer compared with materials conventionally used in tin PSCs. These results, in combination with the enhanced mechanistic understanding (on both hierarchical and flat surfaces), pave the way towards using redox-active molecular wires not only for improved performance of solar cells but also for applications in (bio)electrocatalysis and beyond.

## Results and discussion

### Design and synthesis of molecular wires

**Fig. 1:**
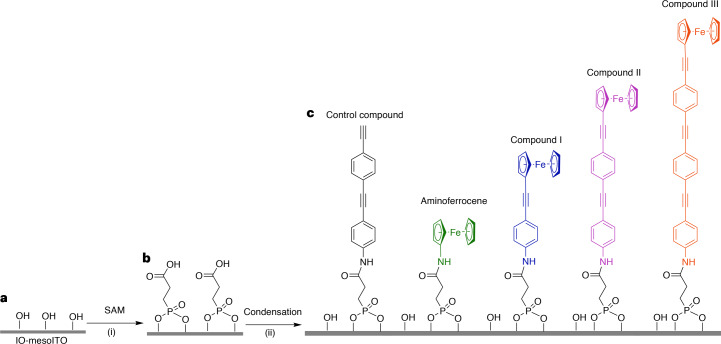
Ferrocene-appended molecular wires and their application to surface functionalization. **a**–**c**, Schematic of the preparation of the ferrocene-appended OPE-based molecular wires and control compounds: pre-treated IO-mesoITO surface (**a**), SAM-modified IO-mesoITO surface (**b**), achieved under reaction conditions (i), and ferrocene-appended molecular wires covalently anchored to SAM-modified IO-mesoITO surfaces (**c**), achieved under reaction conditions (ii). Reaction conditions: (i) IO-mesoITO, 3-phosphonopropionic acid (1 mM), ethanol (10 ml), 24 h; (ii) water (10 ml, 1 h), *N*-(3-dimethylaminopropyl)-*N*′-ethylcarbodiimide hydrochloride (EDC; 1 equiv.), *N*-hydroxysuccinimide (NHS; 1 equiv.), R-NH_2_ (for compounds I–III and the control compound) in *N*,*N*-dimethylformamide (DMF; 1 equiv. 10 ml, 24 h). See Methods for further synthetic details. Source data

The molecular wires (Fig. 1) were designed to be highly modular with regards to the facile addition of *p*-phenylene-ethynylene units to tune their length, anchorage onto ITO surfaces and addition of a redox-active group to interrogate their ET properties. Ferrocene was chosen as the redox-active group as it exhibits high thermal and chemical stability, and with its reversible, one-electron oxidation to ferrocenium, it serves as a gold standard for electrochemical studies^40^. A primary amine group was used at the opposite terminus of our molecular wires^27,28^ as this facilitates covalent attachment to pre-modified ITO surfaces through amide formation^41^.

Although the synthesis of OPEs is well documented^27^, simultaneously achieving the modular addition of several aryl units with ultimate control over the total molecular length, the position of each unit and type of functionalization has not yet been demonstrated. Here we show a modified synthetic strategy to achieve these goals, developing the use of orthogonal protecting groups at each stage of the assembly of the molecular wire architecture (Methods and Supplementary Section 2). The ferrocene-appended OPE-based molecular wires, compounds I–III (Fig. 1 and Supplementary Section 2), and aminoferrocene were characterized by NMR spectroscopy, mass spectrometry and ultraviolet–visible spectrophotometry (Supplementary Sections 2–4).

### Electrode functionalization and characterization

Although thin-film ITO is a highly versatile conductive material with extensive applications in solar cells^42^, hierarchical variants such as ‘inverse-opal mesoporous ITO’ (IO-mesoITO) have opened the door to a wider range of (spectro)electrochemical investigations, including in the fields of catalysis, biocatalysis and solar-driven reactions^3,43,44^. Their large effective surface area results in a highly enhanced loading of redox species compared with conventional flat-surface electrodes^23,45^. Compounds I–III were therefore anchored onto porous hierarchical ITO electrodes (Fig. 1) with a large surface area to maximize electrochemical sensitivity and hence facilitate interrogation of the ET properties of our molecular wires^22^. IO-mesoITO electrodes with 700 nm diameter pores and a 0.25 cm^2^ calculated geometric surface area were assembled using a bottom-up approach starting from ITO nanoparticles (Methods). The molecular wires were covalently attached through condensation of the RNH_2_ group onto the 3-phosphonopropanoic acid-based SAM-modified IO-mesoITO surface (Fig. 1 and Methods).

**Fig. 2:**
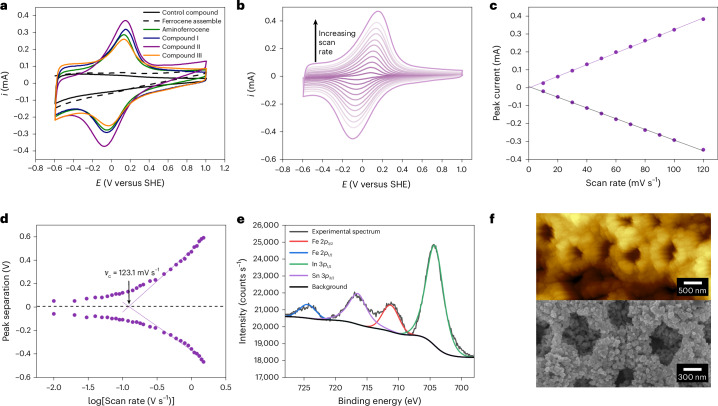
Characterization of surface-anchored ferrocene-derived molecular wires. **a**, Cyclic voltammograms of aminoferrocene and compounds I–III (Fig. 1) anchored to IO-mesoITO electrode surfaces in phosphate-buffered saline (PBS; pH 7), recorded at a scan rate of 100 mV s^−1^ at room temperature. Controls with the two-unit OPE wire anchored to the ITO surface (control compound 4-[(4-ethynylphenyl)ethynyl]aniline, Fig. 1) and an IO-mesoITO electrode incubated in a DMF solution of ferrocene for 24 h at room temperature and then subsequently rinsed with 5 ml DMF three times (non-anchored ferrocene) are also shown. **b**, Cyclic voltammograms of compound II recorded at various scan rates (10–100 mV s^−1^). **c**, Randles–Sevcik plot of compound II on IO-mesoITO. **d**, Trumpet plot of compound II derived from scan rates of 10–1,500 mV s^−1^. The critical scan rate (*ν*_c_) was derived from a linear fit of the anodic and cathodic peak data points at high scan rates. See Supplementary Section 4 for the corresponding data shown in **b**–**d** for compounds I and III. **e**, High-resolution XPS spectrum of the Fe 2*p* region of compound II covalently anchored to a SAM-modified IO-mesoITO electrode. **f**, AFM (top) and SEM (bottom) images of an IO-mesoITO electrode surface. Source data

The SAM-modified IO-mesoITO electrodes were investigated by film electrochemistry, scanning electron microscopy (SEM) and X-ray photoelectron spectroscopy (XPS). All SAM-modified IO-mesoITO electrodes exhibited similar behaviour in CV with regards to peak potentials, peak currents and peak separations. The covalent anchoring of compounds I–III and aminoferrocene onto the SAM-modified IO-mesoITO electrodes resulted in reversible redox couples (Fig. 2a), with anodic and cathodic peak current ratios close to unity (Extended Data Table 1), confirming reversible ET. The reduction potentials were similar for all of the wires (Extended Data Table 1), and no redox peaks were observed with either the control compound (a two-unit OPE wire without the ferrocene appendage) or non-covalently anchored ferrocene (Fig. 2a). The linear relationship between peak current and scan rate, as illustrated for compound II in Fig. 2b,c (see Supplementary Section 4 for aminoferrocene and compounds I and III), demonstrates successful surface functionalization of all of the molecular wires^46^. XPS revealed the presence of the ferrocene Fe on the ITO structures, which was not observed for the control electrodes (Fig. 2e and Supplementary Fig. 29). The surface coverage of compounds I–III was quantified by integrating the CV peaks to calculate the number of electroactive molecules, in conjunction with the IO-mesoITO surface area derived from Brunauer–Emmett–Teller (BET) analysis (Fig. 2f and Methods), and was similar for all compounds (Extended Data Table 1). The distance of the ferrocene unit to the electrode surface did not exceed 3 nm (Extended Data Table 1) and therefore the molecular wire SAM tilt angle, which only becomes pronounced above 6 nm (ref. ^47^), is negligible.

Having demonstrated effective ET between ITO and ferrocene via the molecular wires, we proceeded to investigate the kinetic properties of this ET. Laviron analysis (Methods) and the resulting ‘trumpet plot’ (Fig. 2d and Supplementary Sections 4 and 5) enabled the determination of the apparent rate constants (*k*_app_), critical scan rates (*ν*_*c*_) and ET coefficients (*α*), as summarized in Extended Data Table 1. The low *α* values suggest film structures with exposed ITO sites^48^. The critical scan rates compare favourably with other redox systems immobilized on hierarchical ITO^3^, but are not high compared with systems immobilized onto other electrode materials such as gold^28^. Importantly, the derived rate constants provide information on the ET mechanism within the molecular wires, as discussed in the subsequent section.

### ET mechanism

It is generally accepted that charge transport (CT) between an electrode and a molecular wire can transition from a tunnelling (Sommerfeld regime) to a ‘hopping’ (Arrhenius regime) process by increasing the length of the molecular wire (Supporting Section 6.2)^27,38,49^. However, recent studies on metallic electrodes suggest that at elevated temperatures both tunnelling and hopping processes display Arrhenius-like behaviour (non-zero activation energies) and temperature dependence^50,51^. Our system distinguishes itself from such systems in the following aspects: (1) ITO is the electrode material rather than Au and (2) the anchoring group is an -OH from 3-phosphonopropionic acid rather than a Au–S bond. Understanding the effect of these differences on the CT mechanism (ET in our case) requires a closer look at the thermodynamic and kinetic parameters listed in Extended Data Table 1. To establish which mechanism prevails in our system, we investigated both the thermal dependence of the ET and its rate of decay with increasing molecular wire length.

Based on the predictions of Marcus theory (Supplementary Section 6.2), numerous studies in the field of single-molecule electronics have focused on optimizing the distance between the redox-active site and the electrode. The measure of the dependence of ET rate constant on distance is given by *β*, calculated according to equation (1)^52^:

1 $${k}_{\mathrm{app}}={k}_{0}\exp (-\beta L),$$

where *k*_0_ is a kinetic pre-exponential factor and *L* is the CT distance. This distance is taken to be equal to the molecular length. We assumed that the distance between the electrode surface and the ferrocene units at the distal termini of the molecular wires (*L*_max_ = 3 nm) is equal to *L* due to the SAM tilt angle being negligible when the molecular length is <6 nm (ref. ^10^). By definition (equation (1)), the *β* value applies only to exponentially decaying processes, yet it is often employed to describe all types of CT. For small values of *β*, parallels to band transport or a so-called π-way for CT via coherent tunnelling, also termed hopping, have been drawn^47^.

**Fig. 3:**
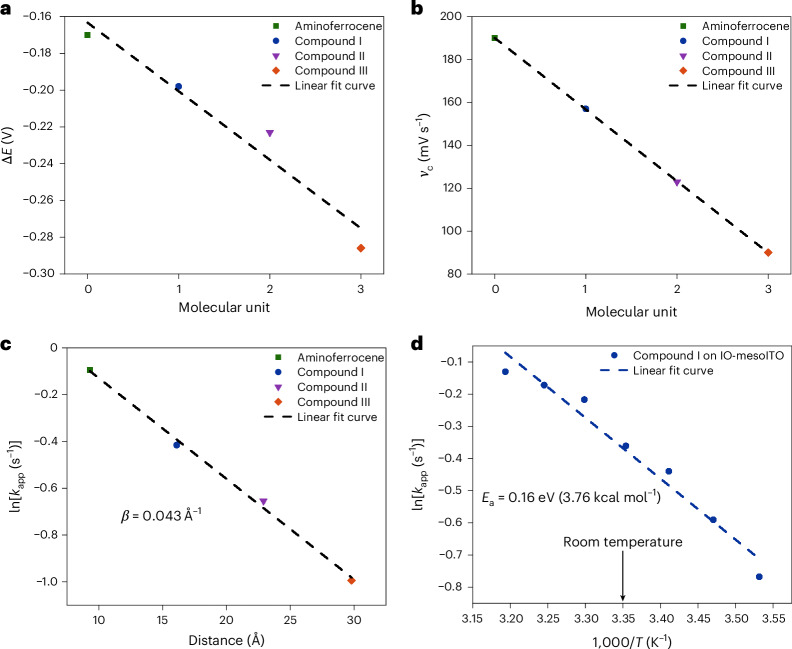
Investigation of the ET mechanism on hierarchical ITO electrodes. **a**, Peak separation in cyclic voltammograms (recorded at a scan rate of 100 mV s^−1^) as a function of the molecular wire length. ‘Molecular unit’ refers to the number of phenylene-ethynylene units present in the molecular wire. The colours match the colours of the structures in Fig. 1a. **b**, Critical scan rate as a function of molecular wire length. **c**, Natural logarithmic plot of equation (1), giving the *β* value for the investigated molecular wires. **d**, Arrhenius plot for Compound I. Source data

**Fig. 4:**
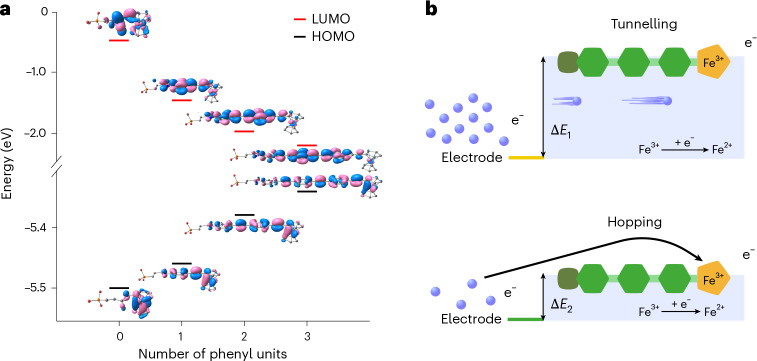
Proposed mechanism of ET in our molecular wires anchored onto hierarchical ITO. **a**, HOMO–LUMO energy gaps for neutral (Fe^II^) phosphonopropionamido-appended aminoferrocene and compounds I–III, calculated by density functional theory. **b**, Schematic illustration of tunnelling-dominated ET^27,68^ in short molecular wires and our proposed hopping-dominated ET mechanism^56^. Source data

Figure 3c shows the linear relationship between increasing molecular length and the natural logarithm of the apparent rate constant *k*_app_. Fitting to equation (1) provides a value for *β* of 0.043 Å^−1^. This value of *β* is low in the general context of molecular wires and is representative of a small attenuation of the ET rate constant upon increasing the molecular length of the wire and is around five times smaller than reported for short (<3 nm) OPE wires attached to gold surfaces^27,28^. Instead, our *β* value of 0.043 Å^−1^ is similar to that reported for long OPE wires^26,27,53^, in which theoretical and experimental evidence suggests that the ET mechanism switches from tunnelling to hopping^27^ (Supplementary Section 6). Thus, our *β* value suggests a hopping mechanism between the ITO electrode and ferrocene unit rather than tunnelling, which would be expected to exhibit an exponential decay of *k*_app_ with increasing molecular length and a large associated *β* value^6,53^. Inspection of the molecular orbitals (MOs) of compounds I–III shows that as the OPE linker is extended up to three units, the highest occupied MO (HOMO) of the neutral Fe^II^ species is delocalized away from the ferrocene unit and across the OPE linker, reducing the distance to the ITO electrode surface (Fig. 4a). This close proximity of the molecular wire’s HOMO to the ITO surface may facilitate hopping of the electron between the electrode and redox centre (reducing Fe^III^ to Fe^II^), even as the molecular length is increased, providing an explanation for the low *β* value that we measured. Although there have been many studies tackling the tunnelling versus hopping question on Au electrode surfaces^27,28^, the mechanism of CT in molecular wires anchored to ITO, a semiconductor, has not yet been investigated.

An Arrhenius-like temperature dependence of the ET rate constant has traditionally been considered indicative of hopping mechanisms and has been used to distinguish between tunnelling and hopping regimes. Yet, recent works on conductance across Au junctions linked by molecular wires have demonstrated that thermal broadening of the Fermi level may lead to thermally activated tunnelling at elevated temperatures, a process that also exhibits Arrhenius-like behaviour, much like hopping^51^. Hence, a temperature dependence of the ET rate constant alone does not prove a hopping mechanism over tunnelling, even though a thermal dependence is not always observed for tunnelling events^54^. However, in conjunction with our exceptionally low *β* value, a temperature dependence would indicate hopping at room temperature and above^26^, either as a sole process or as a major contributor to a multiprocess regime^50^. We thus investigated the temperature dependence of *k*_app_ for compound I (4-(3-phosphonopropionamido)phenylethynylferrocene), the shortest molecular wire, which would exemplify the most ‘extreme’ case of hopping.

Figure 3d shows that *k*_app_ for compound I on IO-mesoITO is dependent on temperature in the range of 283 K to 313 K. Using the Arrhenius equation (Supplementary Equation (4)), the activation energy (*E*_a_) was calculated to be 0.16 eV, which is small and of the same order of magnitude as values reported for long conjugated molecular wires on Au (ref. ^26^). Such low *E*_a_ values have been reasoned to be one of the main factors governing the transition from a tunnelling to a hopping regime, enabling charge to rapidly spread out over the entire bridge and inducing wire-like behaviour^47^. The combination of an exceptionally low *β* value and thermally dependent ET rate constant suggests that a hopping mechanism plays a substantial, if not a dominant, role in the overall ET kinetics in our ITO–molecular wire systems. To explain this unexpected result, we next turned to investigate the factors that determine *β* values.

The *β* value depends on the nature of the bonding in the molecular backbone and is sensitive to the electronic coupling between the molecular wire and the electrode surface^8,10^, in this case the IO-mesoITO electrode. The significance of aligning the energy levels of the highest-occupied electronic state (Fermi level, *E*_f_) of the donor (in this case the electrode) with the lowest-unoccupied MO (LUMO) of the acceptor (that is, the LUMO of the molecular wire in the oxidized Fe^III^ state) for efficient charge/electron injection into the wire has long been recognized^55^. Indeed, the rate of charge conduction through the wire increases rapidly as the overlap between these two levels increases. To better understand the energy alignment of the molecular wire with the ITO electrode compared with Au, we measured the *E*_f_ values of ITO, Au and compound I. Kelvin probe measurement (Supplementary Table 4) showed that our IO-mesoITO electrode has a more positive *E*_f_ (−4.60 eV) than Au (−4.90 eV) and is closer in energy to that of the ITO-anchored molecular wires (−4.43 eV to −4.31 eV), thus leading to better energy alignment in our system compared with what is typically encountered in single-molecule electronics^27^. In the context of ET kinetics, the energy difference between the Fermi levels (Δ*E*_f_) of isolated IO-meso-ITO and compound I anchored to IO-meso-ITO is 0.17 eV, an almost perfect match with *E*_a_, determined in the temperature-dependent ET study. This energy alignment makes a hopping mechanism plausible even for short molecular wires, where one would typically expect tunnelling to dominate^38^. The proclivity of our ITO-anchored molecular wires for the hopping ET mechanism may also be related to (1) the linking of the OPE terminus to ITO through two P–O bonds rather than one bond per molecular wire and (2) the use of amine-terminated OPE wires, which have shown shallower conductance decays than thiol analogues^27,28^.

Although we cannot say unambiguously that thermally activated tunnelling is absent in the IO-mesoITO-based molecular wire systems presented here, our combined experimental investigations (low *β* value, Arrhenius behaviour, Δ*E*_f_ **≈** *E*_a_) support that hopping dominates in the ET process. Given that ET in short (<2 nm) molecular wires usually occurs through tunnelling, we next sought to explore how compound I performs in a tin PSC application as part of the hole transport layer.

### Application of compound I as hole-extraction layer in tin PSCs

**Extended Data Fig. 1:**
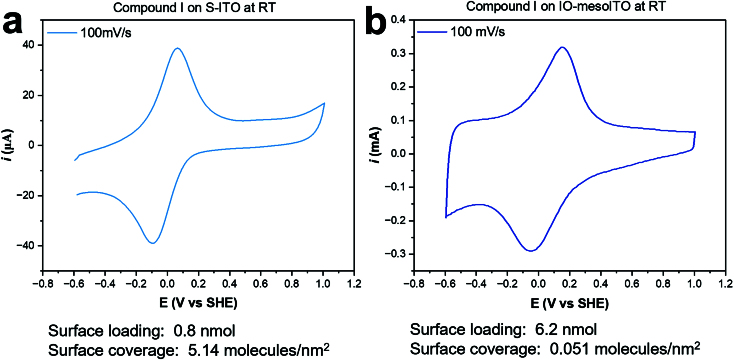
Comparation of Compound I on flat vs hierarchical indium tin oxide (ITO) electrodes. **a** cyclic voltammogram of Compound I on sputtered ITO (S-ITO) and **b** inverse-opal mesoporous ITO (IO-mesoITO) obtained at a scan rate 100mVs^−1^ at room temperature, in phosphate buffer (pH 7.0). Note that for S-ITO the surface coverage is a gross approximation only (see supplementary information Section 7.3 for information on how surface coverages were estimated). Source data

**Extended Data Fig. 2:**
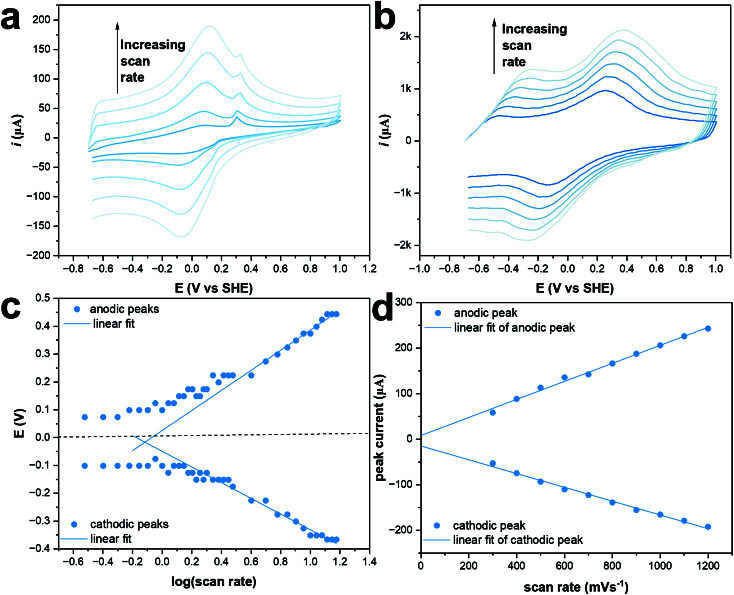
Electrochemical dataset of Compound I on sputtered indium tin oxide electrodes (S-ITO) at 20 °C. Cyclic voltammograms of Compound I on S-ITO in phosphate buffer (pH 7.0) at scan rates of **a** 200-600 mVs^−1^ (the feature at 0.3 V is an artefact that vanished at higher scan rates) and **b** 3-8 Vs^−1^ with the resulting **c** Trumpet plot and **d** Randles-Sevcik plot. Source data

**Extended Data Fig. 3:**
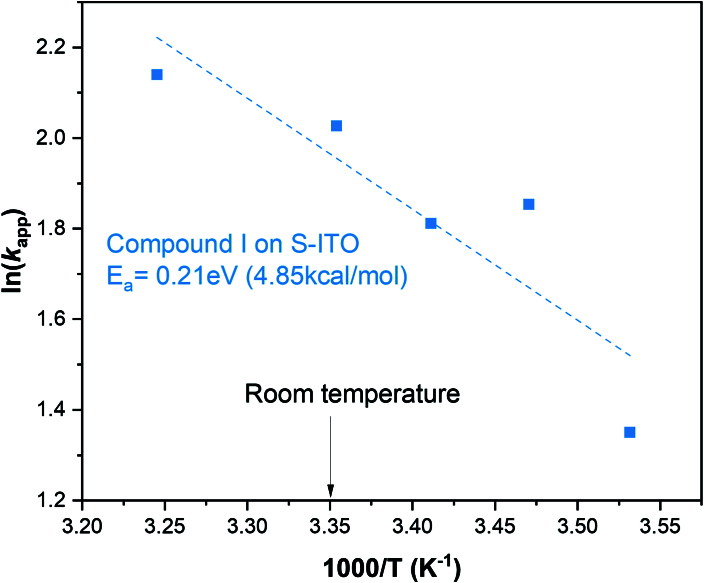
Arrhenius plot of Compound I on sputtered indium tin oxide electrodes (S-ITO). See Fig. 3 in the main paper for the corresponding plot on hierarchical ITO electrodes. Source data

As flat, transparent, conducting oxide substrates are typically used for the fabrication of solar cells, we prepared electrodes analogous to the 3D IO-mesoITO systems by sputtering our electrode base with ITO (S-ITO). Recognizing the different structural characteristics of IO-mesoITO used in the above-mentioned mechanistic study and S-ITO used in PSCs^56^, we investigated the ET mechanism between S-ITO and compound I before incorporating them into the solar cell system. Compound I-functionalized S-ITO exhibited a tenfold enhancement in ET rate constant compared with the analogous IO-mesoITO electrode (Extended Data Figs. 1 and 2 and Supplementary Figs. 21–25), as expected from the more uniform, crystalline structure of two-dimensional S-ITO compared with 3D IO-mesoITO^57^. Although IO-mesoITO and S-ITO exhibit different kinetics (Extended Data Tables 1 and 2), the temperature dependence of the ET constant rate does not differ significantly (Extended Data Fig. 3) and both exhibit comparable values of *E*_a_ (0.16 eV versus 0.21 eV). Hence, we conclude that our S-ITO-based molecular wires exhibit a similar ET mechanism to that of the IO-mesoITO systems.

To demonstrate the applicability of OPE molecular wires as the hole-extraction layer in PSCs^58^, we functionalized commercial S-ITO electrodes with compound I using the two-step condensation methodology detailed earlier (Fig. 1), upon which we could fabricate tin PSCs. Successful deposition of the molecular wire was evidenced by the change in hydrophobicity, determined by contact angle measurements of the compound I-functionalized substrates (Supplementary Fig. 32). Aside from a minor increase in absorbance between 375 and 475 nm, attributed to the ferrocene moiety of the molecular wire, negligible changes were observed in the absorbance and transmittance spectra of the substrates following deposition of compound I (Supplementary Fig. 33). We next deposited tin perovskite films (Methods) with composition PEA_0.2_FA_0.8_SnI_3_ (where PEA and FA represent phenethylammonium and formamidinium cations, respectively)^59,60^. The absorbance and emission spectra of the PEA_0.2_FA_0.8_SnI_3_ perovskite film following deposition on S-ITO/compound I substrates are provided in Supplementary Fig. 34 and display no unexpected features compared with S-ITO.

**Fig. 5:**
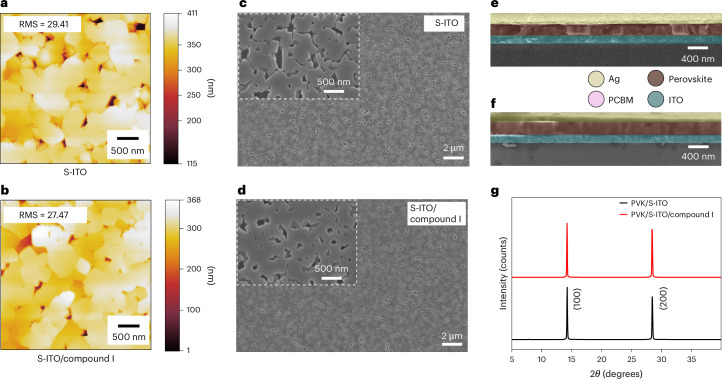
Structural characterization of tin perovskite films on ITO functionalized with compound I. **a**,**b**, AFM images demonstrating the perovskite film coverage and roughness (scale bar denotes maximum (white) and minimum (black) height) on S-ITO (**a**) and S-ITO/Compound I (**b**) substrates. **c**,**d**, SEM images showing the top view of the perovskite morphology on S-ITO (**c**) and S-ITO/compound I (**d**) substrates. Insets: magnifications of the SEM images. **e**,**f**, Cross-sectional SEM images of perovskite solar cells incorporating bare S-ITO (**e**) and S-ITO/Compound I (**f**) substrates. **g**, X-ray diffraction patterns of perovskite (PVK) films deposited on S-ITO and S-ITO/Compound I substrates. Source data

We used AFM to probe the uniformity and surface roughness of the deposited perovskite films and observed similar perovskite root mean square roughness values on S-ITO and S-ITO/compound I substrates (Fig. 5a,b and Supplementary Fig. 35). In addition, SEM showed no obvious differences in the perovskite films formed on S-ITO and S-ITO/compound I substrates (Fig. 5c,d). Similarly, cross-sectional images of the perovskites with and without compound I functionalization displayed little or no variation in film thickness (Fig. 5e,f and Supplementary Fig. 36). The crystallographic properties of the perovskite films were assessed using X-ray diffraction techniques (Fig. 5g). Peaks at approximately 14.2° and 28.5° characteristic of the (100) and (200) planes of perovskite films were observed, confirming the formation of high-quality perovskite layers on substrates with and without compound I (ref. ^61^).

**Fig. 6:**
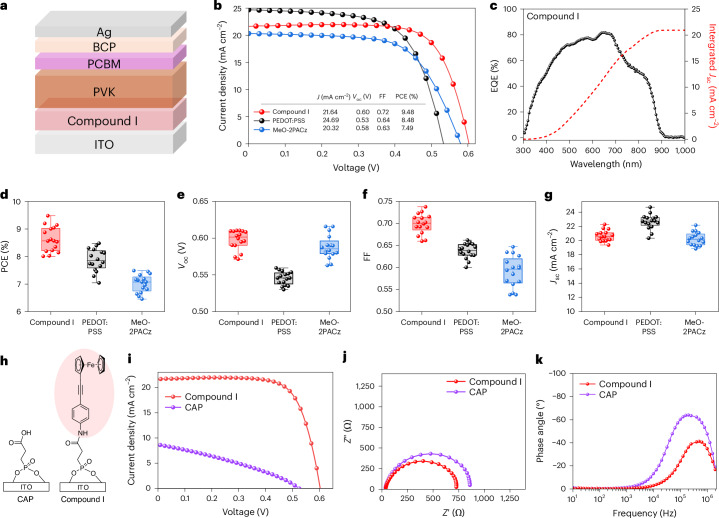
Characterization of compound I-derived tin PSCs. **a**, The architecture of a p-i-n tin PSC based on Compound I anchored on S-ITO. **b**, Comparison of the current density–voltage (*J*–*V*) curves of Compound I-based tin PSCs and other conventional PSC architectures. **c**, EQE and integrated *J*_sc_ of the best-performing compound I PSC configuration. **d**–**g**, Device performance statistics for 16 devices based on Compound 1, PEDOT:PSS and MeO-2PACz hole transport layers: PCE (**d**), *V*_oc_ (**e**), FF (**f**) and *J*_sc_ (**g**). The whiskers represent the minima and maxima, the bounds of the boxes represent the 25th and 75th percentiles, and the lines within the boxes are the statistical means. **h**, Chemical structures of the CAP molecule and Compound I. **i**, Comparison of the *J*–*V* characteristics of CAP and Compound I. **j**, EIS spectra of CAP and Compound I measured under illumination at 0.4 V. **k**, Bode plots of PSCs prepared with CAP and Compound I at 0.4 V (operational voltage). Source data

We fabricated solar cell devices based on the p-i-n architecture with compound I acting as the hole transport layer and a fullerene derivative [6,6]-phenyl-C_61_-butyric acid methyl ester (PCBM) serving as the electron transport layer (Fig. 6a). Current density–voltage (*J*–*V*) curves were collected to test the performance of compound I within a solar cell, showing a champion power conversion efficiency (PCE) of 9.48%. The external quantum efficiency (EQE) spectrum of the compound I-functionalized device was also measured (Fig. 6c). From this, an integrated current density of 20.94 mA cm^−2^ was calculated, closely matching the *J*–*V* data (21.64 mA cm^−2^). Stabilized device power output measurements showed no discernible drop-off in performance over a period of 300 s (Supplementary Fig. 37), and hysteresis data showed a similar performance in both the forward and reverse scan directions (Supplementary Fig. 38).

Additional devices were prepared with poly(3,4-ethylenedioxythiophene):poly(styrene sulfonate) (PEDOT:PSS), an established polythiophene-derived hole conductor^58^. Compared with these devices, the champion device prepared with compound I showed improved open circuit voltage (*V*_OC_) and fill factor (FF) values compared with the PEDOT:PSS devices, which was also reflected in the statistical analysis. Similarly, comparison of the photovoltaic characteristics of compound I with those of (2-(3,6-dimethoxy-9H-carbazol-9-yl)ethyl)phosphonic acid (MeO-2PACz), a widely used SAM for PSCs^62^, revealed improvements in the short-circuit current density *(J*_SC_) and FF for compound I. Devices prepared using Compound I experienced lower FF losses and a higher current than the MeO-2PACz PSCs, perhaps due to resistive tunnelling losses in the alkyl-carbazole-based SAMs^63^. This was further evidenced by the steeper *J*–*V* response around the *V*_oc_, indicating a lower series resistance in the device with compound I.

Finally, we considered the importance of the ferrocene terminal group in compound I by comparison with a simple carboxylated alkylphosphate (CAP) molecule (Fig. 6h). We observed a substantial deficit in performance in the device prepared without the ferrocene terminal group (Fig. 6i), which achieved a maximum PCE of only 2.26%. Device statistics with and without the ferrocene terminal group are shown in Supplementary Fig. 39 and highlight losses in both *J*_SC_ and FF for the device prepared with CAP. These losses, combined with a markedly reduced gradient in the *J*–*V* curve around the open-circuit voltage, indicate losses associated with high series resistance within the device, suggesting improved CT in the ferrocene-terminated substrates. The reduced resistive losses upon ferrocene inclusion are reflected in the electrochemical impedance spectroscopy (EIS) data (Fig. 6j,k). Samples prepared with ferrocene showed reduced CT resistance (*R*_CT_), from 836 Ω to 694 Ω, comparable to the value of 680 Ω obtained for PEDOT:PSS-based PSCs (Supplementary Fig. 40). Finally, Bode plots provide a useful method to understand the timescale at which photogenerated charges accumulate in the device. Comparing the two wires, we observed charge accumulation on shorter timescales in samples prepared with compound I, which we attribute to faster and more efficient hole transfer by virtue of the ferrocene terminal group. These findings are consistent with observations within the wider literature and highlight the importance of ferrocene termination in encouraging interfacial charge transfer^64–67^.

## Conclusion

We have investigated a series of conjugated molecular wires on hierarchical and flat ITO electrodes, identifying their ET mechanism and demonstrating their application in PSCs. We summarize our findings as follows:

1. Film electrochemistry enabled facile investigation of the ET mechanism on both hierarchical and flat ITO surfaces, complementing established mechanical methods available for ‘flat’ surfaces. This approach should be widely applicable, even to systems with complex surface morphologies, and takes full advantage of the tunability of the electrode surface and pore size.
2. We found that a hopping ET mechanism is possible even in a very short (1 nm) molecular wire, which until now was thought to occur predominantly via tunnelling. Even though we cannot rule out a contribution from thermally assisted tunnelling, we have demonstrated that reducing the energy gap between electrode and redox centre in weakly coupled molecular wires can induce a hopping mechanism at such short distances.
3. We applied the principles of single-molecule electronics to solar cells and showcased the use of our molecular wire in a tin PSC, which achieved a PCE of 9.48%. This proof of principle reveals a class of compounds that could have potential in a range of optoelectronic devices, including solar cells.

In the future, we envisage that our ITO-based molecular wire system and analytical tools could be applied to gain insights into (bio)electrocatalytic reactions by lodging large biomolecules within electrode pores. We also anticipate that further combinations of the fields of single-molecule electronics and solar cells could benefit from exploiting the toolbox of chemical synthesis to design target-specific molecular wires.

## Methods

### Materials

All synthetic procedures were conducted under an inert atmosphere (nitrogen) using standard Schlenk techniques, unless otherwise stated. All chemicals were of analytical grade, purchased from Sigma-Aldrich and used without further purification. Anhydrous solvents were obtained from a Grubbs-type solvent purification system (SPS) system and stored over 3 Å activated molecular sieves under an inert atmosphere. Glassware was dried for 12 h at 120 °C before use. Deuterated solvents for NMR spectroscopy were used as received. ITO nanoparticles for making hierarchical electrodes were purchased from Sigma-Aldrich (nanopowder with a particle size of <50 nm). Deionized ultrapure water (18 MΩ cm) was used in all experiments. PBS buffer was adjusted to pH 7.0 with concentrated aqueous solution of HCl.

All of the reagents used for PSC device preparation were purchased from the following suppliers: tin metal (Sn(0) beads, 99.98%), iodine (I_2_, flakes, 99%), tin(II) fluoride (SnF_2_, 99%) and toluene as anti-solvent (Sigma-Aldrich); FA iodide (FAI) and PEA iodide (PEAI; Greatcell Solar); PCBM (99.5%; Ossila); DMF and dimethylsulfoxide (DMSO; Acros Organics).

### Synthesis of compounds I–III

The molecular wires (Fig. 1) were synthesized as described in the Supplementary Information. Orthogonal protecting groups were used to extend the OPE wires in a controlled manner^27^. To protect the terminal alkyne, trimethylsilyl (TMS) and triisopropylsilyl (TIPS) protecting groups were chosen as they can be selectively removed under acidic and basic/nucleophilic conditions, respectively. By repetitions of the Sonogashira coupling and hydrolysis reactions, the OPE wires were extended in a controlled way, producing a series of TMS- and TIPS-protected OPE wires (Supplementary Section 1).

To generate the primary amine-terminated OPE wires from smaller subunits, we first used potassium carbonate, a weak base, to remove the TMS protecting group of the OPE subunit before coupling with 4-iodoaniline. However, due to low yields (≤5%), we used di-*tert*-butyl dicarbonate to protect the free amine of the terminal OPE subunit before coupling, which increased the yield to >60%. With the protected amine successfully incorporated onto one terminus of the OPE wire, tetrabutylammonium fluoride was used to remove the TIPS protecting group at the opposite terminus, followed by coupling with iodoferrocene to yield ferrocene- and *tert*-butyloxycarbonyl (BOC)-capped wires. As ferrocene is unstable in the strong acidic conditions required to remove the BOC protecting group in the final step, trifluoroacetamide was chosen instead as it can be removed under basic conditions (KOH) tolerated by ferrocene.

### Synthesis of SnI_2_

Tin metal (10 g, 84.7 mmol) was added to a nitrogen-flushed three-necked 250-ml round-bottomed flask equipped with a condenser. Degassed aqueous HCl (100 ml, 2 M) was subsequently added along with iodine (14 g, 55 mmol) and the stirred solution was heated to 180 °C. An excess of tin metal was required to help prevent the formation of SnI_4_ in solution. Upon reaching 150 °C, the solution quickly changed colour from dark brown to bright yellow and was allowed to reflux for 2 h. The solution was then allowed to cool to 70 °C before decanting it into a heated Schlenk flask via a cannula, separating the undissolved tin metal. The decanted solution was then allowed to cool slowly overnight, affording red needle-like SnI_2_ crystals. The supernatant was filtered and the crystals were washed with dilute degassed HCl (3 × 50 ml, 0.01 M) and subsequently dried under vacuum. Crude SnI_2_ yields of >80% with respect to I_2_ were achieved. The dried SnI_2_ crystals were stored in a glove box (<0.5 ppm H_2_O, <0.5 ppm O_2_) in opaque containers. Crude SnI_2_ was purified by vacuum sublimation (300 °C and 0.08 mbar) for ~4 h (or until no further deposition of yellow/orange SnI_4_ impurities was observed on the cold finger) before being dissolved in the perovskite precursor.

### Electrode preparation and functionalization

Hierarchical IO-mesoITO was assembled by a colloidal co-assembly approach using polystyrene beads (750 nm diameter) and ITO colloids (<50 nm diameter). After sintering, a mesoporous ITO skeleton was formed around the macroporous IO chambers. This approach produced interconnected macrochambers with a diameter of ~750 nm and connecting chambers with a width of 150 nm. Harnessing the strong affinity of ITO for phosphonate groups, 3-phosphonopropanoic acid was used to form a SAM after immersing the ITO electrode in concentrated phosphoric acid solution for 24 h. After the formation of the 3-phosphonopropanoic acid SAM on the pre-treated ITO, the NH_2_-terminated OPE wire was covalently attached to the surface of the electrode via a condensation reaction with the exposed RCOOH groups of the 3-phosphonopropionic acid units.

#### Preparation of IO-mesoITO electrodes

ITO nanoparticles (<50 nm diameter, 35 mg) were dispersed by sonication in MeOH–water (6:1 (v/v), 300 μl) for 3 h. A dispersion of polystyrene beads (750 nm diameter, 2.54% (w/v) suspension in water, 1 ml) was centrifuged (3 mins, 9,408 *g*, 4 °C), the supernatant removed and the polystyrene pellet was re-dispersed in MeOH (1 ml). The polystyrene bead dispersion was centrifuged again, the supernatant removed and the dispersion of ITO nanoparticles added to the polystyrene pellet. This mixture was thoroughly vortexed for 5 min and sonicated in ice-cold water (<5 °C) for 20 min to give a polystyrene–ITO dispersion in water.

Fluorine-doped tin oxide (FTO)-coated glass (7 Ω sq^−1^, thickness 2.2 mm, Sigma-Aldrich) was sonicated in isopropyl alcohol (15 min), then ethanol (15 min) and dried at 150 °C for 2 h. A parafilm template (circular, 5.7 mm diameter) was placed onto the dried FTO glass to define the geometrical surface area for the IO-mesoITO films, and the polystyrene–ITO dispersion was drop-cast onto this predefined area. Dropping 8 μl of the polystyrene–ITO dispersion over a geometrical surface area of 0.25 cm^2^ corresponds to a IO-mesoITO film thickness of 10 μm. If a thicker film was desired, the polystyrene–ITO mixture was deposited several times, with a drying period of at least a 4 h between deposits. The electrodes were heated at a rate of 1 °C min^−1^ from room temperature to 500 °C and annealed at this temperature for 20 min. The IO-mesoITO electrodes were then cleaned in 30% H_2_O_2_–H_2_O–30% NH_4_OH (1:5:1, v/v) at 70 °C for 15 min, rinsed with water and heated for 1 h at 180 °C to give a contaminant-free hydrophilic ITO surface.

### Electrode functionalization

#### Functionalization of IO-mesoITO electrodes

The IO-mesoITO electrodes were cleaned by rinsing with 2-propanol, followed by acetone and then water. They were then placed in 30% H_2_O_2_–H_2_O–30% NH_4_OH (1:5:1, v/v) at 70 °C for 1 h and afterwards washed with H_2_O. A 3-phosphonopropionic acid SAM was subsequently formed on the IO-mesoITO surface by incubating the electrodes in an ethanolic solution of 3-phosphonopropionic acid (1 mM) and NEt_3_ (2 mM) at room temperature for 24 h. The SAM-modified ITO (IO-mesoITO|SAM) electrodes were rinsed with EtOH, heated at 140 °C under N_2_ for 24 h, rinsed with EtOH and dried under vacuum at room temperature. The resulting clean SAM-modified electrodes were kept under vacuum before use in the next step. IO-mesoITO electrodes bearing COOH-terminated SAMs were immersed in a solution of EDC (10 mM) and NHS (10 mM) in deionized (DI) water for 1 h, washed with DI water, and immersed in a 2 mM solution of the desired molecular wire (compounds I–III) and aminoferrocene in DMF for 24 h.

#### Functionalization of S-ITO electrodes

S-ITO electrodes were purchased from Liaoning Youxuan New Energy Technology Co. Ltd. The S-ITO electrodes were cleaned by rinsing with 2-propanol, followed by water and then acetone. The cleaned S-ITO substrate was subsequently subjected to oxygen plasma treatment for 10 min. SAMs were subsequently formed on the S-ITO surface by incubating the electrodes for 24 h at room temperature in an ethanolic solution of 1 mM 3-phosphonopropionic acid and 2 mM NEt_3_. The SAM-modified ITO electrodes (S-ITO|SAM) were rinsed with ethanol, annealed at 140 °C under nitrogen for 24 h, rinsed again with ethanol and finally dried under vacuum at room temperature. The COOH-terminated SAM-modified S-ITO electrodes were activated in an aqueous solution of 10 mM EDC and 10 mM NHS for 1 h, rinsed with DI water and functionalized with 2 mM compound I in DMF for 24 h.

### Electrochemistry

#### CV measurements

All CV curves were recorded on an Ivium potentiostat. The electrochemical experiments were performed using a standard three-electrode configuration, with the functionalized IO-mesoITO electrode as the working electrode, Ag/AgCl (3 M KCl) as the reference electrode (World Precision Instruments) and a Pt wire as the counter electrode. PBS buffer (pH 7.0) was used as the electrolyte. Measurements were performed in a glove box (O_2_ < 1.5 ppm) using a standard glass cell (Southampton University Scientific Glassblowing Service). All potentials were converted to the standard hydrogen electrode scale. The shift in potential due to the *iR* drop (ohmic drop) was negligible and corrected after data collection (Supplementary Information). All measurements were carried out at room temperature unless otherwise stated.

#### CV analysis by the Laviron method

Thermodynamic and kinetic data were extracted from the CV data using the Laviron model based on equations (2) and (3):

2 $${\Delta E}_{{\rm{p}},{\rm{a}}}=\frac{-2.3{RT}}{\left(1-\alpha \right){nF}}\log \left(\frac{\left(1-\alpha \right){nF}}{{{RTk}}_{\mathrm{app}}}\right)-\frac{2.3{RT}}{\left(1-\alpha \right){nF}}\log \left(v\right)$$

3 $${\Delta E}_{{\rm{p}},{\rm{c}}}=\frac{-2.3{RT}}{\alpha {nF}}\log \left(\frac{\alpha {nF}}{{{RTk}}_{\mathrm{app}}}\right)-\frac{2.3{RT}}{\alpha nF}\log \left(v\right)$$

where Δ*E*_p,a_ and Δ*E*_p,c_ are the differences between the potential of the anodic (a) and cathodic (c) peaks and the formal reduction potential obtained by averaging the anodic and cathodic potentials recorded in the range of 10 –100 mV s^−1^, *n* is the number of electrons transferred, *α* is the electron-transfer coefficient, *ν* is the scan rate and *k*_app_ is the apparent rate constant for ET. *R*, *T* and *F* are the ideal gas constant, absolute temperature and Faraday constant, respectively. The calculations are detailed in Supplementary Section 6.

For the surface system, the cyclic voltammetry peak current is linearly proportional to the scan rate and is given by (4)^69^:

4 $${i}_{{\rm{p}}}={\left(\frac{{n}^{2}{F}^{2}}{4RT}\right)}ACv$$

where *i*_p_ is the peak current, *n* is the number of electrons transferred in the redox cycle, *F* is the Faraday constant, *R* is the universal gas constant, *T* is the absolute temperature, *A* is the electrode surface area, *C* is the surface concentration of redox-active species and *ν* is the scan rate (in V s^−1^).

The surface coverage (*Γ*, in molecules nm^−2^) of the molecular wires was estimated from the oxidation peaks in the voltammograms of aminoferrocene and compounds I–III (Extended Data Table 1) using equation (5):

5 $$\varGamma =\frac{Q}{nFA}$$

where *Q* is the electric charge obtained by integrating the area under the oxidation peak, *n* is the number of electrons involved in the ET process, *F* is the Faraday constant and *A* is the surface area of the electrode. As IO-mesoITO was used to increase the surface area, the actual surface area was determined by BET analysis, which is widely used for accurately measuring the surface area of metal–organic frameworks^2^. The average surface area was calculated to be *S* = 57.701 m^2^ g^−1^, based on two BET easurements (Supplementary Fig. 30). The charge injected was determined by integrating the first oxidation peak in the cyclic voltammogram for each scan rate. The surface loading of S-ITO was determined on the basis of its geometric area as the exact mass of the commercially available S-ITO loaded on the substrate could not be determined (Extended Data Fig. 1, Extended Data Table 1 and Supplementary Section 7.3).

The separation of anodic and cathodic peaks with increasing scan rate (Fig. 2d) can be used to calculate the apparent rate constant of the system according to the Laviron formalism using the following equations:

6 $${E}_{{\rm{p}},{\rm{c}}}=E^\circ {\prime} -\frac{2.3{RT}}{\alpha {nF}}\log \left[\frac{\alpha {nFv}}{{{RTk}}_{\mathrm{app}}}\right]$$

7 $${E}_{{\rm{p}},{\rm{a}}}=E^\circ {\prime} -\frac{2.3{RT}}{(1-\alpha ){nF}}\log \left[\frac{(1-\alpha ){nFv}}{{{RTk}}_{\mathrm{app}}}\right]$$

where *E*_p,a_ is the potential of the anodic peak, *E*_p,c_ is the potential of the cathodic peak, *E*°′ is the formal potential calculated by averaging the anodic and cathodic potentials at low scan rates, *v* is the scan rate, *α* is the electron-transfer coefficient, *k*_app_ is the apparent rate constant, *R* is the ideal gas constant, *T* is the absolute temperature, *F* is the Faraday constant and *n* is the number of electrons transferred. *E*_p_ − *E*°′ was plotted against the log of the scan rate (trumpet plot) to determine the ET parameters.

### Perovskite device fabrication

#### Substrate preparation

S-ITO substrates (purchased from Psiotec, 1.2 cm × 1.2 cm, 15 Ω cm^−^^2^) were rinsed with acetone and then sequentially sonicated in soap, distilled water, acetone and isopropanol. The clean ITO substrate was treated with oxygen plasma for 10 min. Compound I-functionalized ITO substrates were prepared following the method outlined in ‘Electrode functionalization’. For the final functionalization step, the following conditions were used: 1 mM solution of compound I in DMF for 12 h at 60 °C. The coated substrates were washed with DMF and then heated at 100 °C for 30 min. To fabricate the PEDOT:PSS/ITO layers, 30 μl PEDOT:PSS was spin-coated onto clean S-ITO substrates at 6,000 r.p.m. for 30 s, followed by heating at 140 °C for 30 min. To fabricate the MeO-2PACz/ITO layers, 30 μl MeO-2PACz dissolved in ethanol (0.2 mg ml^−1^) was spin-coated onto clean S-ITO substrates at 2,000 r.p.m. for 30 s. All samples were stored in a N_2_-filled glove box before use.

#### Device fabrication

A 0.8 M (PEA)_0.2_(FA)_0.8_SnI_3_ perovskite precursor solution was prepared inside a glove box. Initially, PEAI, FAI, SnI_2_ and SnF_2_ powders were weighed in a molar ratio of 0.2:0.8:1:0.1. The mixed powders were then dissolved in DMF–DMSO (4:1, v/v) and filtered using a 0.2 µm polytetrafluoroethylene filter, yielding 500 µl of a 0.8 M PEA_0.2_FA_0.8_SnI_3_ perovskite precursor solution. This solution was heated with stirring at 50 °C overnight and subsequently spin-coated onto pre-cleaned ITO substrates at 4,000 r.p.m. for 20 s. At 7 s of spinning, 0.5 ml toluene was dripped onto the spinning substrate as an anti-solvent. After spin-coating, the substrate was annealed at 70 °C for 20 min. Next, PCBM in chlorobenzene (20 mg ml^−1^) was spin-coated onto the perovskite films at 2,000 r.p.m. for 30 s, followed by bathocuproine in IPA (0.5 mg ml^−1^) at 5,500 r.p.m. for 30 s. Finally, a 100 nm Ag electrode was thermally evaporated onto the device at a pressure of 10^−6^ mbar. The active area of the pixel was defined to be 0.045 cm^2^ using a mask.

#### Device characterization

UV–visible spectroscopy was conducted using a Shimadzu UV-2600 integrating-sphere spectrophotometer. X-ray diffraction analysis was performed using a Bruker D2-phaser diffractometer equipped with a Cu Kα (Ni-filtered) radiation source, operating at 40 kV and 40 mA. Photoluminescence spectra were measured on an FLS 1000 fluorescence spectrophotometer (Edinburgh Instruments).

The *J*–*V* properties were characterized under 1-Sun (AM1.5G) illumination using a Keithley 2400 source meter at a scan rate of 50 mV s^−1^ in forward and reverse bias with a xenon lamp (Newport). The light intensity was calibrated using an independently calibrated silicon photodiode. Devices were kept under inert conditions before measurement. Each pixel was isolated by scraping the surrounding perovskite before loading the device into a homemade chamber in a N_2_ glove box. Each device comprised six pixels, with each pixel having an effective area of 0.045 cm^2^.

EQEs were measured with a Bentham EQE measurement unit incorporating a halogen lamp chopped at 188 Hz via a Newport monochromator. Data collection was facilitated by a four-point probe connected to a lock-in amplifier. The monochromatic beam was calibrated using a calibrated silicon photodiode, and the EQE spectra were measured using Bentham EQE software. The device was loaded into a nitrogen glove box and sealed in the measuring chamber during the measurements.

EIS was performed using a Gamry 1010E digital potentiostat. Samples were measured under N_2_ in a sealed chamber with a multiplexer.

### NMR spectroscopy

NMR analysis was performed using a Bruker 400 MHz NMR spectrometer. [D_6_]DMSO and CDCl_3_ (Thermo Scientific, 0.75 ml ampoules) were used as solvent for the NMR samples. All samples were prepared by dissolving ~10 mg of solid compound into ~0.6 ml [D_6_]DMSO. Chemical shifts are reported in ppm relative to the residual signals of DMSO or chloroform as the internal standard (2.50 and 7.26 ppm, respectively).

### Electrospray ionization mass spectrometric analysis

Direct analysis was performed using a Waters LCT Premier time-of-flight (TOF) mass spectrometer. The mass spectrometer was operated in positive and negative electrospray ionization modes over an *m*/*z* scan range of 100 to 2,000 with the following parameters: source capillary of 2,500 V, sample cone of 35 V, desolvation temperature of 350 °C and source temperature of 120 °C. The TOF was calibrated with sulfadimethoxine [M]^+^ (*m*/*z* = 311.08) and leucine encephalin [M]^+^ (*m*/*z* = 556.28).

### UV–visible analysis

The UV–visible spectra of compounds I–III and aminoferrocene were recorded using a Shimadzu UV-1900i spectrophotometer at room temperature in the range of 400–800 cm^−1^ by the absorbance method.

### SEM

SEM images were obtained with a Zeiss LEO Gemini 1525 field-emission-gun scanning electron microscope equipped with an InLens detector using an accelerating voltage of 5 kV, standard aperture of 30 µm and working distance of 6.4 mm. The perovskite-coated substrates were analysed as follows: top view images were obtained using a Gemini 1 Zeiss Sigma 300 SEM microscope with a working voltage of 2.5–5 kV. Samples were coated with 15 nm of Cr by sputtering deposition to passivate the surface against oxidation and prevent charging. A silver paste was used to electrically ground samples. Cross-sectional images were collected under the same conditions, but the samples were prepared by a different method: using a diamond pin, the films were bisected and fixed to a sample holder using carbon tape such that the beam was normal to the sample cross-section.

### BET analysis

N_2_ adsorption–desorption analysis was performed using a NOVAtouch analyser with Quantachrome TouchWin software to determine the specific surface area. Before measurements, all samples were degassed at 150 °C for 2 h. Specific surface areas were calculated by applying the BET equation to the adsorption isotherm in the relative *p*/*p*_0_ range of 0.1–0.3, where *p* is the equilibrium pressure and *p*_0_ is the saturation vapor pressure at the adsorption temperature.

### Confocal microscopy

A Zeiss LSM 800 confocal laser scanning microscope (LSM) with Zen Blue 2.6 software was applied to characterize ITO films on FTO-coated glass. Confocal reflection LSM stacks were obtained using a 405 nm laser (2.0% power) with a ×20 and numerical aperture (NA) 0.7 objective lens. The collected stacks were then processed using ConfoMap software to obtain the 3D view image and determine the film thickness of the electrodes.

### AFM

AFM images were obtained using an Agilent AFM 5500 instrument in tapping mode under ambient conditions at room temperature and a Bruker NCHV probe (non-reflective) with a frequency of ~320 kHz and a spring constant of ~40 N m^−1^. Each image was generated from 512 scan lines. Images were processed in PicoView 1.5.

### XPS

XPS spectra were recorded using a Thermo Scientific K-alpha^+^ XPS spectrometer equipped with a monochromated microfocused Al Kα X-ray source (*hν* = 1,486.6 eV) and a flood gun under an operating pressure of 2 × 10^−7^ mbar. The X-ray gun power was set to 72 W (6 mA and 12 kV) with a spot size of 400 µm. Survey spectra were obtained using a pass energy of 200 eV, step size of 0.5 eV, dwell time of 25 ms and three scan accumulations. High-resolution Fe 2*p* core-level spectra were obtained using a pass energy of 20 eV, step size of 0.1 eV, dwell time of 50 ms and 60 scans. XPS quantification and deconvolution were conducted with Avantage 5.9931 software using a ‘smart’ baseline function and a Gaussian–Lorentzian ‘convolve’ function.

### Reporting summary

Further information on research design is available in the Nature Portfolio Reporting Summary linked to this article.

## Online content

Any methods, additional references, Nature Portfolio reporting summaries, source data, extended data, supplementary information, acknowledgements, peer review information; details of author contributions and competing interests; and statements of data and code availability are available at 10.1038/s41557-025-02034-0.

## Supplementary information

Supplementary Information Synthesis and characterization of molecular wires, Electrochemistry experiments for compounds I–III, Temperature dependence investigations, Theoretical basis for the mechanistic study, Electrode surface characterizations, Perovskite film and optoelectronic characterizations and Quantum chemical calculations.

Reporting Summary

## Source data

Source Data Fig. 1 The cdx file for functionalization of compounds I–III and aminoferrocene.

Source Data Fig. 2 The excel file for electrochemical characterization of surface-anchored ferrocene-derived molecular wires.

Source Data Fig. 2f top and bottom TIF files for Fig. 2f SEM (top) and AFM (bottom) images.

Source Data Fig. 3 The excel file for the mechanistic investigation of the electron transfer mechanism on hierarchical ITO electrodes.

Source Data Fig. 4 The excel file for the energy levels of compounds I–III and aminoferrocene.

Source Data Fig. 5 Separate TIF files are given for each unprocessed SEM image of the top view (including inset) and cross section of the S-ITO and S-ITO/compound I images. 5g_XRD is the raw XRD data for the diffraction patterns of perovskite grown on S-ITO and S-ITO/compound I.

Source Data Fig. 6 The data are separated into tabs whose ID correspond to letters of the figure; **b** and **c** are the *J*–*V* curves and EQE plots respectively, **d**–**g** are the statistical photovoltaic parameters of the devices prepared with compound I, PEDOT:PSS and MeO-2PACz. For the devices comparing CAP versus compound I, **i** is the *J*–*V* curves, **j** is the Nyquist plot and **k** is the Bode plot.

Source Data Extended Data Fig. 1 The excel file for the CV of compound I on S-ITO and IO-mesoITO.

Source Data Extended Data Fig. 2 The excel file for the electrochemical dataset of compound I on S-ITO at 20 °C.

Source Data Extended Data Fig. 3 The excel file for the Arrhenius plot of compound I on S-ITO.

Source Data Fig. 5 Separate TIF files are given for each unprocessed SEM image of the top view (including inset) and cross section of the S-ITO and S-ITO/compound I images. 5g_XRD is the raw XRD data for the diffraction patterns of perovskite grown on S-ITO and S-ITO/compound I.

## Data Availability

The data supporting the findings of this study are available from the Imperial Research Data repository at 10.14469/hpc/15577. Source data are provided with this paper.

## Extended data

**Extended Data Table 1:** Summary of the thermodynamic and kinetic parameters of aminoferrocene and Compounds I-III

	Thermodynamic parameters					Kinetic parameters				
	Mole-cular length (nm)	*E*_1/2_ *vs*. SHE (V)	Δ*E* (V)	*i*_*pa*_/*i*_*pc*_	*Γ* (molecules/nm^2^)	Critical scan rate (mV/s)	*α*	*β*	*k*_*app,A*_ (s^−1^)	*k*_*app,C*_ (s^−1^)
Amino-ferrocene	0.93	0.059	-0.17	1.04	0.046	190	0.15	0.13	0.91	1.18
Compound I	1.61	0.063	-0.20	1.10	0.051	157	0.13	0.12	0.66	0.87
Compound II	2.29	0.051	-0.22	1.00	0.069	123	0.14	0.11	0.52	0.71
Compound III	2.98	0.037	-0.29	0.89	0.034	90	0.13	0.12	0.37	0.50

The molecular lengths were calculated from DFT-optimized structures (BP86/def2-TZVP/D3BJ). *i*_pa_ and *i*_pc_ are the anodic and cathodic peak currents, respectively. α and β are the electron transfer coefficients, and α + β = 1 should be satisfied in an ideal scenario; ${k}_{{app},A}$ and ${k}_{{app},C}$ are the anodic and cathodic electron transfer rate constants, respectively. See Eq. 2 - Eq. 7 in Methods for Equations used to calculate these parameters.

**Extended Data Table 2:** Comparison of the kinetics and mechanism of electron transfer (ET) of Compound I on inverse-opal mesoporous indium tin oxide (IO-mesoITO) versus sputtered indium tin oxide (S-ITO)

Material	Surface loading (nmol)	Critical scan rate *Vc* (mVs^−1^)	α	β	*k*_app,A_ (s^−1^)	*k*_app,C_ (s^−1^)	Temperature dependence of *k*_app_	Activation energy (eV)
IO-mesoITO	6.2	157	0.13	0.12	0.66	0.87	yes	0.16
S-ITO	0.8	1389	0.21	0.10	6.11	5.46	yes	0.21

*α* and *β* are the electron transfer coefficients at room temperature. ${k}_{{app},A}$ and ${k}_{{app},C}$ are the anodic and cathodic electron-transfer rate constants, respectively. See also Extended Data Table 1.
