# Supplementary material for: Electron hopping in conjugated molecular wires with application to solar cells
Source: Nat Chem. 2026 Feb 9;18(4):756–64. doi: 10.1038/s41557-025-02034-0 (PMC13061622; doi:10.1038/s41557-025-02034-0)
Supplement: Supplementary file 1 — Synthesis and characterization of molecular wires, Electrochemistry experiments for compounds I–III, Temperature dependence investigations, Theoretical basis for the mechanistic study, Electrode surface characterizations, Perovskite film and optoelectronic characterizations and Quantum chemical calculations. [file 41557_2025_2034_MOESM1_ESM.pdf]

nature chemistry

Supplementary information

https://doi.org/10.1038/s41557-025-02034-0

Electron hopping in conjugated molecular wires with application to solar cells

In the format provided by the authors and unedited

Contents

1 Synthesis 2

1.1 General procedure for Sonogashira coupling 3

1.2 General procedure for deprotection 3

1.3 Synthesis of molecular wires 3

2 NMR spectroscopy 14

3 UV-vis data for the compound series 35

4 Electrochemistry for aminoferrocene and compounds I to III 36

5 Electrochemical Kinetics Calculations (Compound II as an example) 40

6 Temperature dependence study 41

6.1 Influence of peak separation threshold on the β value 41

6.2 Temperature-dependence study of electron transfer mechanism 44

6.2.1 Temperature-dependence study of Compound I on IO-mesoITO 47

6.2.2 Temperature-dependence study of Compound I on S-ITO 55

7 Surface Characterisation 61

7.1 Atomic Force Microscopy (AFM) 61

7.2 X-ray photoelectron spectroscopy (XPS) 62

7.3 BET (Brunauer-Emmett-Teller) measurements and surface coverage estimates 65

7.4 Confocal microscopy 66

7.5 Kelvin Probe (KP) measurements 67

8 Characterisation of molecular wires on S-ITO – contact angles & absorbance 67

9 Characterisation of perovskite films deposited on S-ITO & S-ITO/Compound I 68

10 Electronic characterisation of perovskite devices 70

11 Quantum Chemical Calculations 72

11.1 Density Functional Theory 72

11.2 Example Input Files 72

11.3 XYZ coordinates for ORCA calculations 73

12 References 81

1 Synthesis

Although the synthesis of oligo(p-phenylene ethynylene)-based molecular wires (OPE) is well-documented, simultaneously achieving the modular addition of several aryl units with ultimate control over total molecular length, the position of each unit and functionalization type has not yet been demonstrated. Here we show a modified synthetic strategy to achieve these goals, developing the use of orthogonal protecting groups at each stage of assembling the molecular wire architecture. Ferrocene-appended OPE-based molecular wires Compounds I-III (Figure 1), and aminoferrocene were characterized by NMR, mass spectrometry and ultraviolet-visible spectroscopy (see Supplementary Sections 2, 3 and 4).

Our strategy enabled us to synthesize a series of OPE molecular wires from 1-3 aryl units, terminated with the protecting groups trimethylsilane (TMS) and tri-isopropylsilyly-ethynyl (TIPS), in good yield. With the OPE wires protected at both ends, the TMS group was removed by alkaline hydrolysis, exposing the alkyne group, which was immediately coupled to trifluoroacetamide protected 4-iodoaniline. Subsequently, the TIPS group was removed with tetrabutylammonium fluoride (t-BuN$^+$F$^-$), such that iodoferrocene could be coupled to the OPE wire. Subsequent treatment with a strong base yielded the free -NH_2 and ferrocene-derived molecular wires (Compounds I-III).

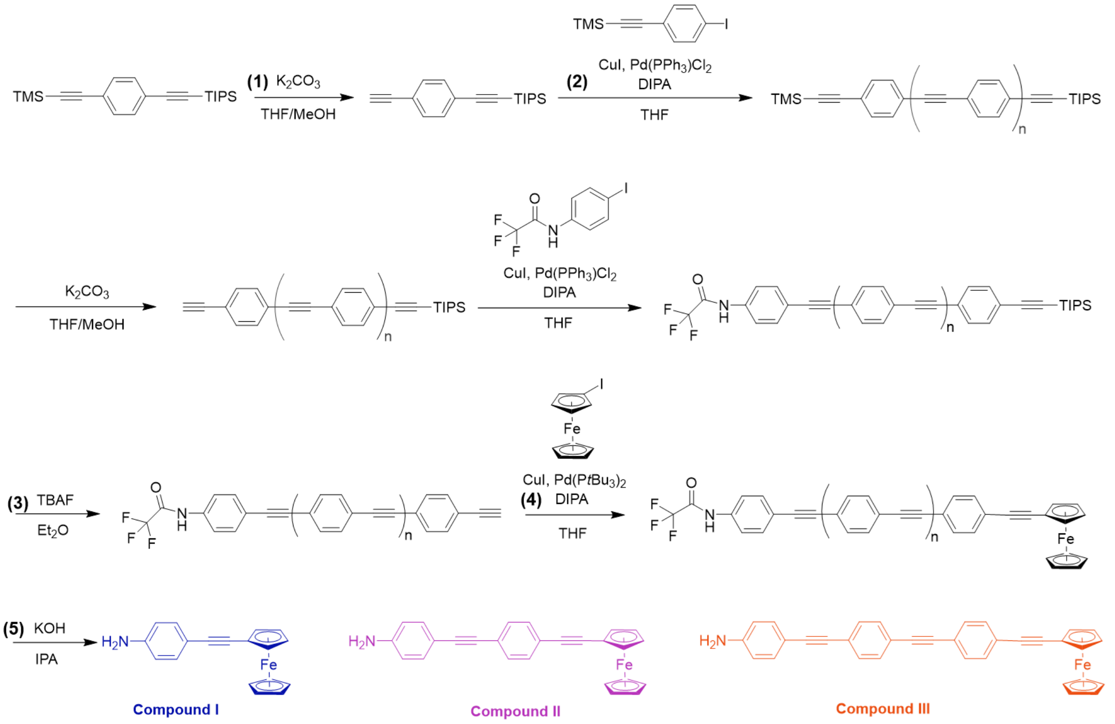

Figure 1. The general synthetic route of compounds I-III. **Reagent and conditions:** (1) K_2CO_3 (2 equiv), THF/MeOH (1:1), room temperature (rt), overnight, yield 80%; (2) copper iodide (0.1 equiv), bis(triphenylphosphine)palladium(II) dichloride (0.1 equiv), DIPA ,THF reflux overnight under nitrogen, yield 60%; (3) 1M solution of tetrabutylammonium fluoride in THF (1.5 equiv), rt, 15 min (4) copper iodide (0.2 equiv), bis(tri-*tert*-butylphosphine)palladium(0) (0.2 equiv),

DIPA, THF reflux overnight under nitrogen, yield 14% (2 steps) (5) KOH (2.5 equiv), i-PrOH, reflux 2.5 h, yield 60%.

1.1 *General procedure for Sonogashira coupling*

All the reagents were dried and all the solvents were degassed before use. To a pre-heated round bottom flask, alkyne (1eq), aryl halide (1-1.2 eq), bis(triphenylphosphine)palladium(II) chloride ($Pd(PPh_3)_2Cl_2$) (5 mol% per alkyne) and copper(I) iodide (CuI) (5 mol% per alkyne) were added. The flask was connected to Schlenk line following the addition of dry THF and degassed Diisopropylamine(DIPA). The mixture was stirred under N_2 at 60-70 ℃ overnight. After the completion of reaction, the mixture was washed with H_2O twice, the organic phase was collected, dried over anhydrous Na_2SO_4 after every water/DCM liquid extraction and then dried *in vacuo*. The crude product was purified through column chromatography on a short silica column to yield pure compound.

1.2 *General procedure for deprotection*

The silylated alkyne was dissolved in methanol/THF 1:1 in a round bottom flask, potassium carbonate K_2CO_3 (2 eq) was added portion-wise. The mixture was stirred at room temperature overnight. After the completion of reaction, the mixture was dried *in vacuo* and diluted with DCM (100 mL) and an aqueous extraction was performed by first adding water (200 mL) and extracting the organic phase. The organic phase was then washed with water (3 x 200 mL) and then brine (200 mL). The organic phase was dried over anhydrous Na_2SO_4 after every water/DCM liquid extraction. The solvent was removed *in vacuo* and the crude was purified by passing it through a silica plug using n-hexane as the eluent.

1.3 *Synthesis of molecular wires*

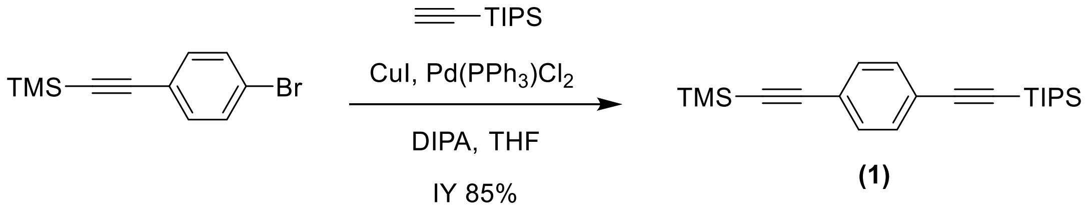

Synthesis of triisopropyl((4-((trimethylsilyl)ethynyl)phenyl)ethynyl)silane (1): ((4-bromophenyl)ethyl)trimethylsilane (4.05 g, 16 mmol), copper iodide (0.30 g, 1.6 mmol) and bis(triphenylphosphine)palladium (II) dichloride (1.12 g, 1.6 mmol) were dissolved in dry THF (100 mL) and degassed DIPA (15 mL). TIPS-acetylene (3.20 g, 2.97 mL, 17.6 mmol) was added and the mixture was refluxed under nitrogen overnight. After completion of the reaction, the mixture was allowed to cool to room temperature and the solvent was removed *in vacuo*. The mixture was purified through the use of column chromatography on a short silica column, eluting with n-hexane. The solvent was removed *in vacuo* to give the compound 1 as colorless oil (yield 5.50 g, 15 mmol, 93%).

R*f* 0.9 (hexane,UV)

^{1}H NMR ($CDCl_3$, 298 K, 400 MHz): δ 7.39 (s, 4H), 1.13 (s, 21H), 0.25 (s, 9H).

^{13}C NMR ($CDCl_3$, 298 K, 400 MHz): δ 131.96, 131.86, 123.70, 123.09, 106.72, 104.78, 96.28, 92.93, 18.81, 11.45, 0.07

MS (ESI$^+$) (MeCN): *m/z* calcd. for $C_{22}H_{34}Si_2$ *m/z* 354.2194; found 354.2182

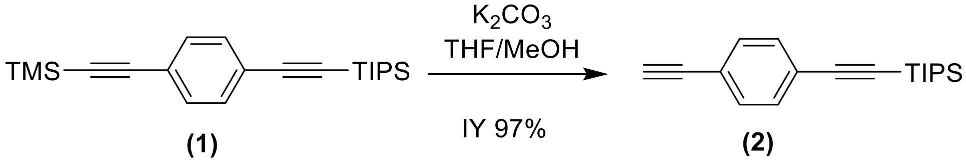

(1) **(2)**

Synthesis of ((4-ethynylphenyl)ethynyl)triisopropylsilane (2): Triisopropyl((4-((trimethylsilyl)ethynyl)phenyl)ethynyl)silane (**1**) (5.5 g, 15 mmol) was dissolved in a 1:1 mixture of MeOH:THF (100 mL). K_2CO_3 (2.07 g, 30 mmol) was added, and the mixture was stirred at room temperature overnight. Upon completion the mixture was dried *in vacuo* and diluted with DCM (100 mL) and an aqueous extraction was performed by first adding water (200 mL) and extracting the organic phase. The organic phase was then washed with water (3 x 200 mL) and then brine (200 mL). The organic phase was dried over anhydrous Na_2SO_4 after every water/DCM liquid extraction. The solvent was removed *in vacuo* and the crude was purified by passing it through a silica-plug using n-hexane as the eluent. The solvent was removed *in vacuo* to give compound **2** as a white solid (3.70 g, 14.6 mmol, 97%).

R*f* 0.9 (hexane, UV)

^{1}H NMR ($CDCl_3$, 298 K, 400 MHz): δ 7.43 (s, 4H), 3.17 (s, 1H), 1.13 (s, 21H)

^{13}C NMR ($CDCl_3$, 298 K, 400 MHz): δ 132.04, 124.12, 122.07, 106.54, 93.10, 83.40, 79.00, 18.80, 11.44

MS (ESI$^+$) (MeCN): *m/z* calcd. for $C_{19}H_{26}Si$ $[M+H]^+$ *m/z* 283.1877; found 283.1881

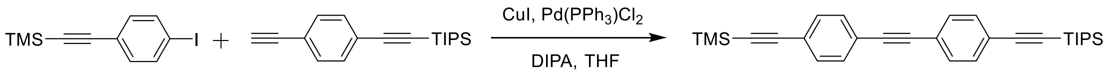

(2) **(3)**

Synthesis of triisopropyl ((4-((4-((trimethylsilyl) ethynyl) phenyl) ethynyl) phenyl)ethynyl)silane (3): ((4-Ethynylphenyl)ethynyl)triisopropylsilane (**2**) (250 mg, 0.89 mmol), (4-Iodophenylethynyl) trimethylsilane (268 mg, 0.89 mmol), copper iodide (17 mg, 0.09 mmol) and bis(triphenylphosphine)palladium(II) dichloride (63 mg, 0.09 mmol) were dissolved in dry THF (20 mL). Degassed DIPA (5 mL) was then added and the resulting solution was refluxed overnight. Upon completion, the reaction solution was allowed to cool to room temperature and the solvent was removed *in vacuo*. The mixture was diluted with DCM (30 mL) and washed with water (30 mL) and brine (30

mL). The organic phase was dried over anhydrous Na_2SO_4 after every water/DCM liquid extraction. The organic phase was collected and concentrated. The mixture was purified through the use of column chromatography on a short silica column, eluting with n-hexane. The solvent was removed *in vacuo* to give the compound **3** as light-yellow solid (yield 300 mg, 0.66 mmol, 74%).

R*f* 0.6 (10% EtOAc in hexane, UV)

^{1}H NMR ($CDCl_3$, 298 K, 400 MHz): δ 7.45 (s, 8H), 1.14 (s, 21H), 0.26 (s, 9H)

^{13}C NMR ($CDCl_3$, 298 K, 400 MHz): δ 132.15, 131.54, 123.70, 123.25, 123.21, 123.97, 106.73, 104.73, 96.57, 93.11, 91.14, 90.95, 18.81, 11.45, 0.06

MS (ESI$^+$) (MeCN): *m/z* calcd. for $C_{30}H_{38}Si_2$ $[M+H]^+$ *m/z* 455.2585; found 455.2583

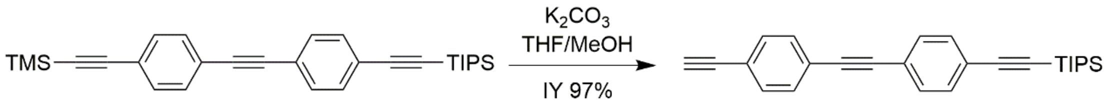

(3) (4)

Synthesis of ((4-((4-ethynylphenyl)ethynyl)phenyl)ethynyl)triisopropylsilane (4): Triisopropyl((4-((4-((trimethylsilyl)ethynyl)phenyl) ethynyl) phenyl)ethynyl)silane (**3**) (5.50 g, 15 mmol) was dissolved in a 1:1 mixture of MeOH:THF (100 mL). K_2CO_3 (2.07 g, 30 mmol) was added, and the mixture was stirred at room temperature overnight. Upon completion, the solvents were removed *in vacuo* and the crude mixture was redissolved in DCM (100 mL). The DCM solution was then washed with water (4 x 200 mL) followed by brine (200 mL). The organic phase was dried over anhydrous Na_2SO_4 after every water/DCM liquid extraction. The organic phase was isolated and the solvent was removed *in vacuo* and the crude product was purified by passing it through a silica-plug using n-hexane as the eluent. The solvent was removed *in vacuo* to give compound **2** as a white solid (3.70 g, 14.6mmol, 97%).

R*f* 0.5 (10% EtOAc in hexane, UV)

^{1}H NMR ($CDCl_3$, 298 K, 400 MHz): δ 7.48 (s, 4H), 7.47 (s, 4H), 3.19 (s, 1H), 1.15 (s, 21H)

^{13}C NMR ($CDCl_3$, 298 K, 400 MHz): δ 132.61, 132.17, 131.75, 131.57, 124.16, 123.87, 122.80, 121.71, 106.69, 93.27, 92.01, 90.78, 82.28, 75.86, 18.81, 11.45

MS (ESI$^+$) (MeCN): *m/z* calcd. for $C_{27}H_{30}Si$ $[M+H]^+$ *m/z* 383.2190; found 383.2194

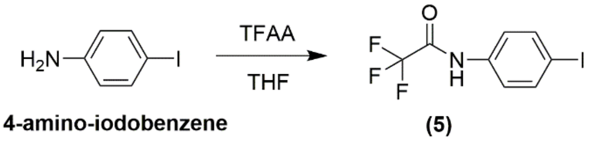

4-amino-iodobenzene **(5)**

Synthesis of 2,2,2-trifluoro-N-(4-iodophenyl) acetamide (5): 4-Iodoaniline (2.19 g, 10 mmol, 1 eq) was dissolved in the dry THF (20 ml), trifluoroacetic anhydride (4.20 g, 20 mmol, 2 eq) was added dropwise to the solution at 0~2 °C within 15 minutes and the solution was further stirred for 1 hour at RT. After the completion, the reaction was evaporated to dryness and the result residue was stirred with H_2O twice. The participant was filtered and dried at 50 °C *in vacuo*. The product was afforded as off-white flakes (3.10 g, 9.8mmol, 98%).

R*f* 0.9 (hexane, UV)

^{1}H NMR ($CDCl_3$, 298 K, 400 MHz): δ 7.85 (s, 1H), 7.71 (d, *J* = 12 Hz, 2H), 7.35 (d, *J* = 8 Hz, 2H)

^{13}C NMR ($CDCl_3$, 298 K, 400 MHz): δ 138.57, 134.95, 122.31,117.16,114.29, 90.44

^{19}F NMR (CDCl3, 298 K, 400 MHz): δ -75.66

MS (ESI$^+$) (MeCN): *m/z* calcd. for $[C_8H_5F_3INO\ -H]^-$: 313.9295; found 313.9288

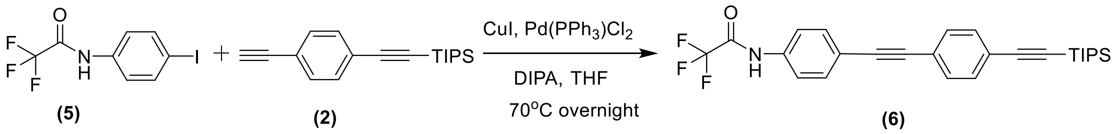

Synthesis of 2,2,2-trifluoro-N-(4-((4-((triisopropylsilyl) ethynyl) phenyl) ethynyl) phenyl) acetamide (6): ((4-Ethynylphenyl)ethynyl)triisopropylsilane (2) (300 mg, 1.1 mmol), 2,2,2-trifluoro-N-(4-iodophenyl) acetamide (5) (402 mg, 1.2 mmol), copper iodide (19 mg, 0.1 mmol) and bis(triphenylphosphine)palladium(II) dichloride (70 mg, 0.1 mmol) were added in round-bottom flask and connected to a Schlenk line following the addition of dry THF (20 mL) and degassed diisopropylamine(DIPA) (5 mL). The mixture was refluxed overnight. After completion of the reaction, the mixture was allowed to cool to room temperature and the solvent was removed *in vacuo*. The mixture was diluted in DCM (30 mL) and washed with water (30 mL) and brine (30 mL). The organic phase was dried over anhydrous Na_2SO_4 after every water/DCM liquid extraction. The organic layer was collected and concentrated. The mixture was purified via column chromatography on a short silica column, eluting with n-hexane. The solvent was removed *in vacuo* to give the compound 6 as light-yellow solid (yield 360 mg, 0.77 mmol, 70%).

R*f* 0.6 (20% EtOAc in hexane, UV)

^{1}H NMR ($CDCl_3$, 298 K, 400 MHz): δ 7.97 (s, 1H), 7.56 (d, *J* = 12 Hz, 4H), 7.46 (s, 4H), 1.15 (s, 21H)

^{13}C NMR ($CDCl_3$, 298 K, 400 MHz): δ 154.99, 154.61, 135.18, 132.82, 132.15, 131.51, 123.71, 122.90, 121.24, 120.33, 117.17, 114.30, 106.70, 93.14, 90.32, 90.01, 77.48, 77.16, 76.85, 53.57, 18.81, 11.44.

^{19}F NMR (CDCl3, 298 K, 400 MHz): δ -75.64

MS (ESI$^+$) (MeCN): *m/z* calcd. for $C_{27}H_{31}NOSi$ $[M+H]^+$ *m/z* 470.2122; found 470.2132

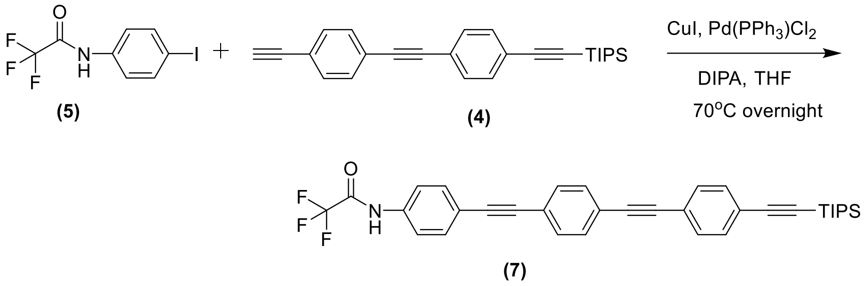

Synthesis of 2,2,2-trifluoro-N-(4-((4-((4-((triisopropylsilyl) ethynyl) phenyl) ethynyl) phenyl) ethynyl) phenyl)acetamide (7): ((4-((4-Ethynylphenyl)ethynyl)phenyl)thynyl) triisopropylsilane (4) (252 mg, 0.66 mmol,1 eq), 2,2,2-trifluoro-N-(4-iodophenyl) acetamide (5) (252 mg, 0.79 mmol,1.2 eq), copper iodide (13 mg, 0.07 mmol, 0.1 eq) and bis(triphenylphosphine)palladium(II) dichloride (50 mg, 0.07 mmol, 0.1 eq) were added in round-bottom flask and connected to Schlenk line following the addition of dry THF (20 mL) and degassed diisopropylamine (DIPA) (5mL). The mixture was refluxed overnight. After completion of the reaction, the mixture was allowed to cool to room temperature and the solvent was removed in vacuo. The mixture was diluted in DCM (30 mL) and washed with water (30 mL) and brine (30 mL). The organic phase was dried over anhydrous Na_2SO_4 after every water/DCM liquid extraction. The organic layer was collected and concentrated. The mixture was purified via column chromatography on a short silica column, eluting with n-hexane. The solvent was removed *in vacuo* to give the compound 6 as light-yellow solid (yield 230 mg, 0.41mmol, 62%).

R*f* 0.5 (20% EtOAc in hexane, UV)

^{1}H NMR ($CDCl_3$, 298 K, 400 MHz): δ 7.87 (s, 1H), 7.59 (d, *J* = 4Hz, 4H), 7.49 (d, *J* = 20Hz, 8H), 1.13 (s,21H)

^{13}CNMR($CDCl_3$,298K,400MHz):δ138.57,135.20,132.86,132.17,131.74,131.71,131.54, 123.74,123.23, 122.94, 122.29, 121.21, 120.32, 93.17, 91.27, 90.95, 90.51,18.81, 14.28, 11.45

^{19}F NMR (CDCl3, 298 K, 400 MHz): δ -75.64

MS (ESI$^+$) (MeCN): *m/z* calcd. for $C_{35}H_{34}F_3NOSi$ *m/z* 570.2435; found 570.2439

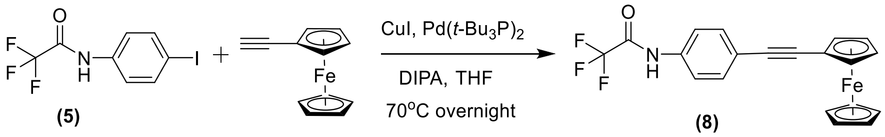

Synthesis of compound (8): Ethynyferrocene (420 mg, 2 mmol,1 eq), 2,2,2-trifluoro-N-(4-iodophenyl) acetamide (5) (945 mg, 3 mmol,1.5 eq), copper iodide (57 mg, 0.3

mmol, 0.15 eq) and bis(tri-*tert*-butylphosphine) palladium(0) (153 mg, 0.3 mmol, 0.15 eq) were added in round-bottom flask and connected to Schlenk line following the addition of dry THF (30 mL) and degassed diisopropylamine (DIPA) (10 mL). The mixture was refluxed overnight. After completion of the reaction, the mixture was allowed to cool to room temperature and the solvent was removed *in vacuo*. The mixture was diluted in DCM (30 mL) and washed with water (30 mL) and brine (30 mL). The organic phase was dried over anhydrous Na_2SO_4 after every water/DCM liquid extraction. The organic layer was collected and concentrated. The mixture was purified through the use of column chromatography on a short silica column, eluting with n-hexane. The solvent was removed *in vacuo* to give compound 6 as light-yellow solid (yield 400 mg, 1 mmol, 50%).

R*f* 0.5 (20% EtOAc in hexane, UV)

^{1}H NMR ($CDCl_3$, 298 K, 400 MHz): δ 7.85 (s,1H), 7.53 (q, *J* = 8Hz, 4H), 4.50 (s,2H), 4.25 (s, 7H),

^{19}F NMR (CDCl3, 298 K, 400 MHz): δ -75.64

MS (ESI$^+$) (MeCN): *m/z* calcd. for $C_{20}H_{14}F_3FeNO$ *m/z* 397.0377; found 397.0377

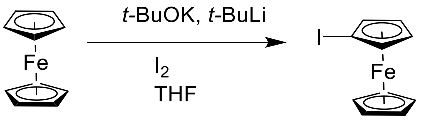

(9)

Synthesis iodoferrocene (9): Ferrocene (7.45 g, 40 mmol), potassium *tert*-butoxide (0.54 g, 4.8 mmol) was dissolved in 100 ml dry THF in a Schlenk flask under N_2 protection. The solution was cooled to -78 °C, and *tert*-butyllithium (32 ml, 80 mmol) was added dropwise. After stirring at -78 °C for 30 min, orange precipitate was formed and stirring was continued for 1.5 h. Then solid iodine (12.7 g, 50 mmol) was added to the system, and then the system was warmed to RT. After completion, the reaction mixture was washed with saturated sodium thiosulfate (200 ml) and diluted with hexane. The organic phase was dried over anhydrous Na_2SO_4 after every water/DCM liquid extraction. The organic phase was collected and concentrated. The dark oil crude was filtered through a short silica column, and iodoferrocene (9) was obtained as brick red solid (yield 9.8 g, 31.5 mmol, 78%).

R*f* 0.95 (hexane, UV)

^{1}H NMR ($CDCl_3$, 298 K, 400 MHz): δ 4.42 (s, 2H), 4.20 (s, 5H), 4.16 (s, 2H)

^{13}C NMR ($CDCl_3$, 298 K, 400 MHz): δ 74.67, 71.26, 69.04

MS (ESI$^+$) (MeCN): *m/z* calcd. for $C_{10}H_9FeI$ *m/z* 311.9098; found 311.9112

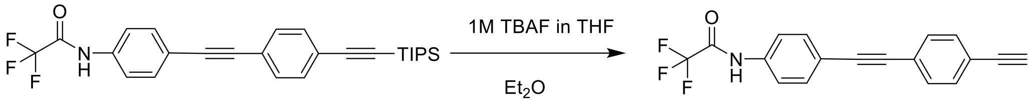

(6) **(10)**

Synthesis of N-(4-((4-ethynylphenyl)ethynyl)phenyl)-2,2,2-trifluoroacetamide (10): 2,2,2-trifluoro-N-(4-((4-((triisopropylsilyl)ethynyl)phenyl)ethynyl)phenyl) acetamide (6) (669 mg, 1.42 mmol) was dissolved in dry diethyl ether (10 mL), when stirring, 1M solution of tetrabutylammonium fluoride in THF (2.3 ml, 2.3 mmol, 1.5 eq) was added dropwise and stirred about 5 min. After completion, the mixture was concentrated and diluted by DCM (30 mL), the organic solution was washed with water (30 mL) and brine (30 mL). The organic phase was dried over anhydrous Na_2SO_4 after every water/DCM liquid extraction. The organic phase was collected, concentrated and located on a short silica gel column to purify. The compound **10** was got as white solid. (Yield 310 mg, 1 mmol, 70%).

R*f* 0.4 (20% EtOAc in hexane, UV)

^{1}H NMR ($CDCl_3$, 298 K, 400 MHz): δ 7.93 (s, 1H), 7.64-7.58 (m, 4H), 7.50 (s,4H), 3.21 (s,1H).

^{19}F NMR (CDCl3, 298 K, 400 MHz): δ -75.63

MS (ESI$^+$) (MeCN): *m/z* calcd. for $C1_8H_{10}F_3NO$ *m/z* 313.07; found 313.2739

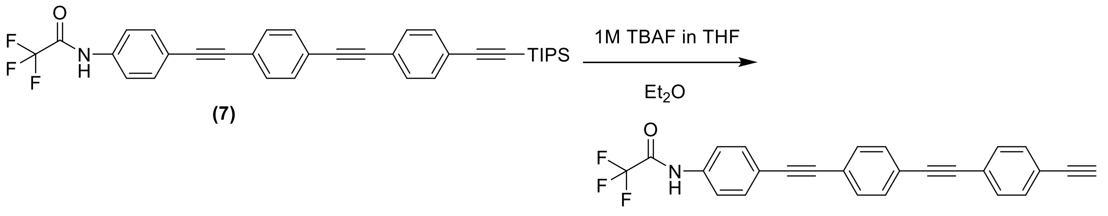

(7) **(11)**

Synthesis of N-(4-((4-((4-ethynylphenyl) ethynyl) phenyl)ethynyl) phenyl)-2,2,2-trifluoroacetamide (11): 2,2,2-Trifluoro-N-(4-((4-((4-((triisopropylsilyl)ethynyl) phenyl)ethynyl)phenyl)ethynyl)phenyl)acetamide (7) (100 mg, 0.18 mmol, 1 eq) was dissolved in dry diethyl ether (10 mL), when stirring, 1M solution of tetrabutylammonium fluoride in THF (0.2 ml, 0.2 mmol, 1.2 eq) was added dropwise and stirred about 5 min. After completion, the mixture was concentrated and diluted by DCM (30 mL), the organic solution was washed with water (30 mL) and brine (30 mL). The organic phase was dried over anhydrous Na_2SO_4 after every water/DCM liquid extraction. The organic phase was collected, concentrated, and located on a short silica gel column to purify. The compound **11** was got as white solid. (Yield 40 mg, 0.1 mmol, 56%).

R*f* 0.4 (20% EtOAc in hexane, UV)

^{1}H NMR ($CDCl_3$, 298 K, 400 MHz): δ 10.53 (s, 1H), 7.85-7.65 (m, 4H), 7.62 (s, 4H), 7.60-7.57 (m,4H), 3.86 (s, 1H)

^{19}**F NMR** (CDCl3, 298 K, 400 MHz): δ -76.20

MS (ESI$^+$) (MeCN): *m/z* calcd. for $C_{26}H_{14}F_3N$-fO *m/z* 414.1100; found 414.1096

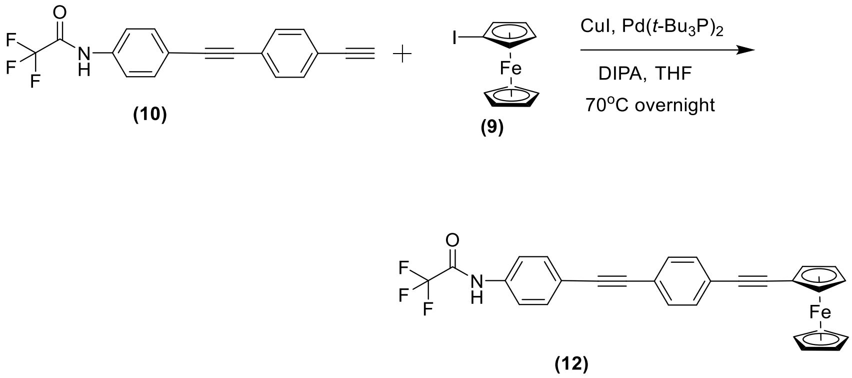

Synthesis of compound (12): Iodoferrocene (162 mg, 0.52 mmol, 2 eq), N-(4-((4-ethynylphenyl)ethynyl)phenyl)-2,2,2-trifluoroacetamide (10) (80 mg, 0.26 mmol,1 eq), copper iodide (19 mg, 0.1 mmol, 0.2 eq) and bis(tri-*tert*-butylphosphine)palladium(0) (51 mg, 0.1 mmol, 0.2 eq) were added in round-bottom flask and connected to Schlenk line following the addition of dry THF (30 mL) and degassed diisopropylamine (DIPA) (10 mL). The mixture was refluxed overnight. After completion of the reaction, the mixture was allowed to cool to room temperature and the solvent was removed in vacuo. The mixture was diluted in DCM (30 mL) and washed with water (30 mL) and brine (30 mL). The organic phase was dried over anhydrous Na_2SO_4 after every water/DCM liquid extraction. The organic layer was collected and concentrated. The mixture was purified through the using of column chromatography on a short silica column, eluting with n-hexane. The solvent was removed *in vacuo* to give the compound 6 as light-yellow solid (yield 90 mg, 0.18 mmol, 70%).

R*f* 0.3 (10% EtOAc in hexane, UV)

^{1}**H NMR** ($CDCl_3$, 298 K, 400 MHz): δ 7.88 (s, 1H), 7.61-7.55 (m, 4H), 7.49-7.44 (m, 4H), 4.52 (s,2H), 4.26 (s, 7H)

^{13}**C NMR** ($CDCl_3$, 298 K, 400 MHz): δ 132.81,131.62,131.48, 120.31, 71.79, 70.42, 69.43

^{19}**F NMR** (CDCl3, 298 K, 400 MHz): δ -75.66

MS (ESI$^+$) (MeCN): *m/z* calcd. for $C_{28}H_{18}F_3FeON^+$ *m/z* 497.0684; found 497.0678

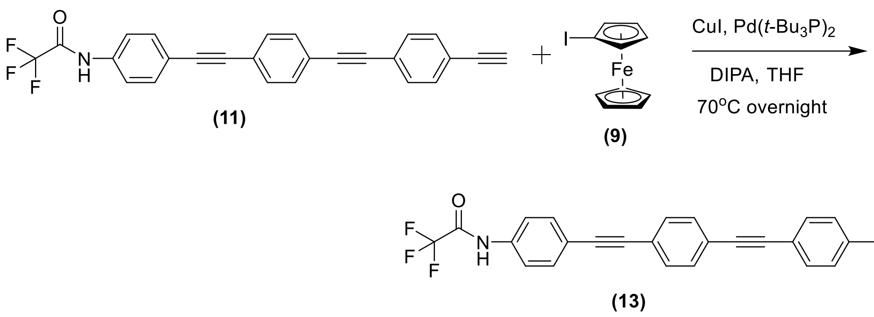

Synthesis of compound (13): Iodoferrocene (47 mg, 0.15 mmol,1.5 eq), N-(4-((4-((4-ethynylphenyl)ethynyl)phenyl)ethynyl)phenyl)-2,2,2-trifluoroacetamide(11) (40 mg, 0.1 mmol,1 eq), copper iodide (5 mg, 0.02 mmol, 0.2 eq) and bis(tri-*tert*-butylphosphine)palladium(0) (10 mg, 0.02 mmol, 0.2 eq) were added in round-bottom flask and connected to Schlenk line following the addition of dry THF (30 mL) and degassed diisopropylamine (DIPA) (10 mL). The mixture was refluxed overnight. After completion of the reaction, the mixture was allowed to cool to room temperature and the solvent was removed in vacuo. The mixture was diluted in DCM (30 mL) and washed with water (30 mL) and brine (30 mL). The organic phase was dried over anhydrous Na_2SO_4 after every water/DCM liquid extraction. The organic layer was collected and concentrated. The mixture was purified through the using of column chromatography on a short silica column, eluting with n-hexane and EtOAc. The solvent was removed *in vacuo* to give the compound 13 as light-yellow solid (yield 42 mg, 0.07 mmol, 70%).

R*f* 0.4 (20% EtOAc in hexane, UV)

^{1}H NMR ($CDCl_3$, 298 K, 400 MHz): δ 7.88 (s, 1H), 7.61-7.58 (m, 4H), 7.51 (s,4H), 7,47-7.46 (m,4H), 4.55 (s, 2H), 4.29 (s, 5H)

^{19}F NMR (CDCl3, 298 K, 400 MHz): δ -75.63

MS (ESI$^+$) (MeCN): *m/z* calcd. for $C_{36}H_{22}F_3FeNO$ *m/z* 597.10; found 597.0995

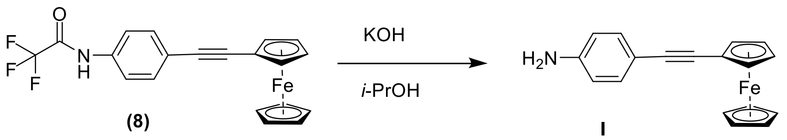

Synthesis of compound (I): The compound 8 (65 mg, 0.17 mmol, 1 eq) was dissolved in *i*-PrOH, potassium hydroxide (23 mg, 0.4 mmol, 2.5 eq) was added portion-wise. The mixture was refluxed for 2.5 h. After the completion of the reaction, the residue was evaporated to dryness at 40~45 °C, the result residue was stirred with cold hexane to remove the trace of *i*-PrOH. The product was suspension in DCM, and poured at the top of a short, cold column. The system was eluted with hexane/EtOAc, the sunset yellow filtrate was collected and evaporated to afford rusty red product (30 mg, 0.1 mmol, 59%).

R*f* 0.3 (20% EtOAc in hexane, UV)

^{1}H NMR ($CDCl_3$, 298 K, 400 MHz): δ 7.99 (s, 2H), 7.52 (s, 2H), 4.55 (s, 2H), 4.28 (s, 5H), 3.92 (s, 2H)

^{13}C NMR ($CDCl_3$, 298 K, 400 MHz): δ 131.34, 129.62, 71.98, 70.56, 69.68

MS (ESI$^+$) (MeCN): *m/z* calcd. for $C_{18}H_{15}FeN$ *m/z* 301.0554; found 301.0559

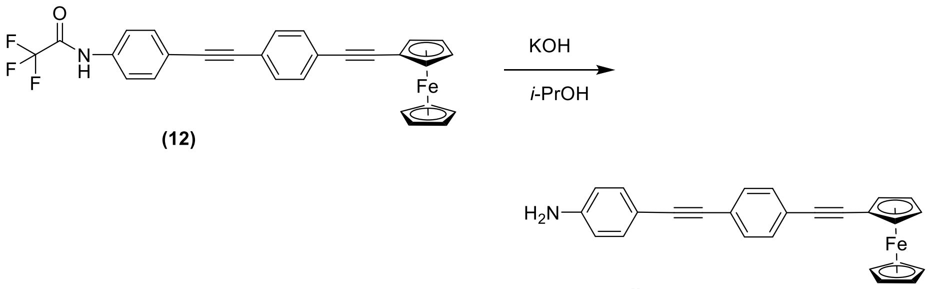

Synthesis of compound (II): The compound 12 (90 mg, 0.18 mmol, 1 eq) was dissolved in *i*-PrOH, potassium hydroxide (25 mg, 0.45 mmol, 2.5 eq) was added portion wise. The mixture was refluxed for 2.5 h. After the completion of the reaction, the residue was evaporated to dryness at 40~45 °C, the result residue was stirred with cold hexane to remove the trace of *i*-PrOH. The product was suspension in DCM, and poured at the top of a short, cold column. The system was eluted with hexane/EtOAc, the sunset yellow filtrate was collected and evaporated to afford rusty red product (23 mg, 0.1 mmol, 32%).

R*f* 0.4 (20% EtOAc in hexane, UV)

^{1}H NMR ($CDCl_3$, 298 K, 400 MHz): δ 7.42 (s, 4H), 7.33 (s, 2H), 6,65 (s, 2H), 4.54 (s,2H), 4.28 (s,7H)

^{13}C NMR ($CDCl_3$, 298 K, 400 MHz): δ 133.14, 131.37, 131.33, 123,23, 115.00, 71.61,70.18, 69.12

MS (ESI$^+$) (MeCN): *m/z* calcd. for $C_{26}H_{19}FeN$ *m/z* 401.0867; found 401.0854

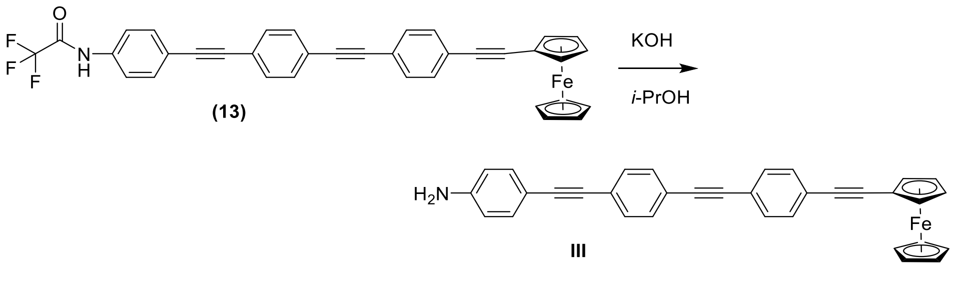

Synthesis of compound (III): The compound 13 (42 mg, 0.07 mmol, 1 eq) was dissolved in *i*-PrOH, potassium hydroxide (10 mg, 0.18 mmol, 2.5 eq) was added portion wise. The mixture was refluxed for 2.5 h. After the completion of the reaction,

the residue was evaporated to dryness at 40~45 °C, the result residue was stirred with cold hexane to remove the trace of *i*-PrOH. The product was suspension in DCM, and poured at the top of a short, cold column. The system was eluted with hexane/EtOAc, the sunset yellow filtrate was collected and evaporated to afford rusty red product (20 mg, 0.04 mmol, 57%).

Rf 0.4 (20% EtOAc in hexane, UV)

^{1}H NMR ($CDCl_3$, 298 K, 400 MHz): δ 7.47-7.45 (m, 8H), 7.34 (d, *J* = 8Hz, 2H), 6.65 (d, *J* = 8Hz, 2H), 5.30 (s, 2H), 4.55 (s, 2H), 4.29 (s, 5H)

MS (ESI$^+$) (MeCN): *m/z* calcd. for $C_{34}H_{23}FeN$ *m/z* 501.1180; found 501.1190.

2 NMR spectroscopy

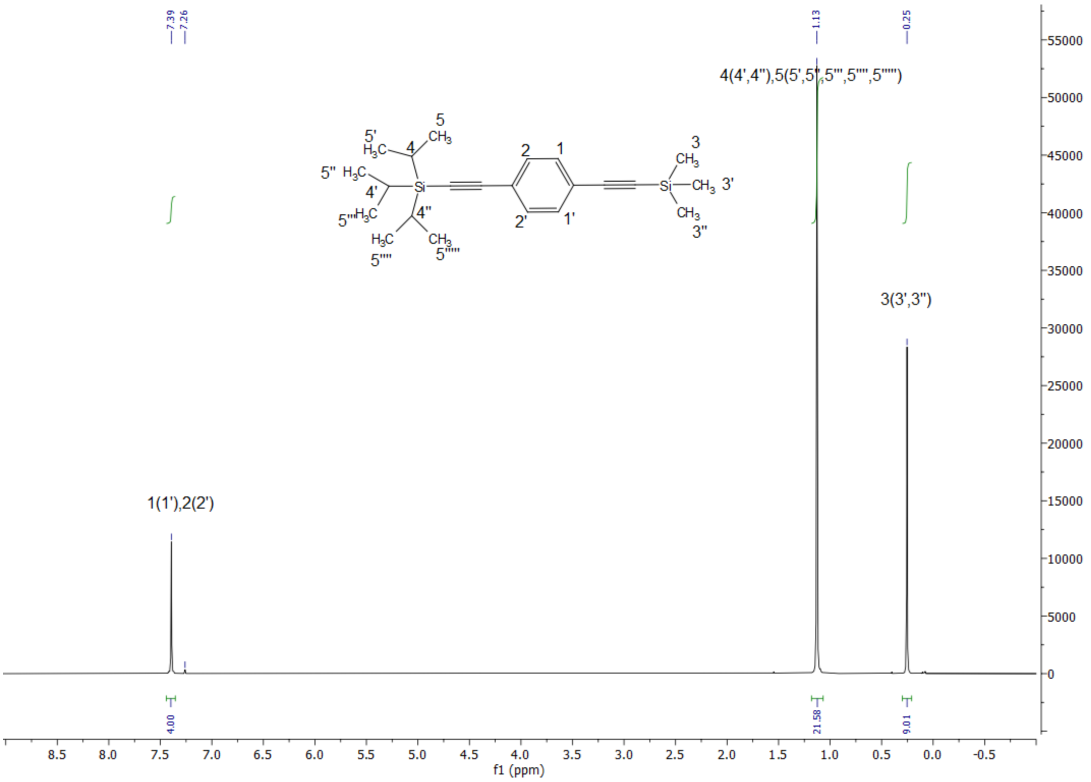

Figure N1: ^{1}H NMR for compound 1 in $CDCl_3$, 400 MHz.

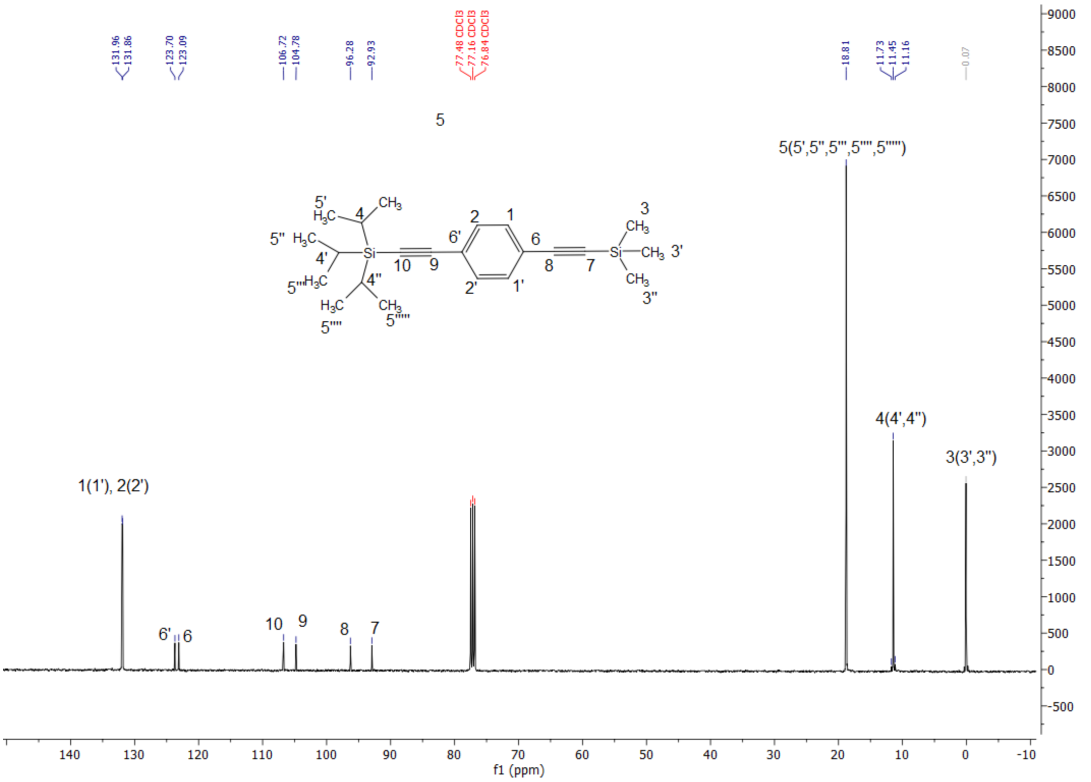

Figure N2: ^{13}C NMR for compound 1 in $CDCl_3$, 400 MHz.

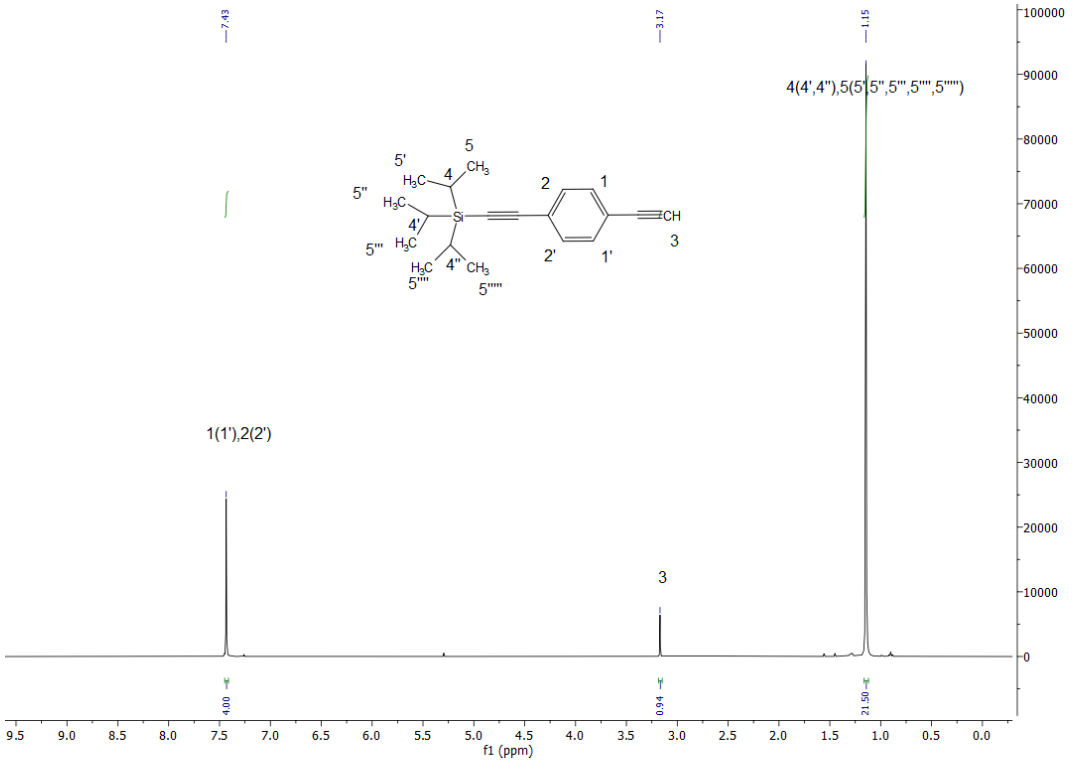

Figure N3: ^{1}H NMR for compound 2 in $CDCl_3$, 400 MHz.

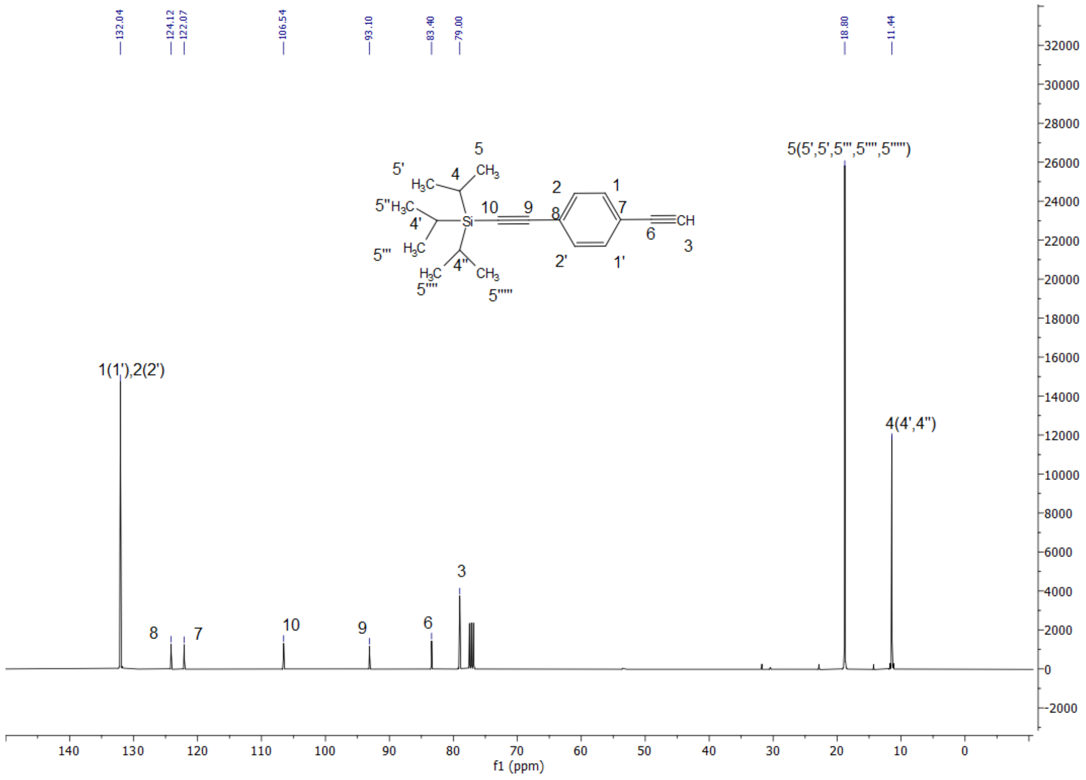

Figure N4: ^{13}C NMR for compound 2 in $CDCl_3$, 400 MHz.

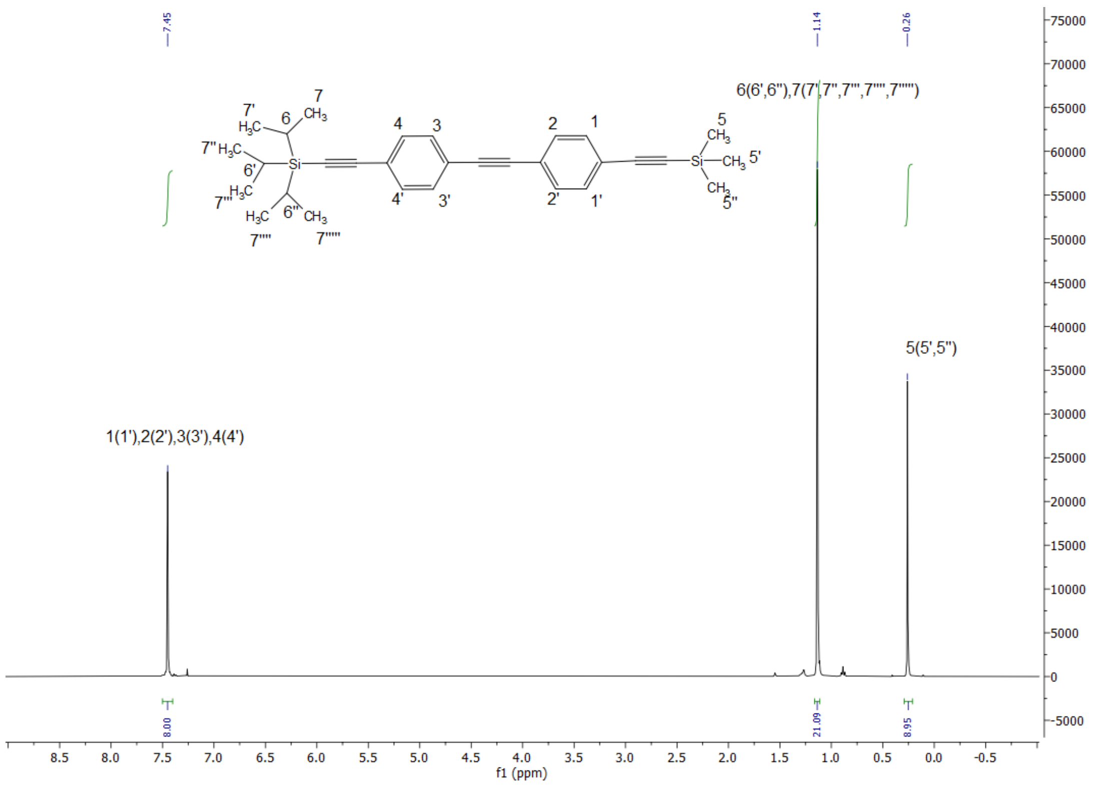

Figure N5: ^{1}H NMR for compound 3 in $CDCl_3$, 400 MHz.

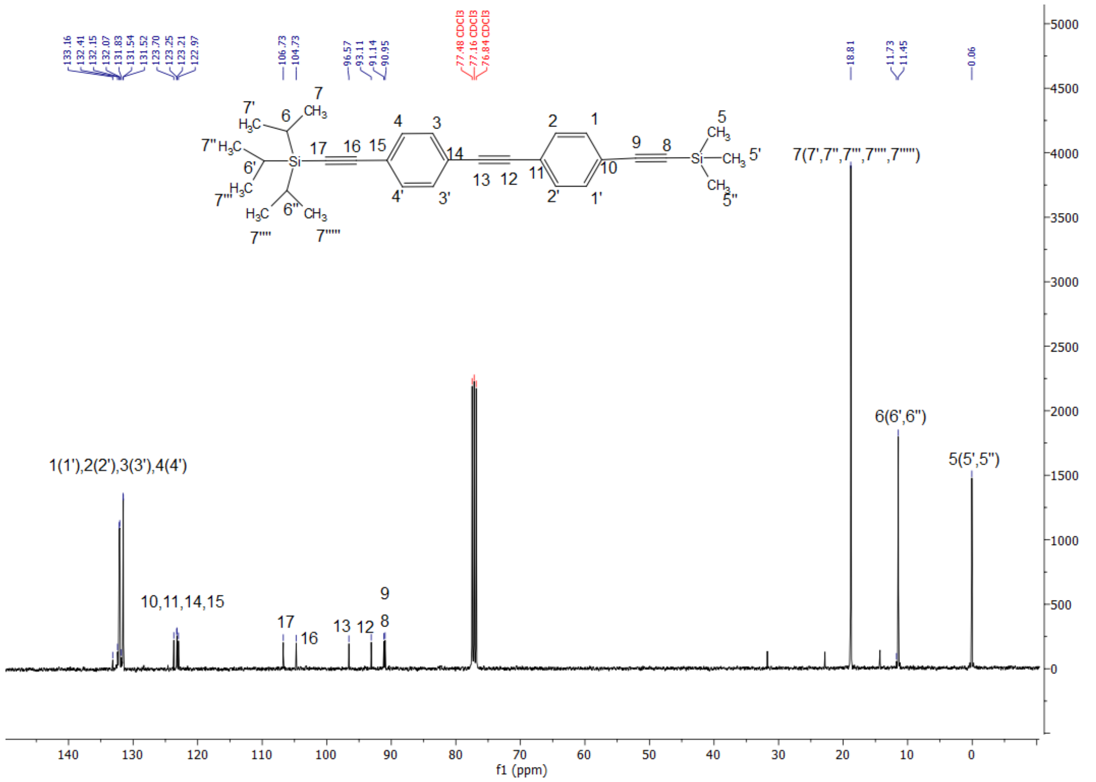

Figure N6: ^{13}CNMR for compound 3 in $CDCl_3$, 400 MHz.

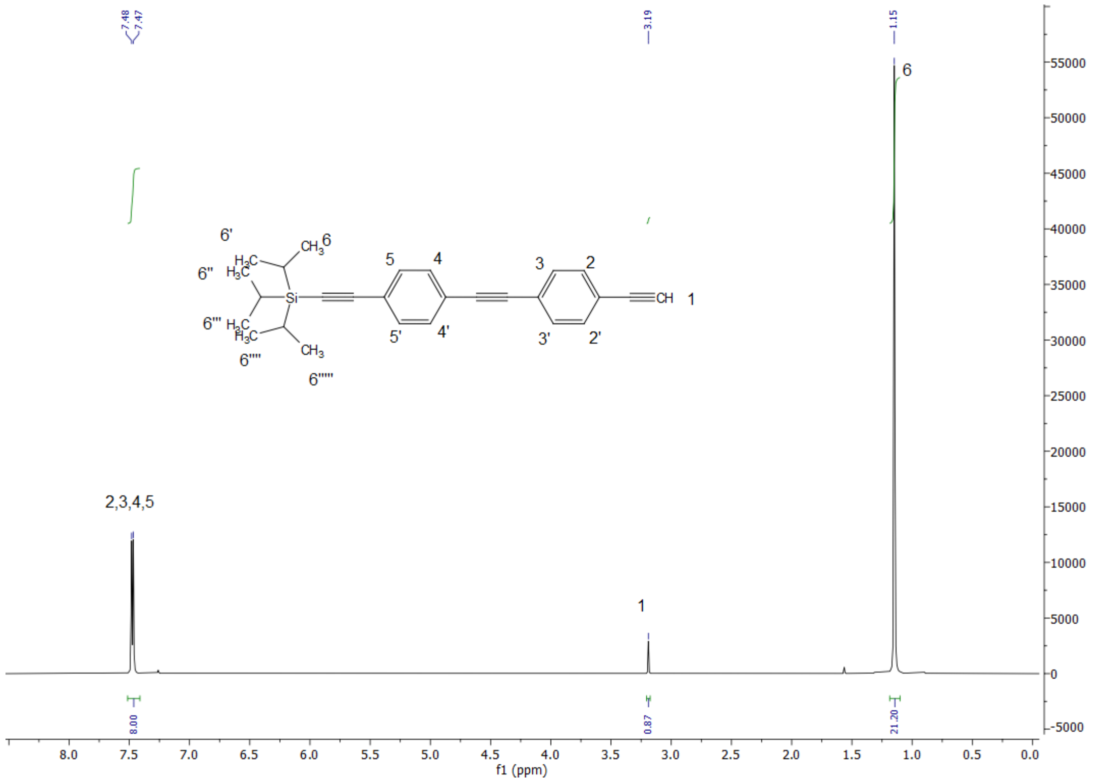

Figure N7: ^{1}H NMR for compound 4 in $CDCl_3$, 400 MHz.

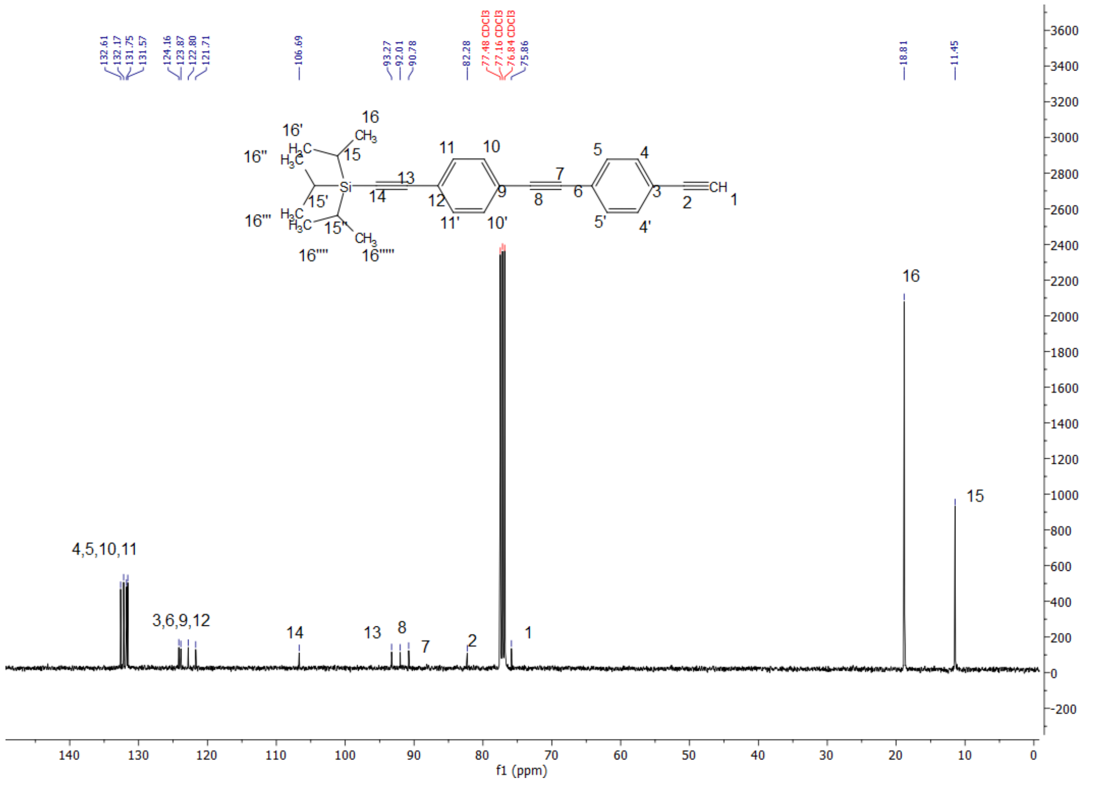

Figure N8: ^{13}C NMR for compound 4 in $CDCl_3$, 400 MHz.

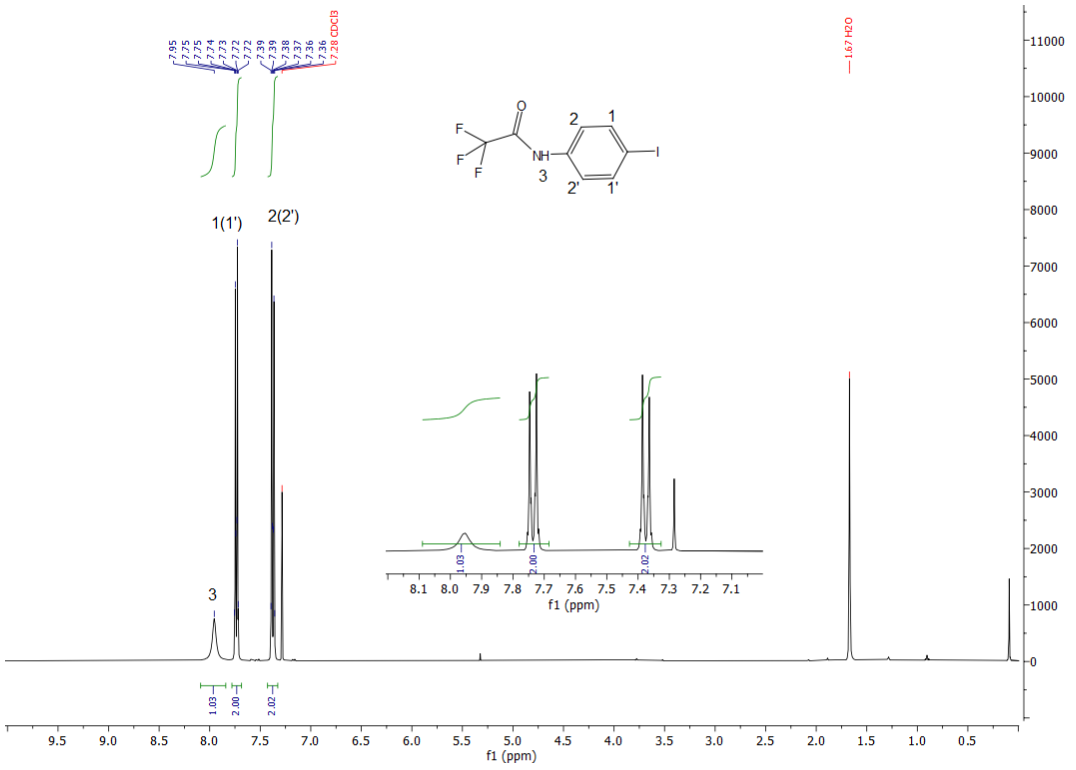

Figure N9: ^{1}H NMR for compound 5 in $CDCl_3$, 400 MHz.

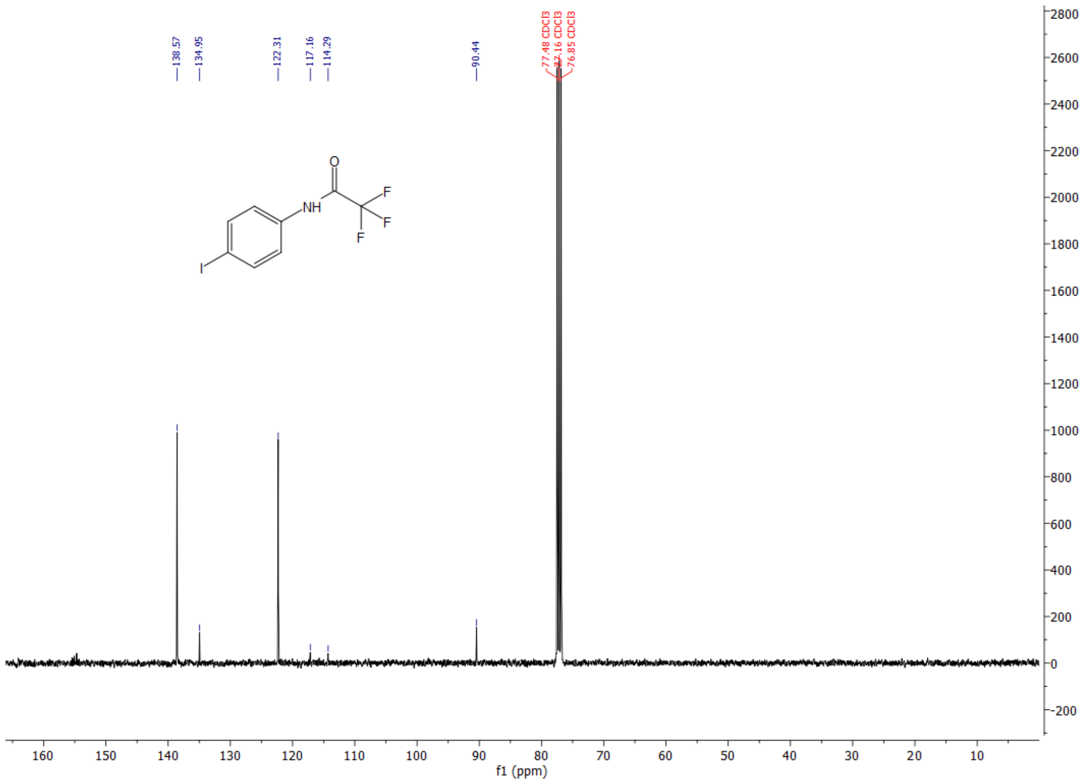

Figure N10: ^{13}C NMR for compound 5 in $CDCl_3$, 400 MHz.

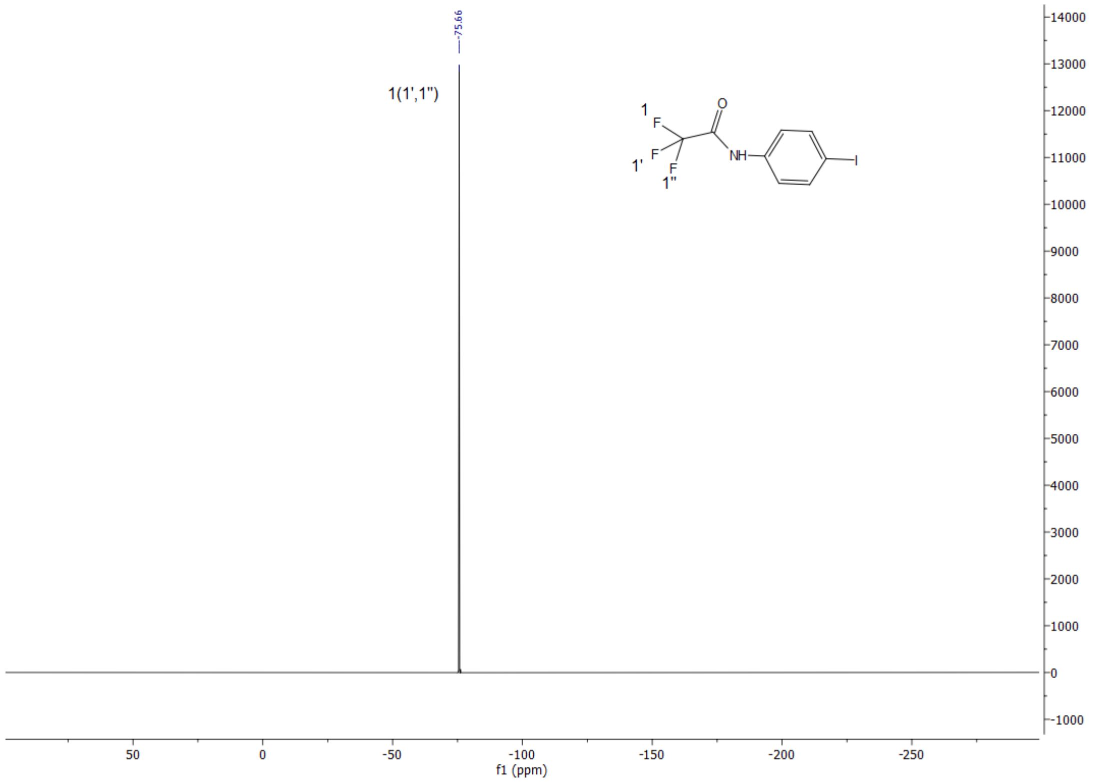

Figure N11: ^{19}F NMR for compound 5 in $CDCl_3$, 400 MHz.

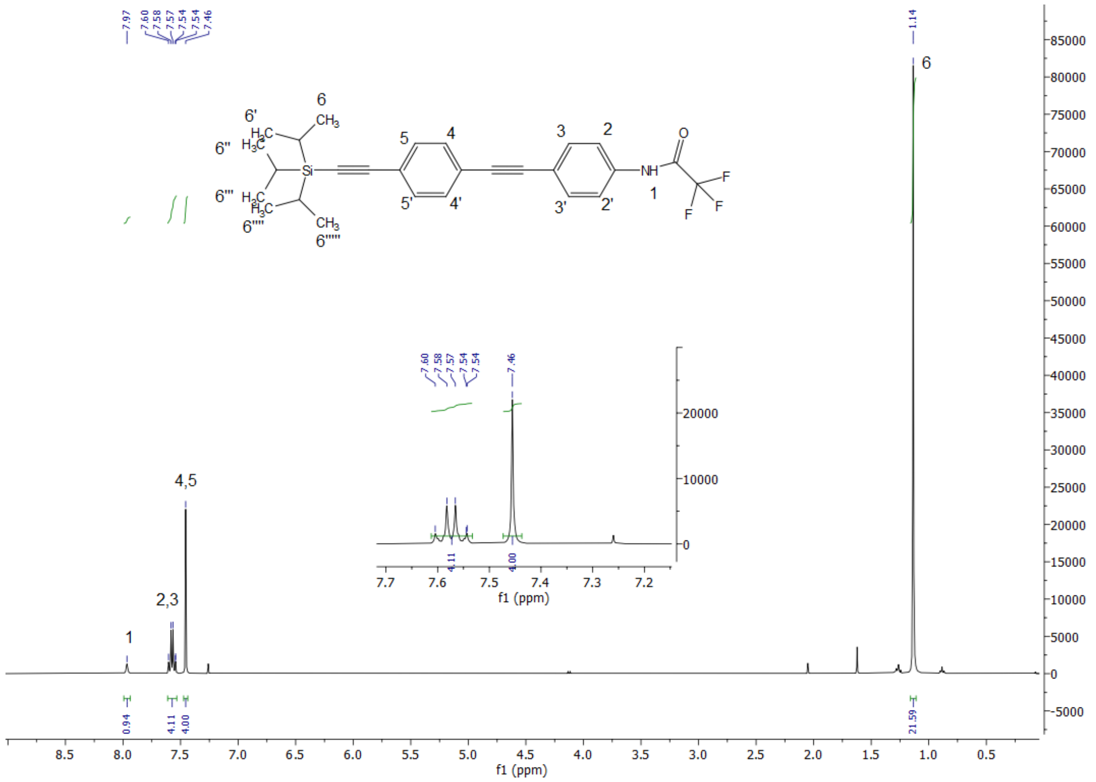

Figure N12: ^{1}H NMR for compound 6 in $CDCl_3$, 400 MHz.

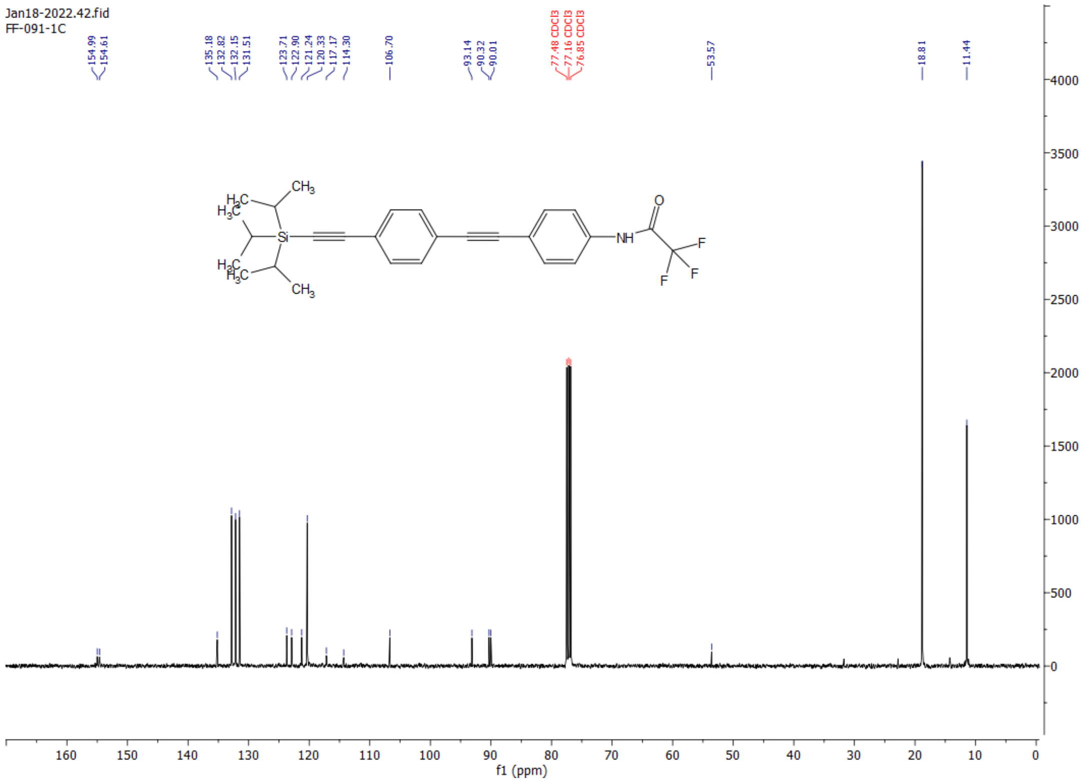

Figure N13: ^{13}C NMR for compound 6 in $CDCl_3$, 400 MHz.

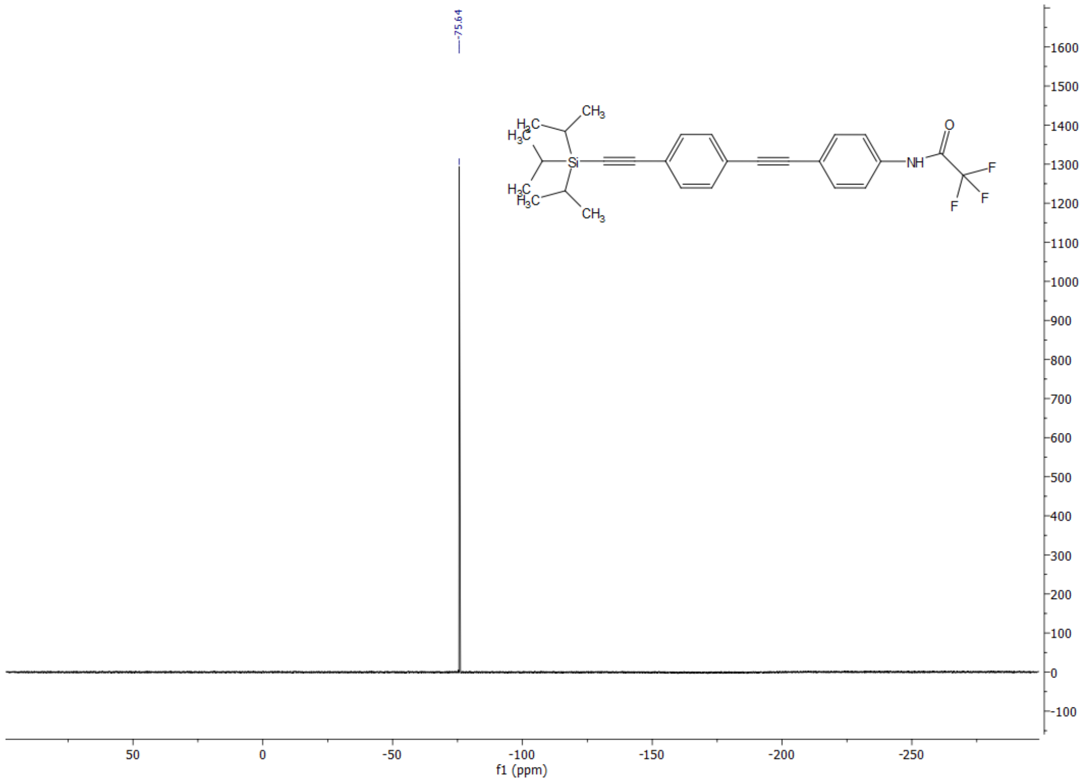

Figure N14: ^{19}F NMR for compound 6 in $CDCl_3$, 400 MHz.

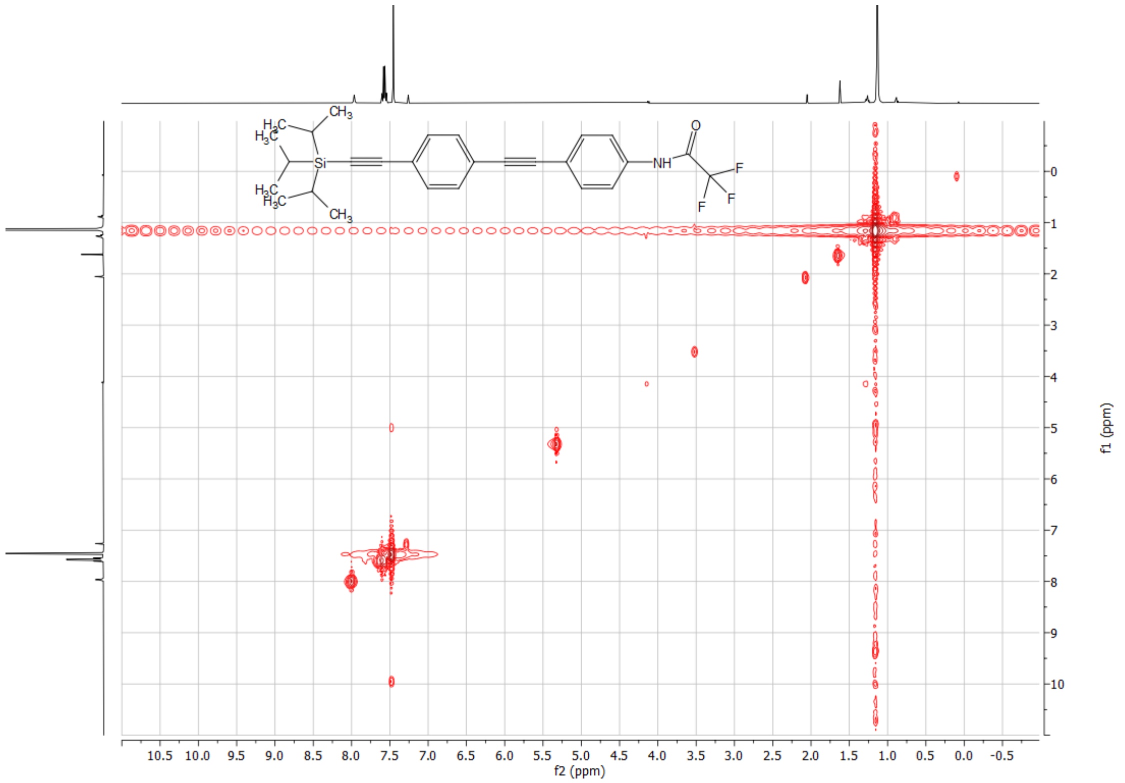

Figure N15: COSY NMR H-H correlation for compound 6 in $CDCl_3$, 400 MHz

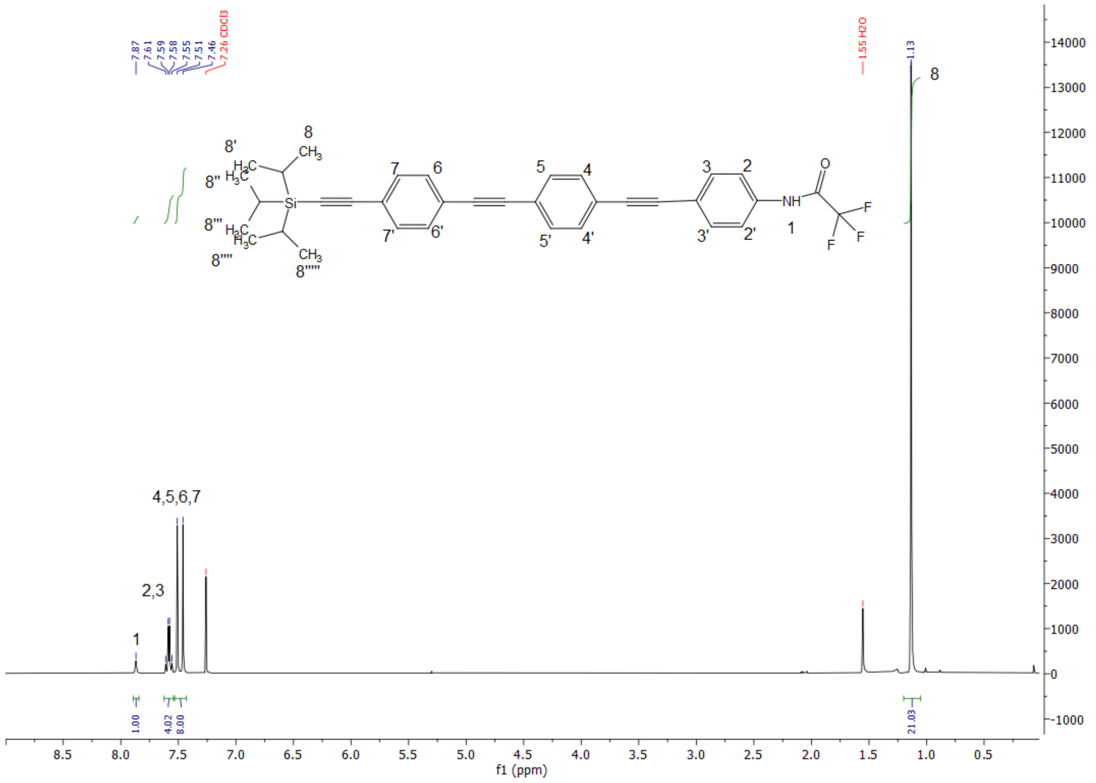

Figure N16: ^1H NMR for compound 7 in $CDCl_3$, 400 MHz

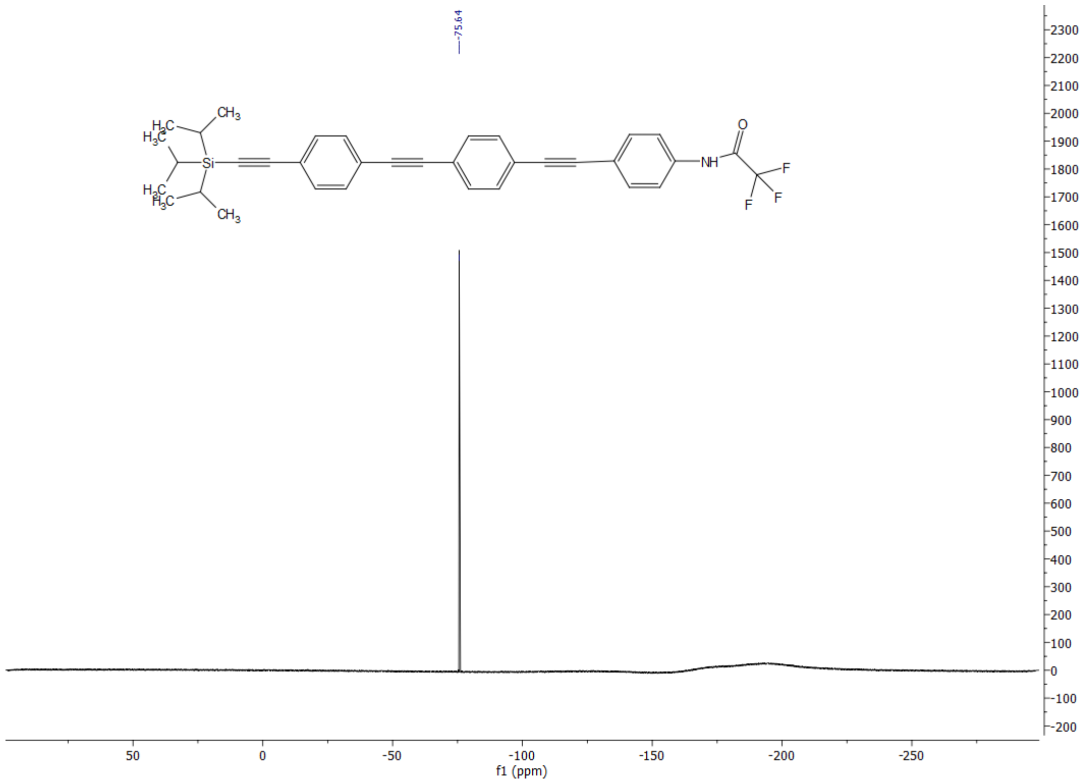

Figure N17: ^{19}F NMR for compound 7 in $CDCl_3$, 400 MHz

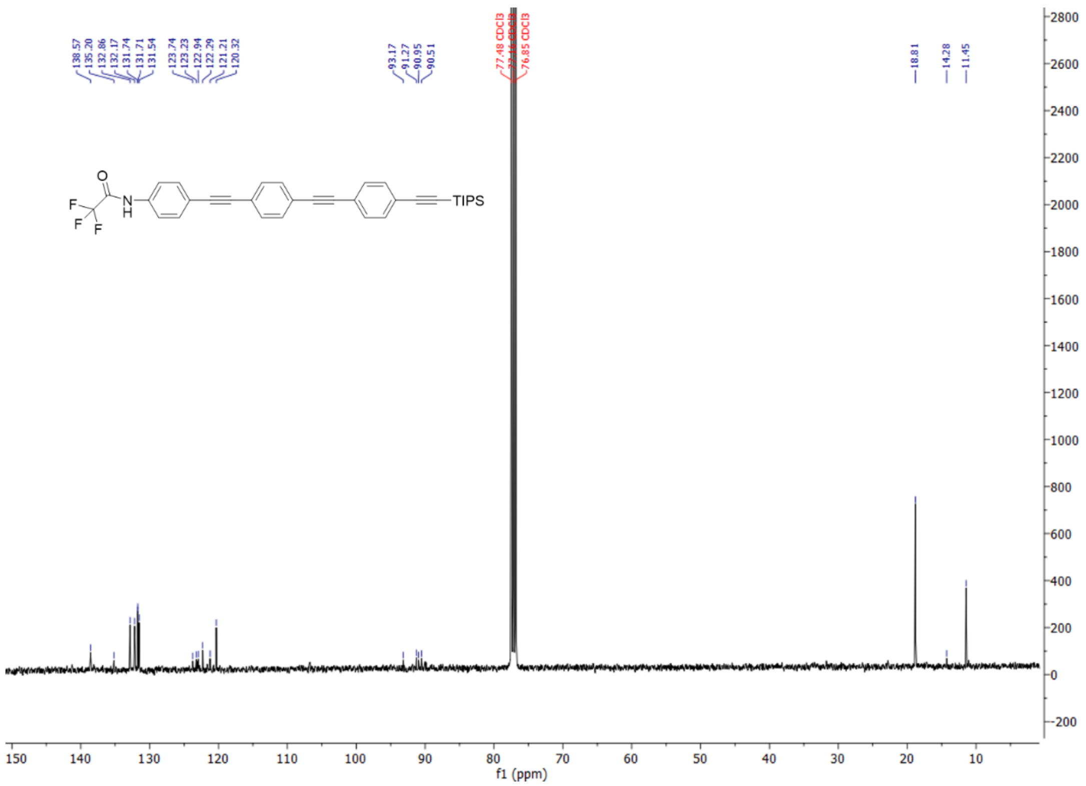

Figure N18: ^{13}C for compound 7 in $CDCl_3$, 400 MHz

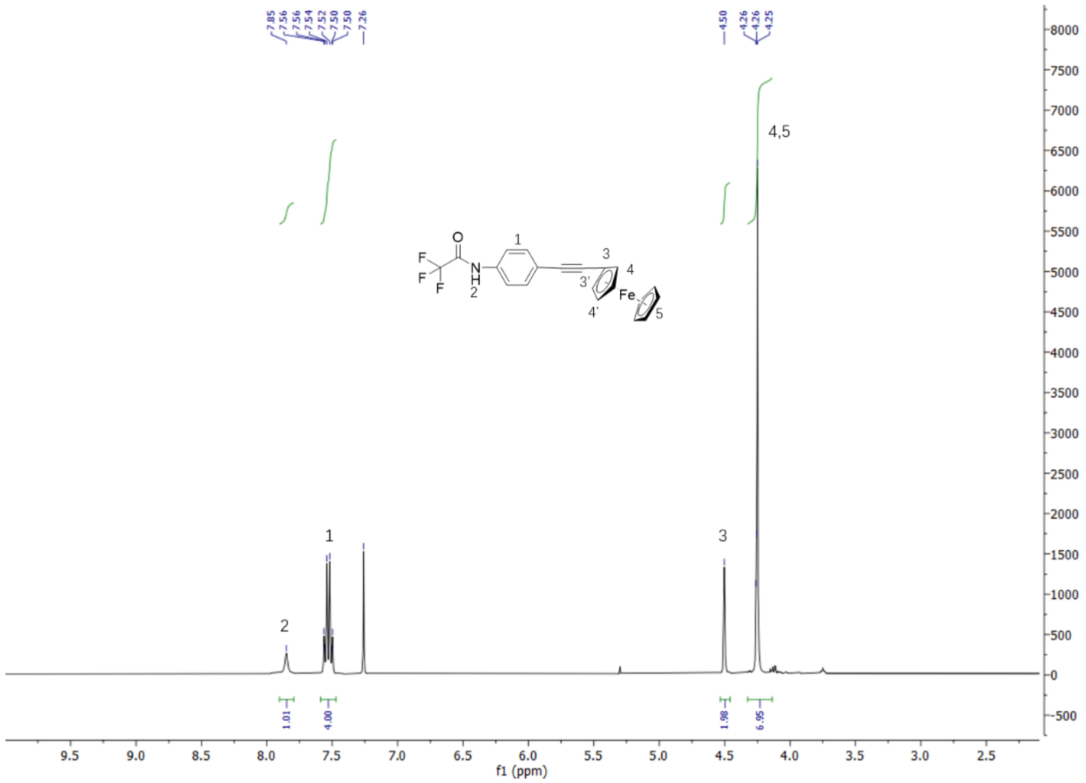

Figure N19: ^{1}H NMR for compound 8 in $CDCl_3$, 400 MHz

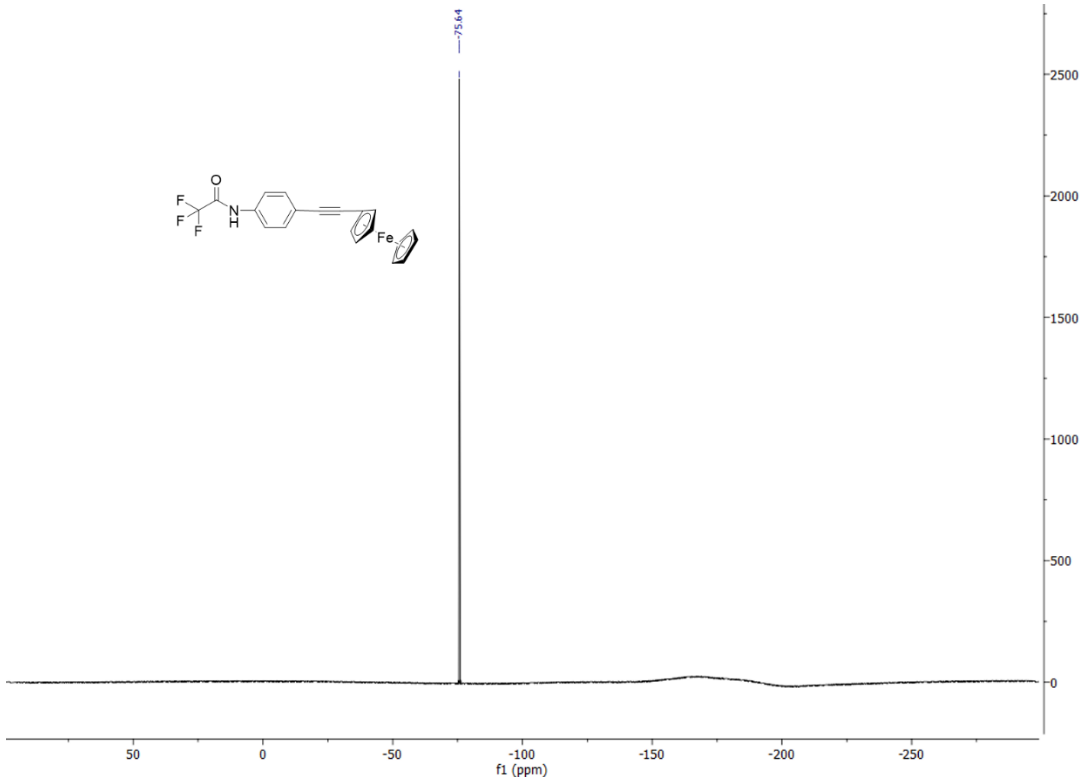

Figure N20: ^{19}F NMR for compound 8 in $CDCl_3$, 400 MHz

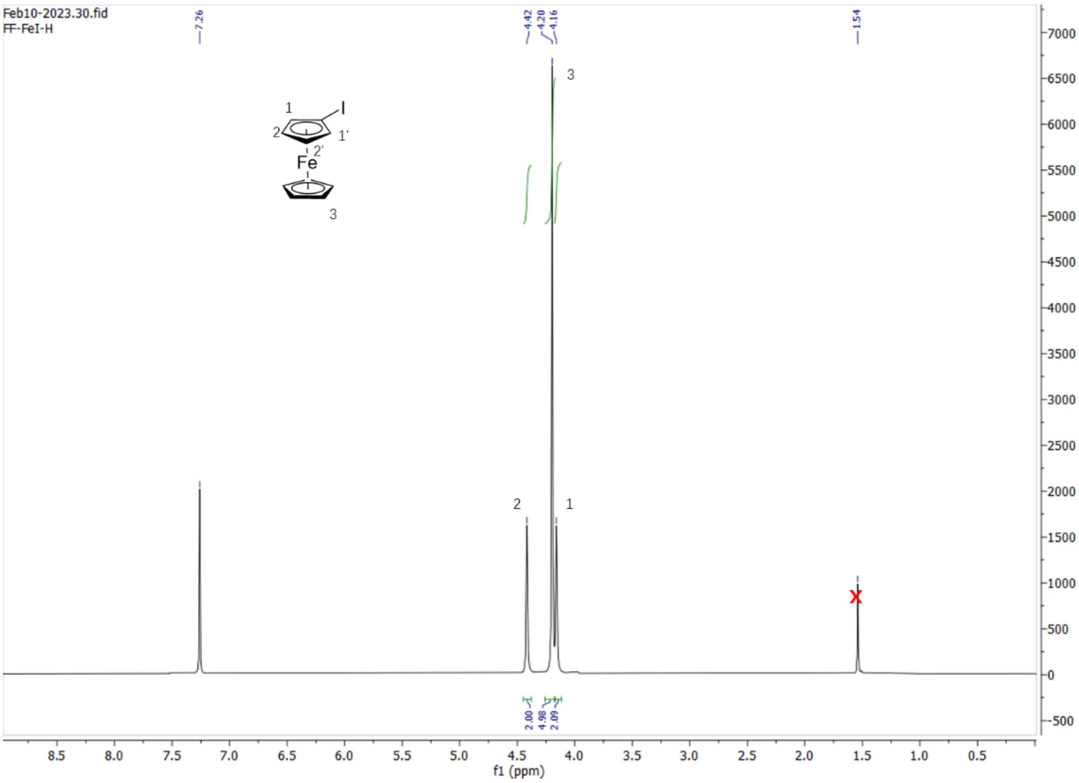

Figure N21: ^{1}H NMR for compound 9 in $CDCl_3$, 400 MHz

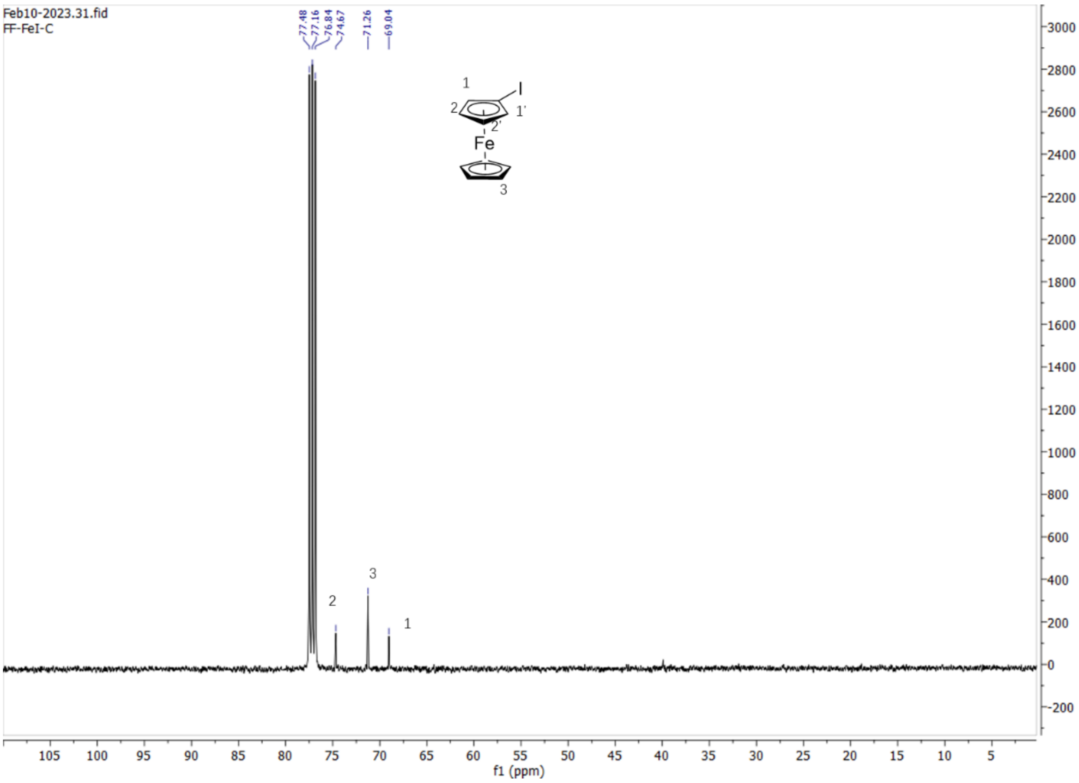

Figure N22: ^{13}C NMR for compound 9 in $CDCl_3$, 400 MHz

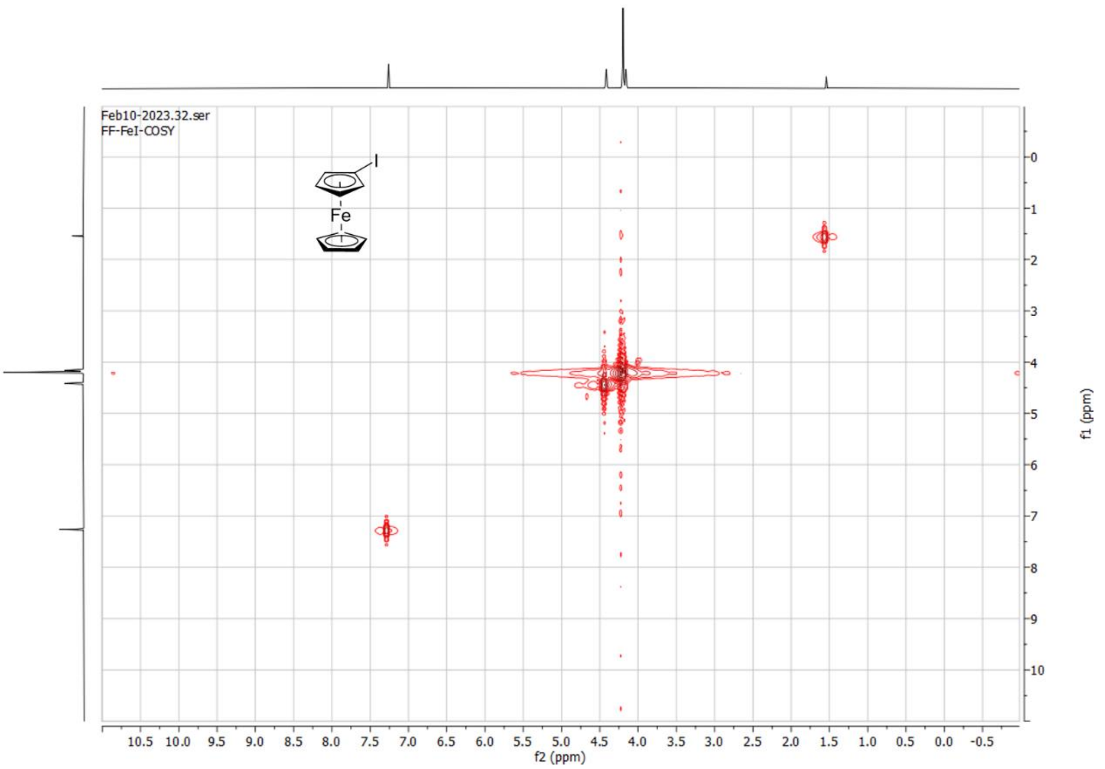

Figure N23: COSY NMR spectrum for compound 9 in $CDCl_3$, 400 MHz

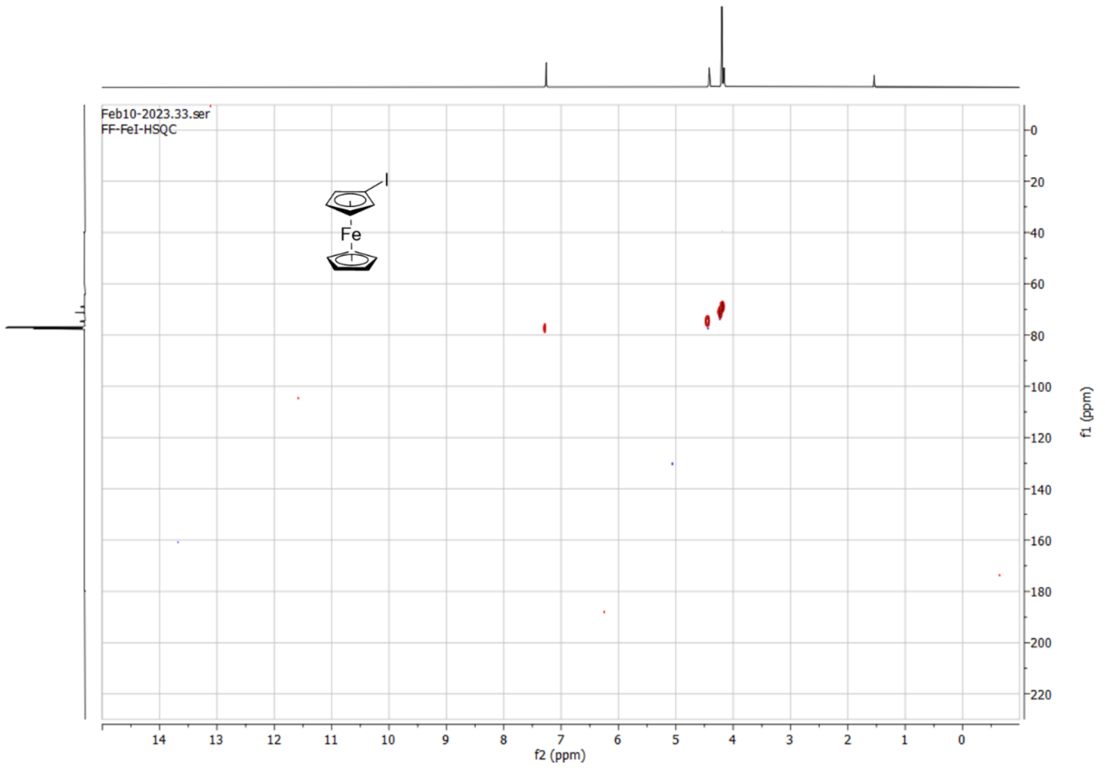

Figure N24: HSQC NMR spectrum for compound 9 in $CDCl_3$, 400 MHz

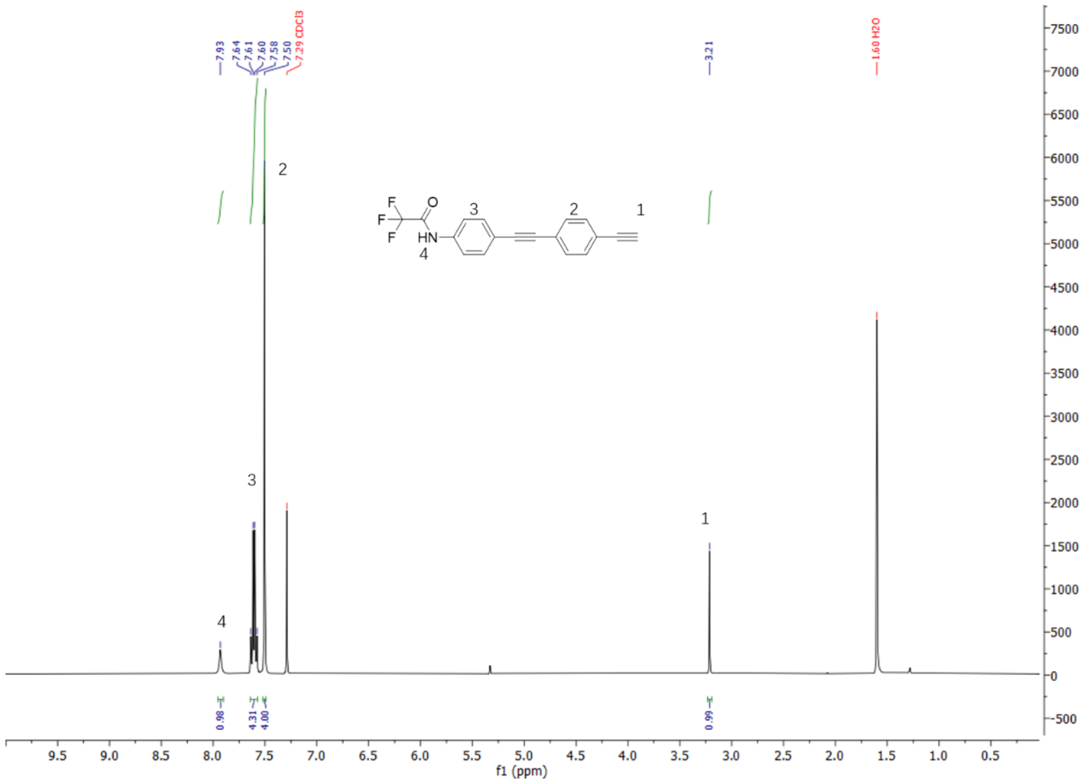

Figure N25: ^{1}H NMR spectrum for compound 10 in $CDCl_3$, 400 MHz

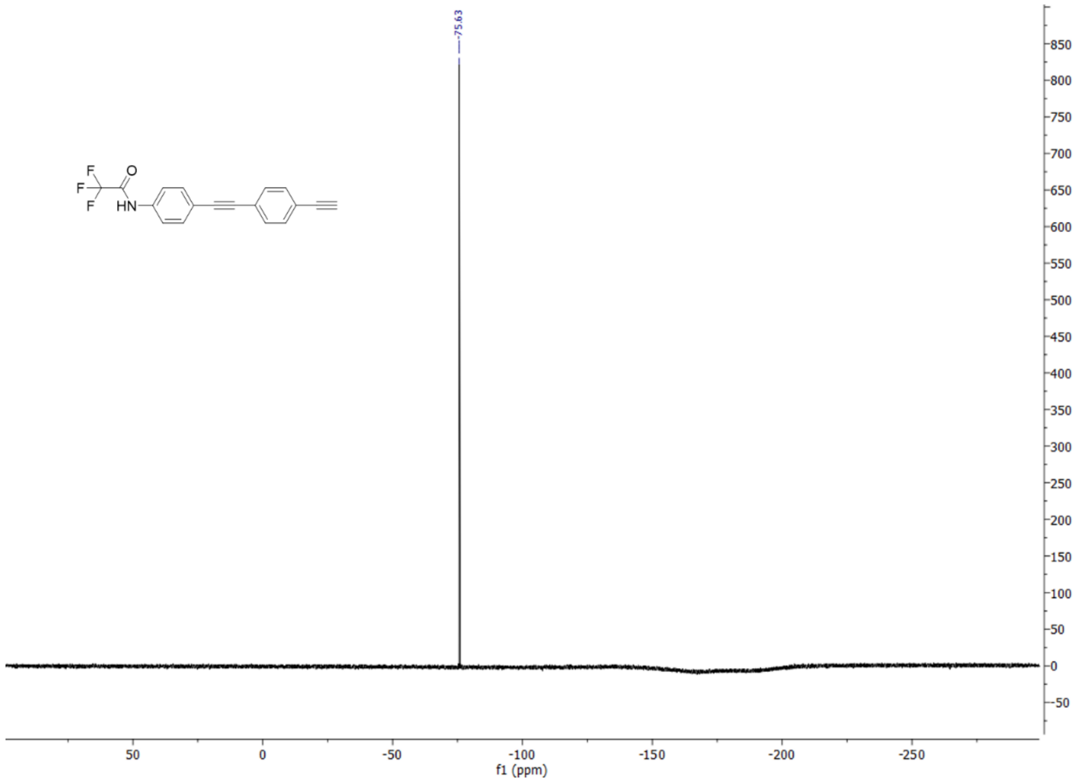

Figure N26: ^{19}F NMR spectrum for compound 10 in $CDCl_3$, 400 MHz

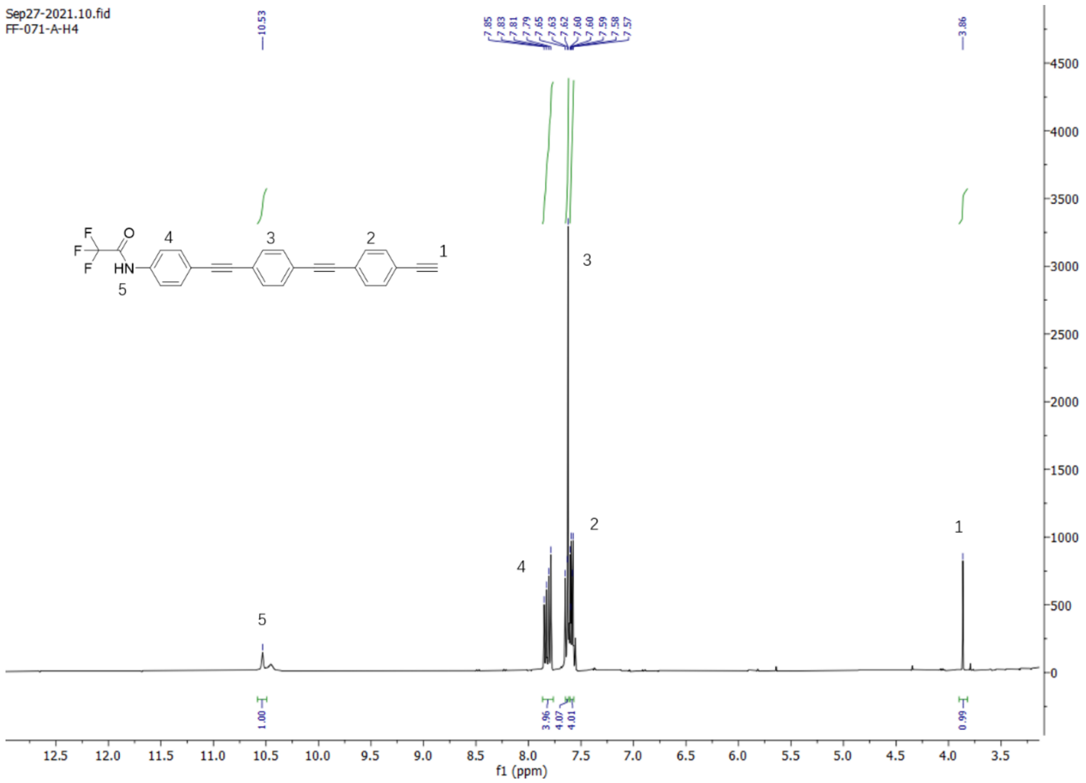

Figure N27: ^{1}H NMR spectrum for compound 11 in $CDCl_3$, 400 MHz

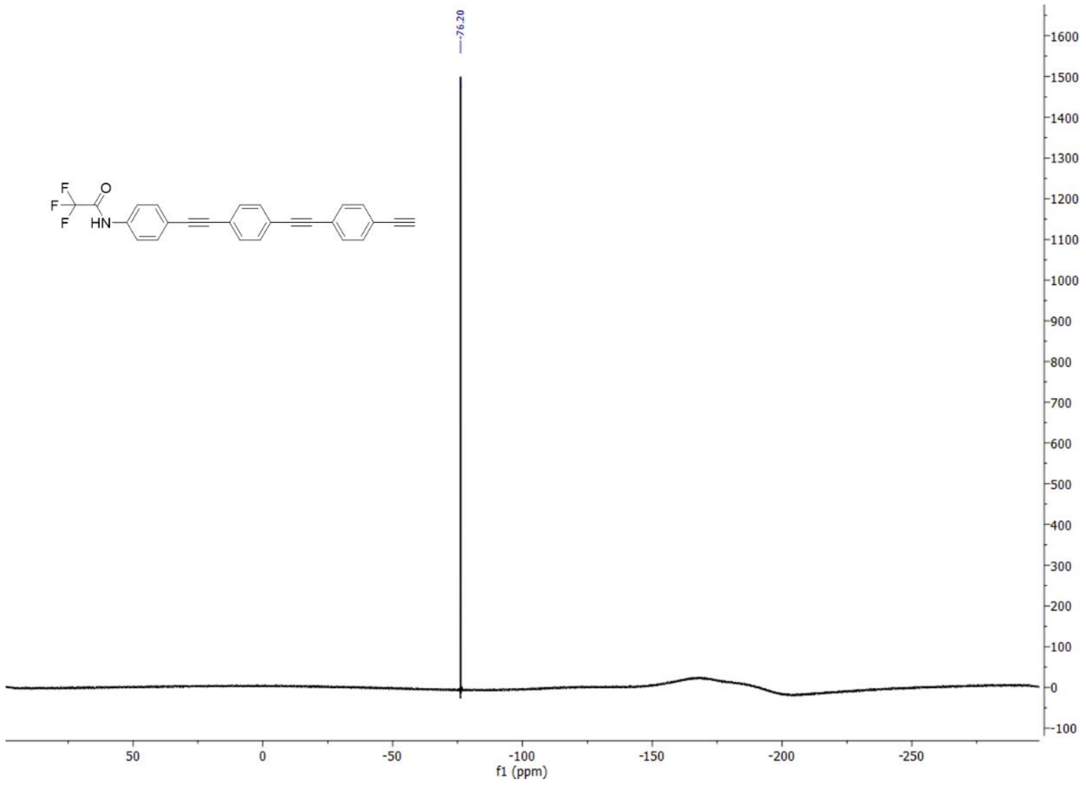

Figure N28: ^{19}F NMR spectrum for compound 11 in $CDCl_3$, 400 MHz

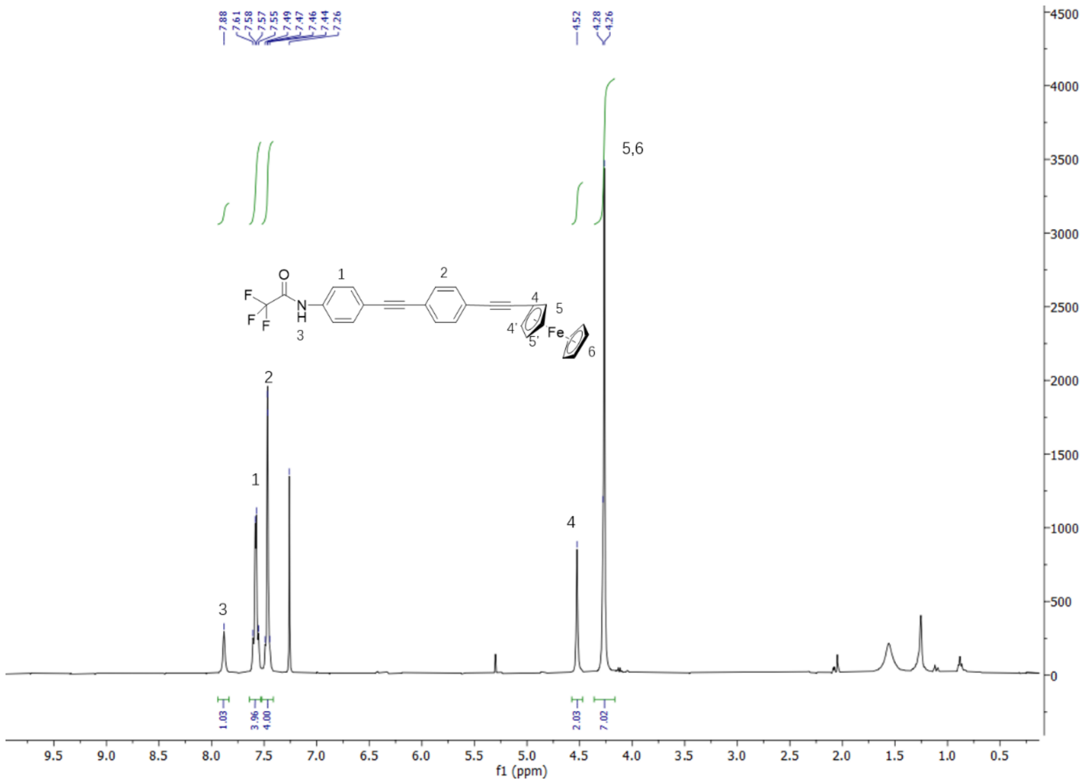

Figure N29: ^{1}H NMR spectrum for compound 12 in $CDCl_3$, 400 MHz

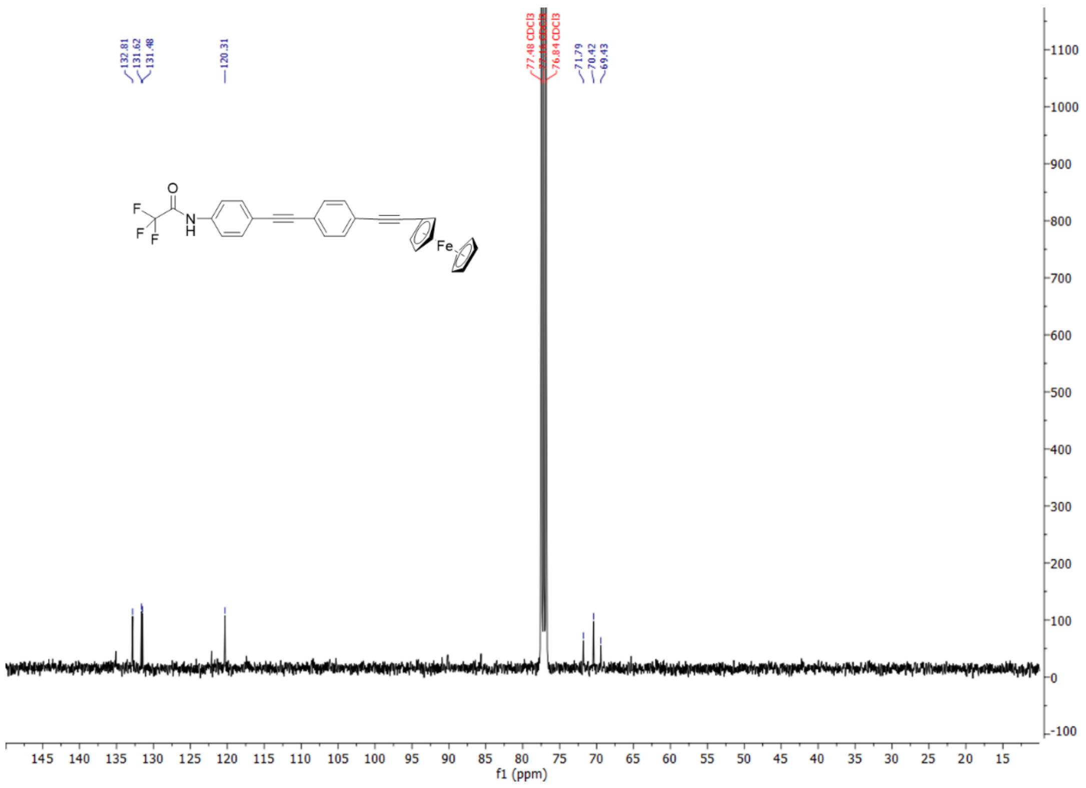

Figure N30: ^{13}C NMR for compound 12 in $CDCl_3$, 400 MHz

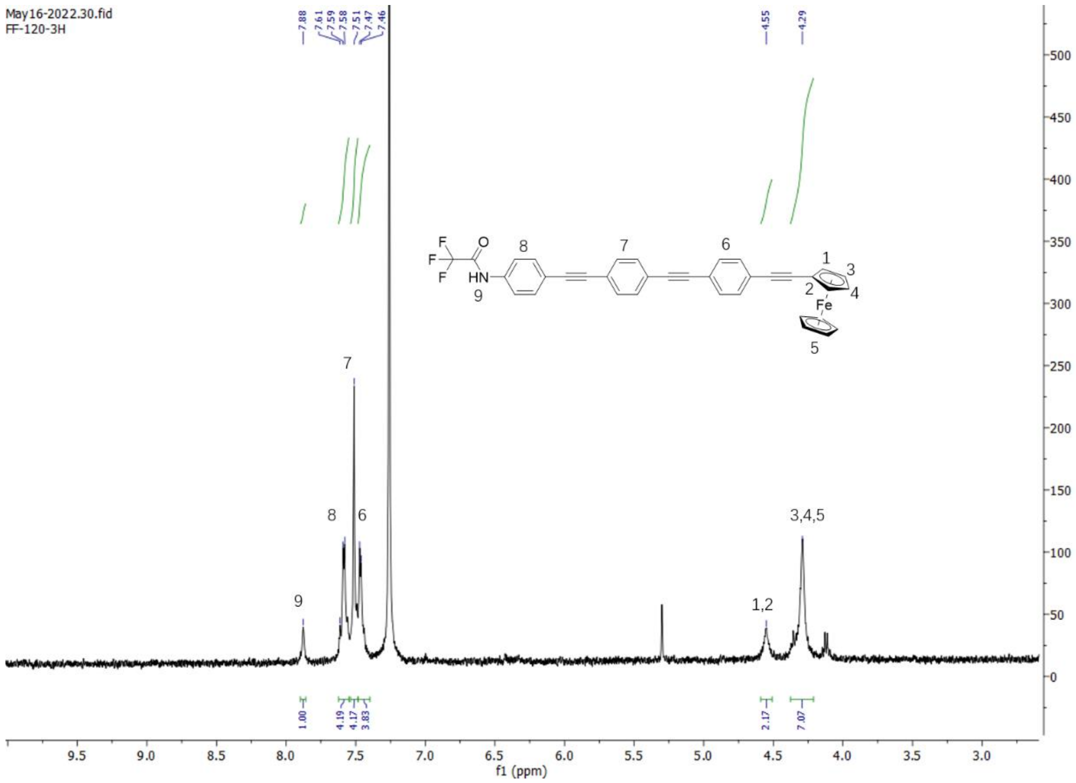

Figure N31: ^{1}H NMR spectrum for compound 13 in $CDCl_3$, 400 MHz

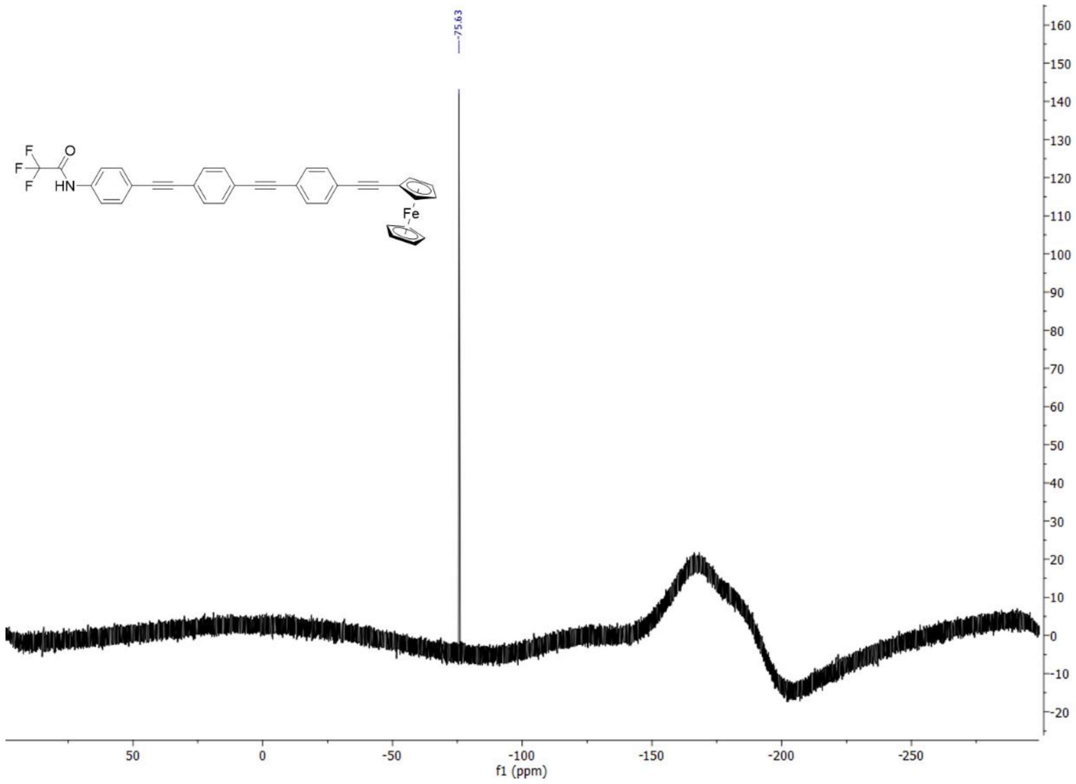

Figure N32: ^{19}F NMR spectrum for compound 13 in $CDCl_3$, 400 MHz

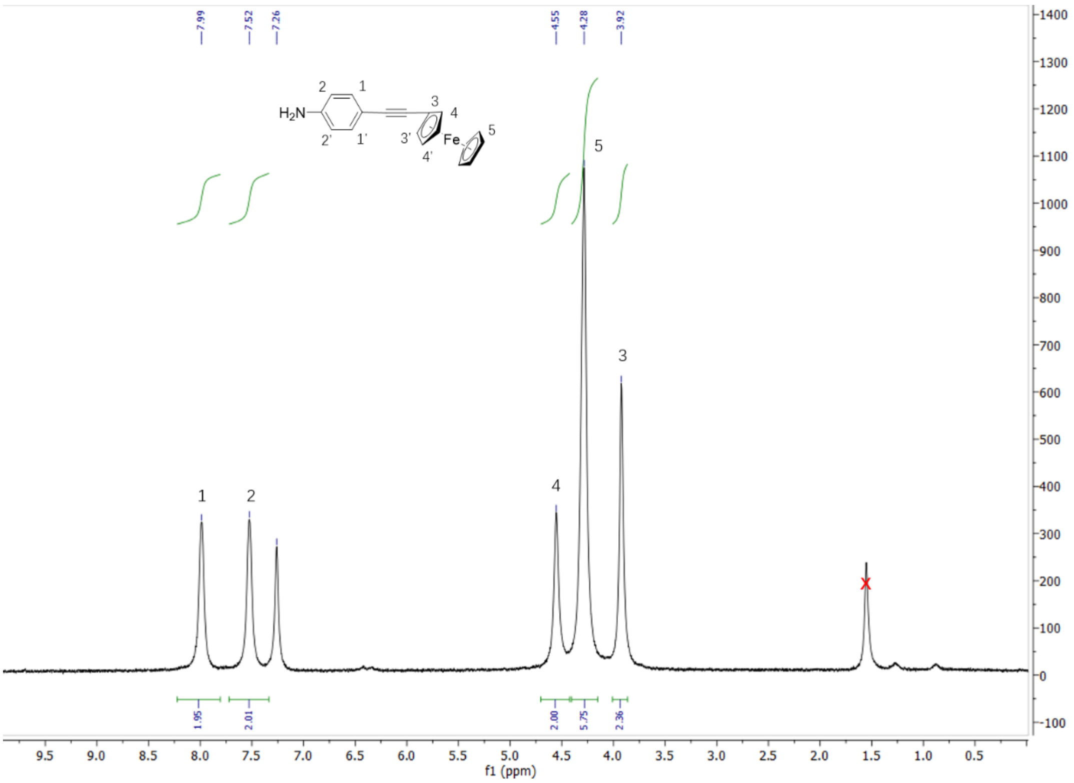

Figure N33: ^{1}H NMR spectrum for Compound I in $CDCl_3$, 400 MHz

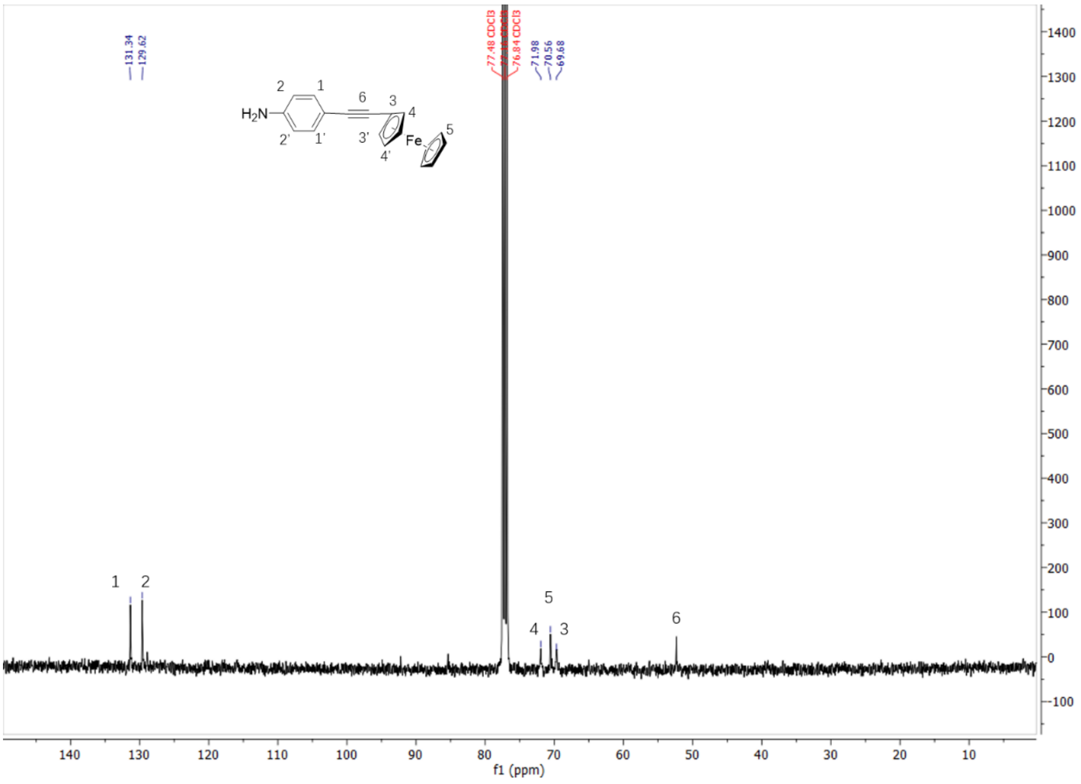

Figure N34: ^{13}C NMR spectrum for Compound I in $CDCl_3$, 400 MHz

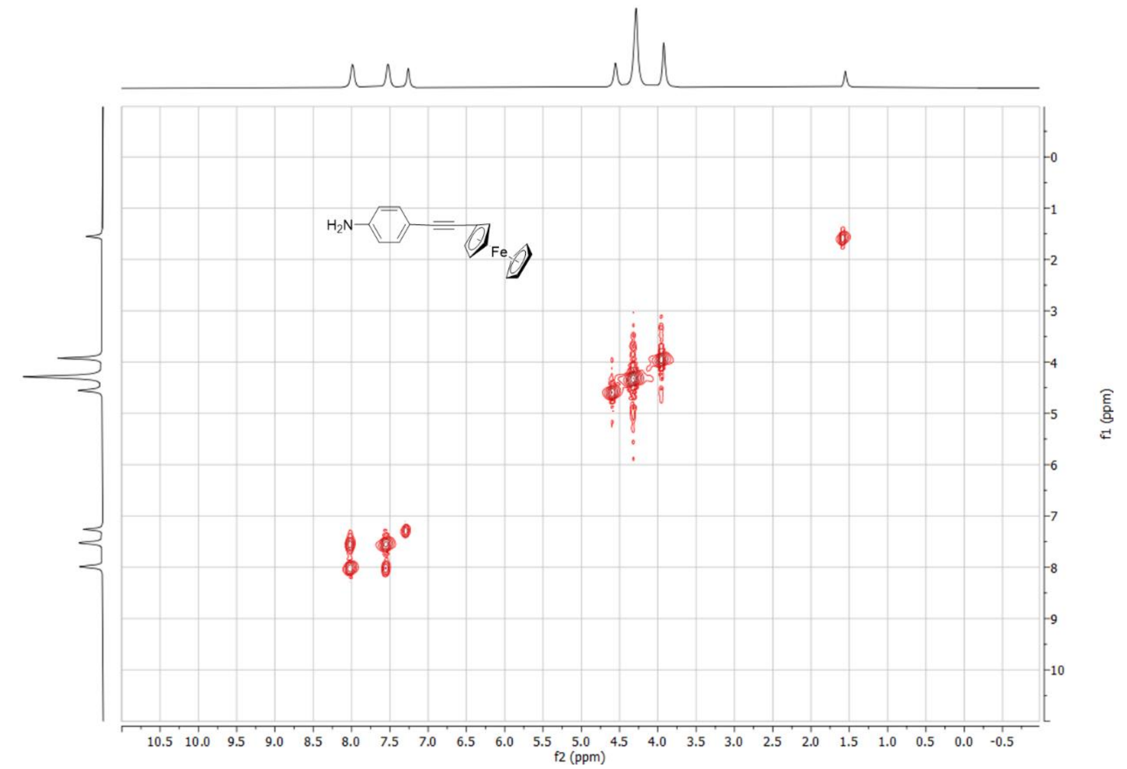

Figure N35: COSY NMR spectrum for Compound I in $CDCl_3$, 400 MHz

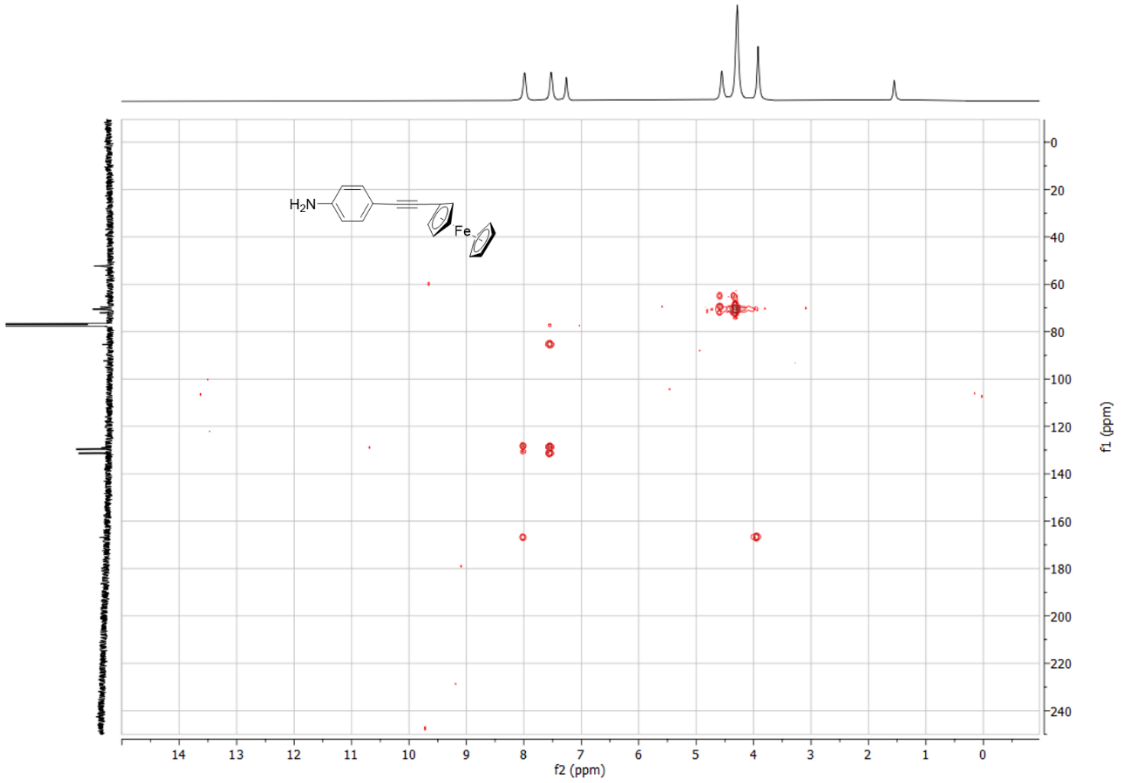

Figure N36: HMBC NMR spectrum for Compound I in $CDCl_3$, 400 MHz

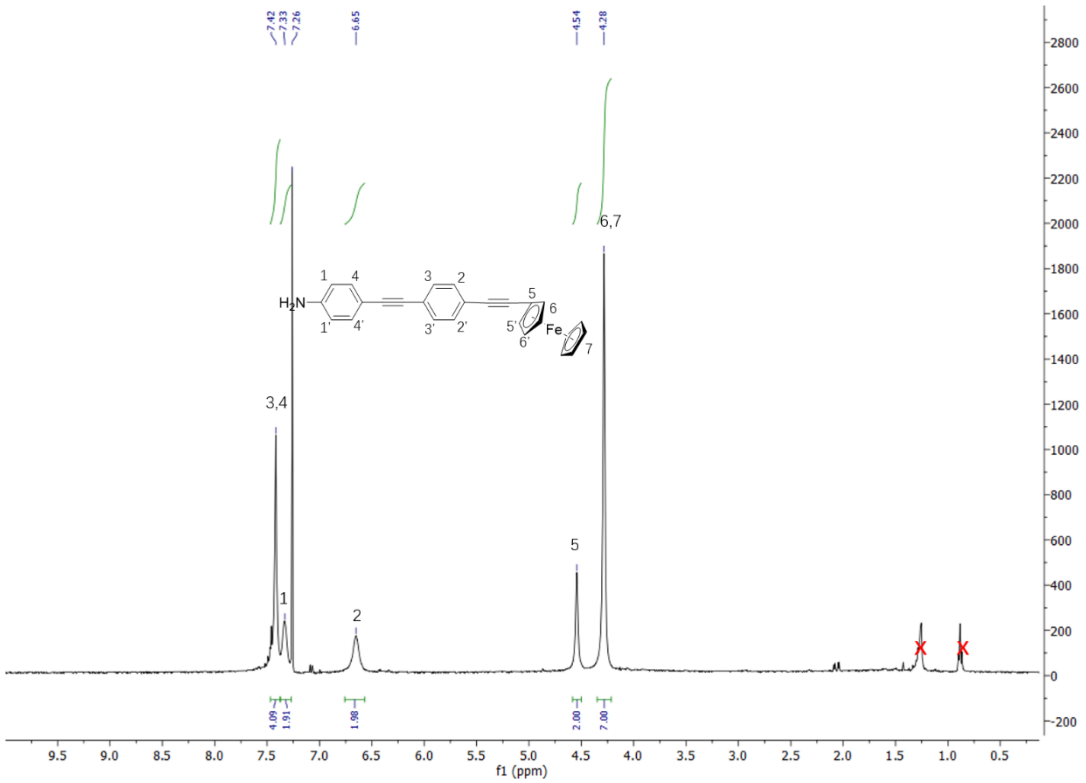

Figure N37: ^{1}H NMR for Compound II in $CDCl_3$, 400 MHz

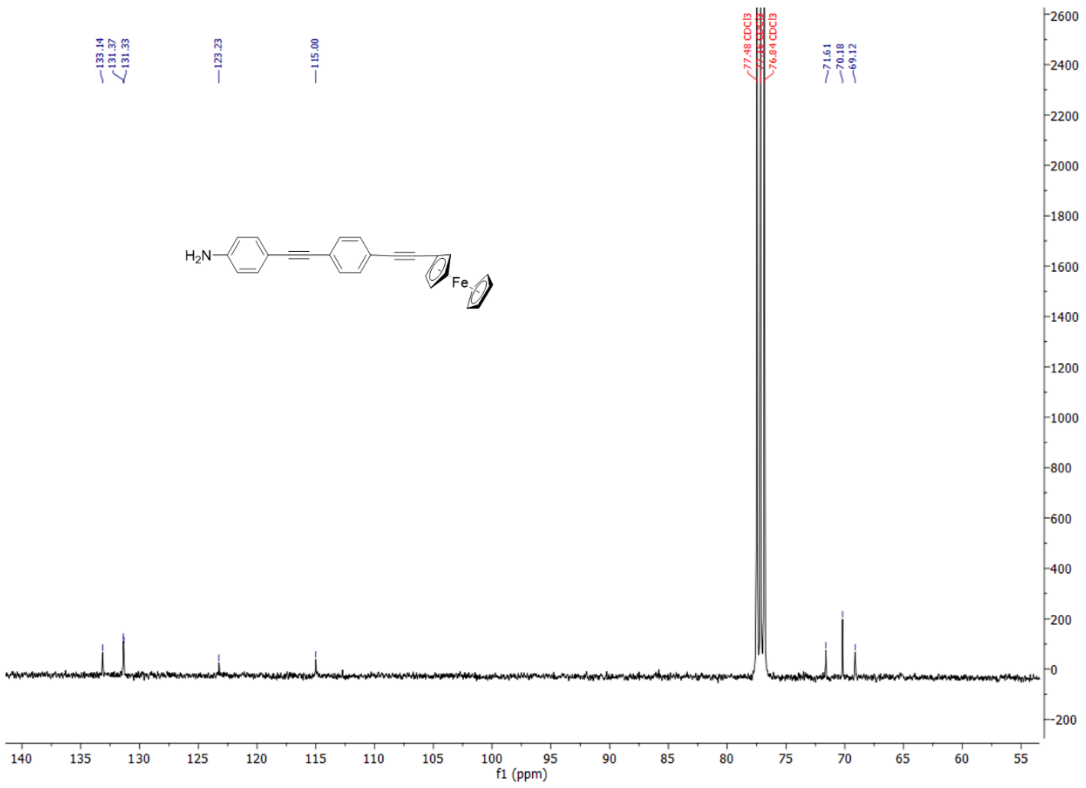

Figure N38: ^{13}C NMR for Compound II in $CDCl_3$, 400 MHz

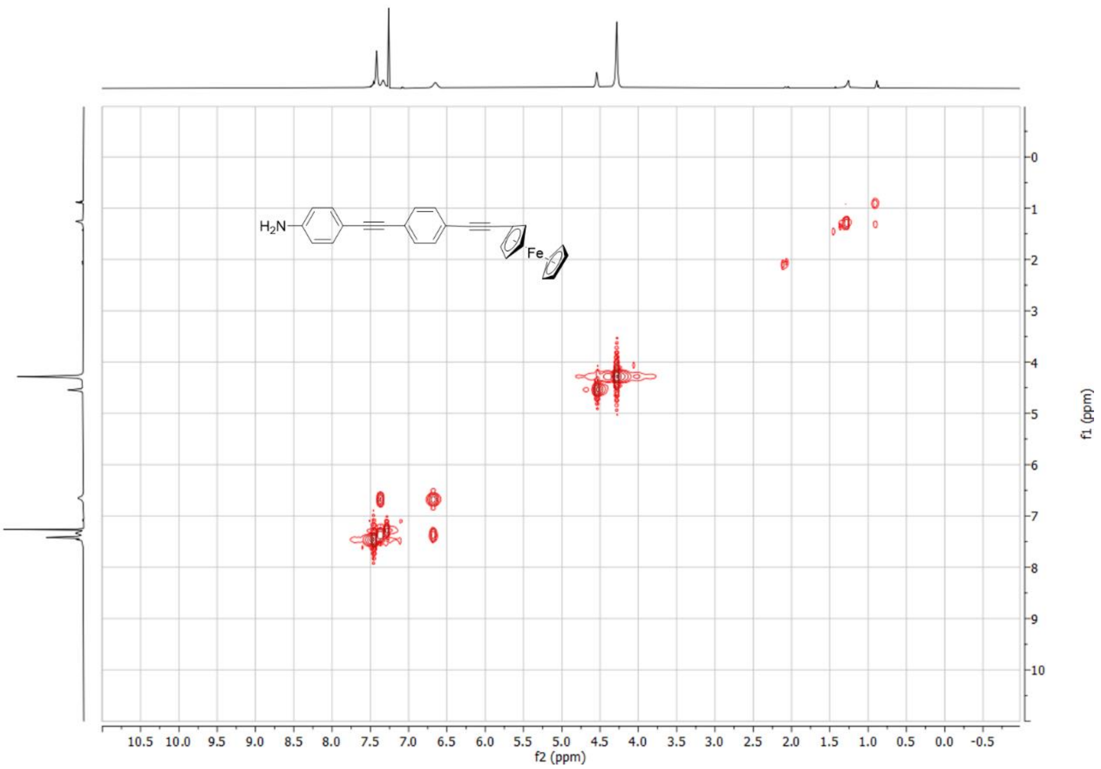

Figure N39: COSY NMR spectrum for Compound II in $CDCl_3$, 400 MHz

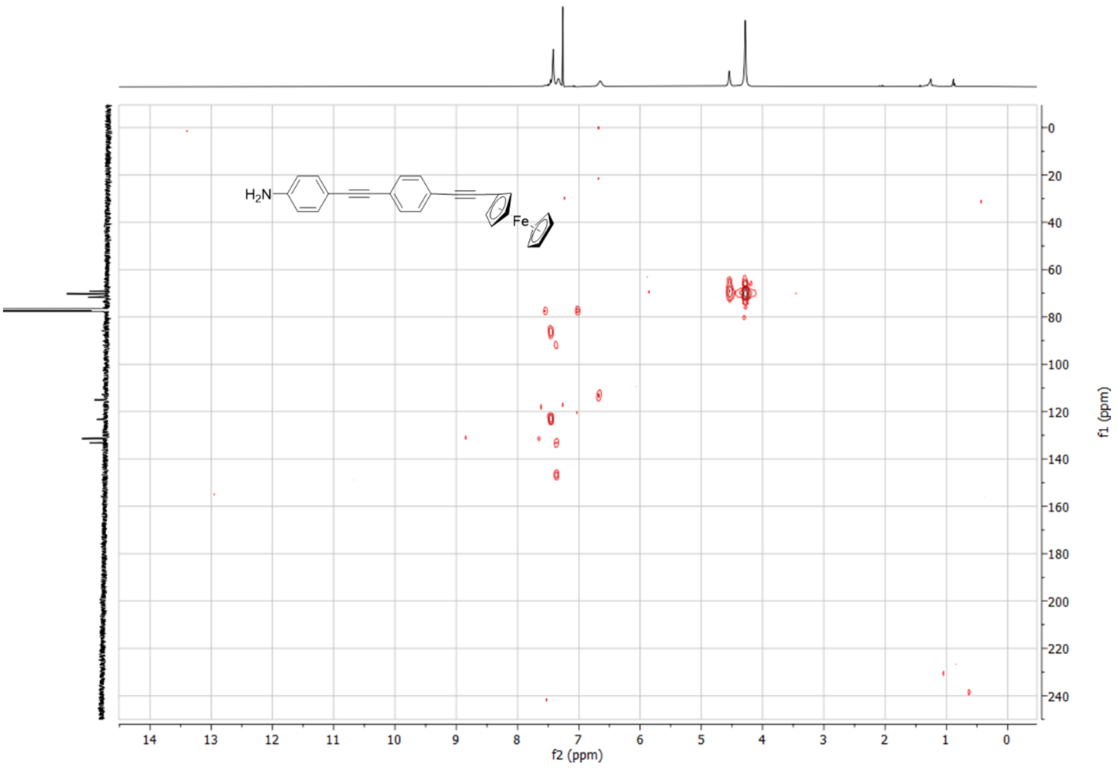

Figure N40: HMBC NMR spectrum for Compound II in $CDCl_3$, 400 MHz

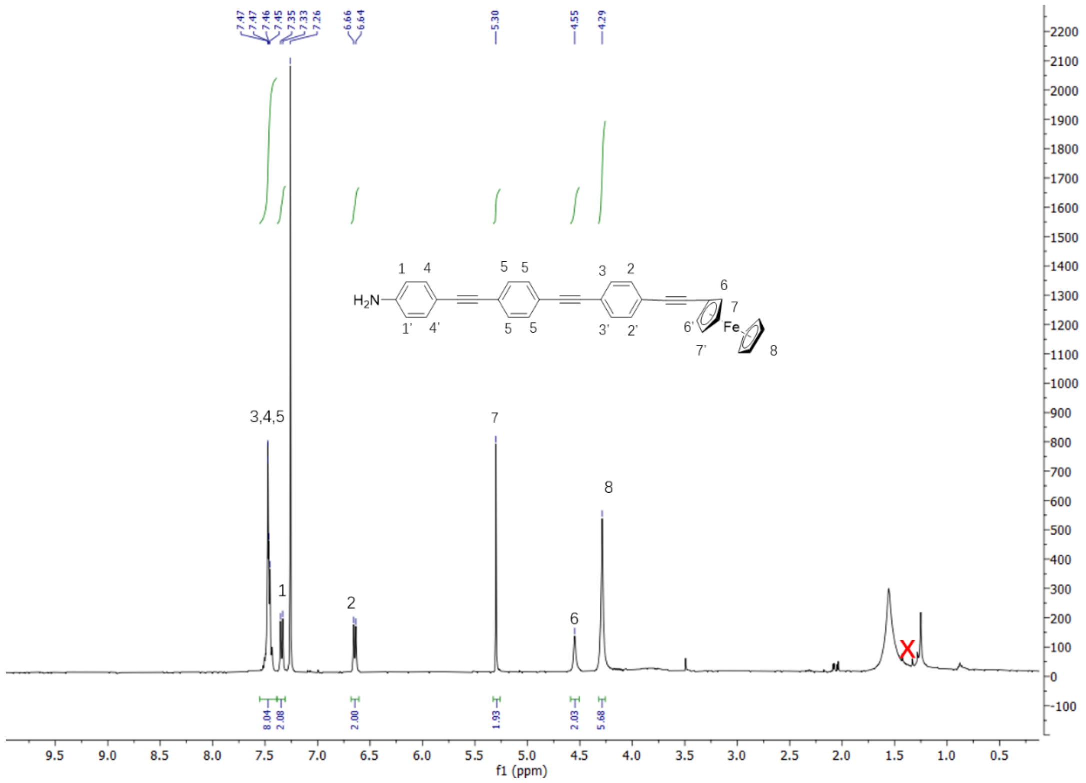

Figure N41: ^{1}H NMR for Compound III in $CDCl_3$, 400 MHz

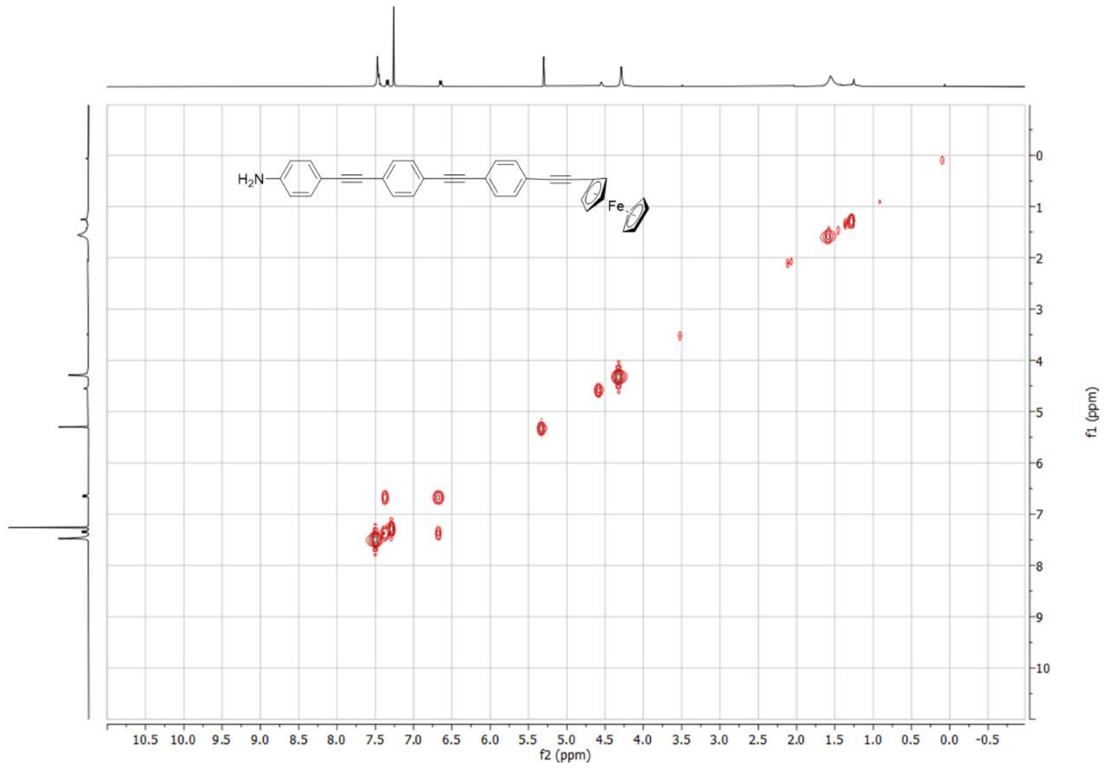

Figure N42: COSY NMR spectrum for Compound III in $CDCl_3$, 400 MHz

3 UV-vis data for the compound series

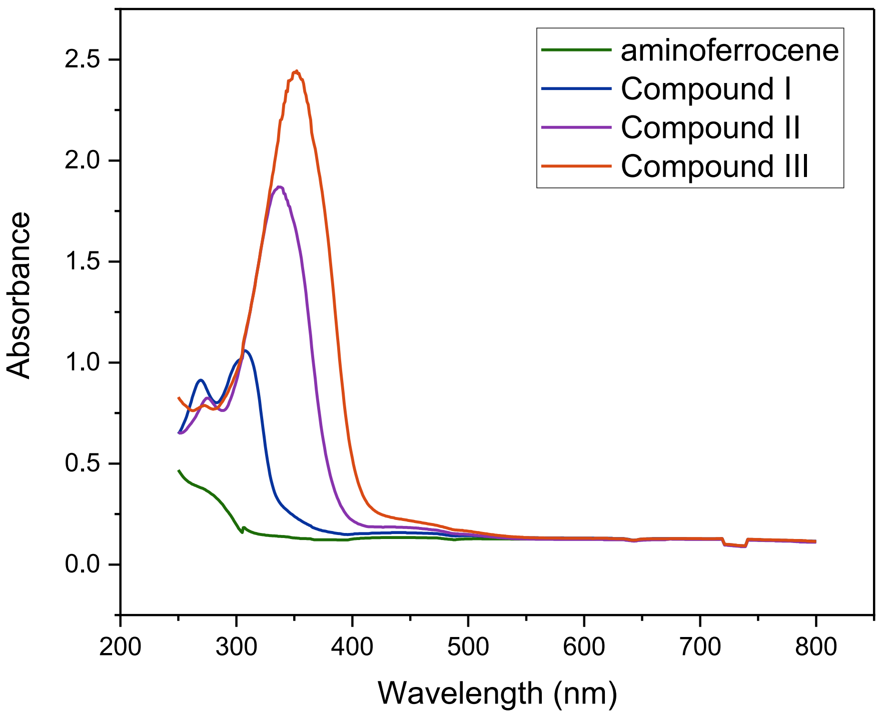

Figure 2: UV-vis spectrum for aminoferrocene and compounds I-III. The compounds were dissolved into DCM to prepare 1 mM solutions and measured in room temperature.

4 Electrochemistry for aminoferrocene and compounds I to III

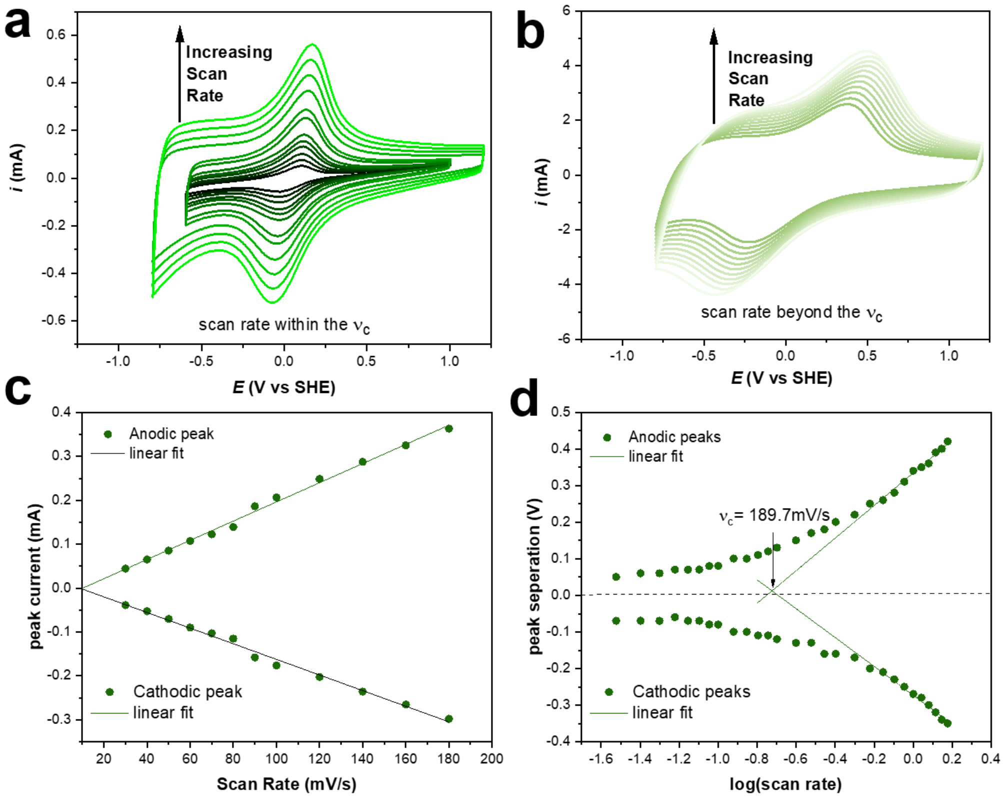

Figure 3: Electrochemical characterisation of aminoferrocene on IO-mesoITO. **a,** Cyclic voltammetry of aminoferrocene on IO-mesoITO surface within critical scan rate (30-180mv/s); **b,** Cyclic voltammetry of aminoferrocene on IO-mesoITO surface beyond critical scan rate (1000-2000 mv/s); **c,** Trumpet plot of aminoferrocene derived from scan rate 30-2000 mV/s; **d,** Randles-Sevcik plot of aminoferrocene on IO-mesoITO.

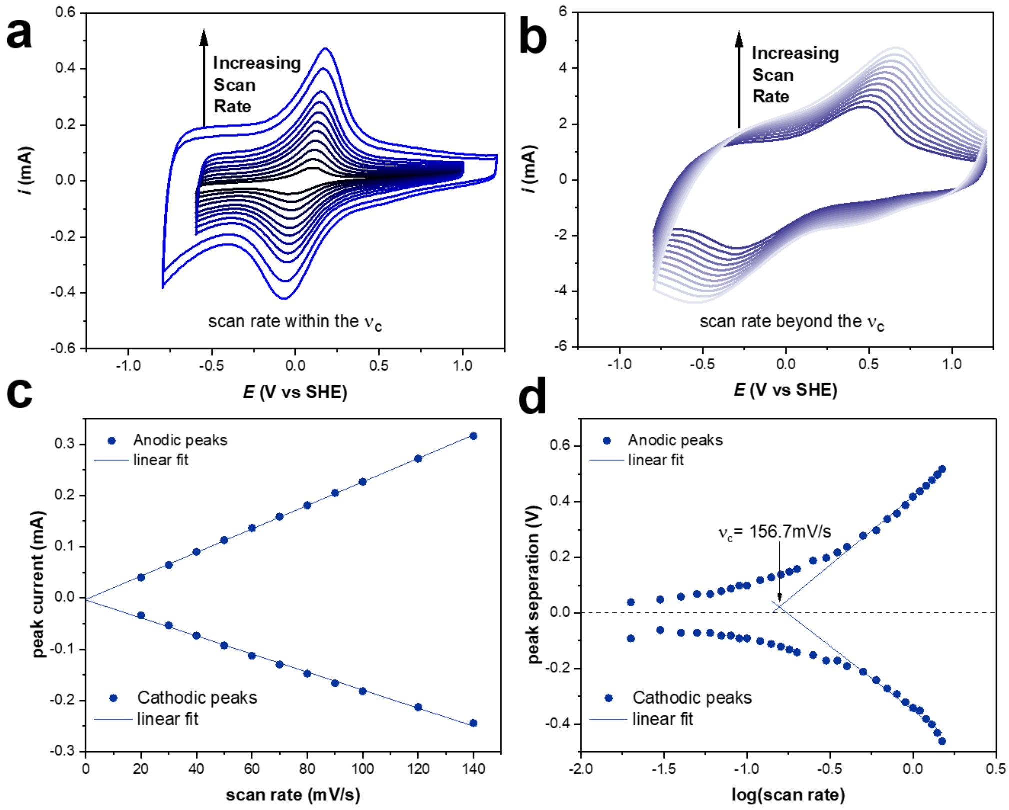

Figure 4: Electrochemical characterisation of Compound I on IO-mesoITO. **a,** Cyclic voltammetry of Compound I on IO-mesoITO surface within critical scan rate (20-140 mVs^{-1}); **b,** Cyclic voltammetry of Compound I on IO-mesoITO surface beyond critical scan rate (1000-2000 mV/s); **c,** Trumpet plot of Compound I derived from scan rate 20-2000 mVs^{-1}; **d,** Randles-Sevcik plot of Compound I on IO-mesoITO.

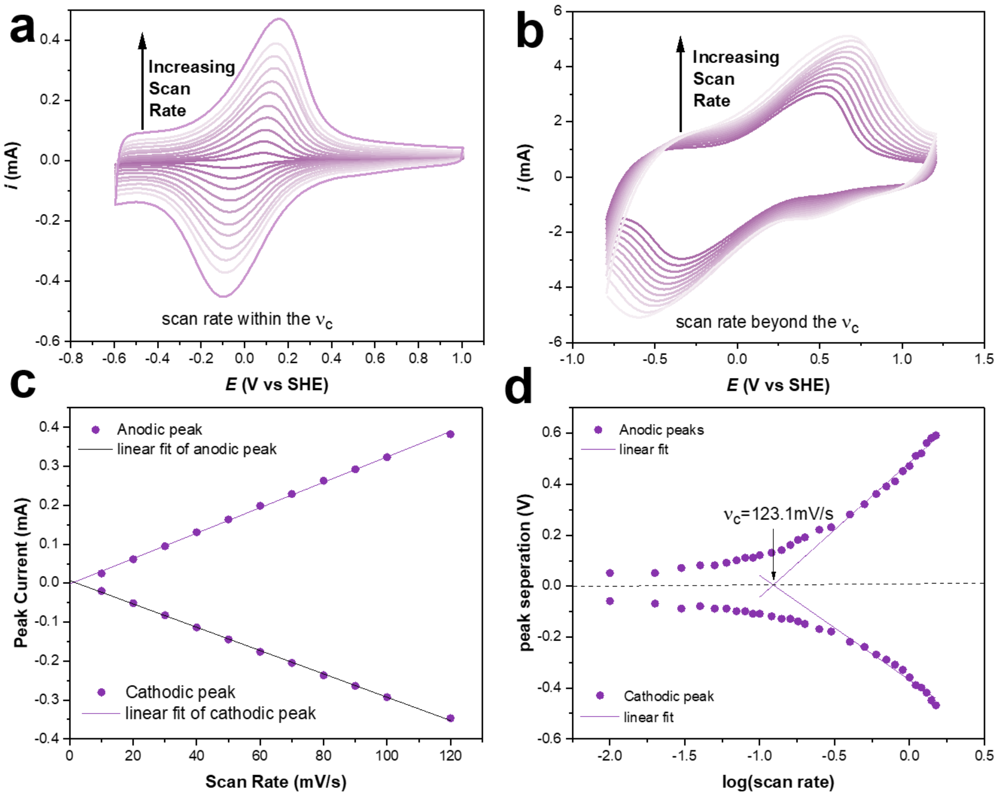

Figure 5: Electrochemical characterisation of Compound II on IO-mesoITO. **a,** Cyclic voltammetry of Compound II on IO-mesoITO surface within critical scan rate (10-120 mVs^{-1}); **b,** Cyclic voltammetry of Compound II on IO-mesoITO surface beyond critical scan rate (1000-2000 mV/s); **c,** Trumpet plot of Compound II derived from scan rate 10-2000 mVs^{-1}; **d**, Randles-Sevcik plot of Compound II on IO-mesoITO.

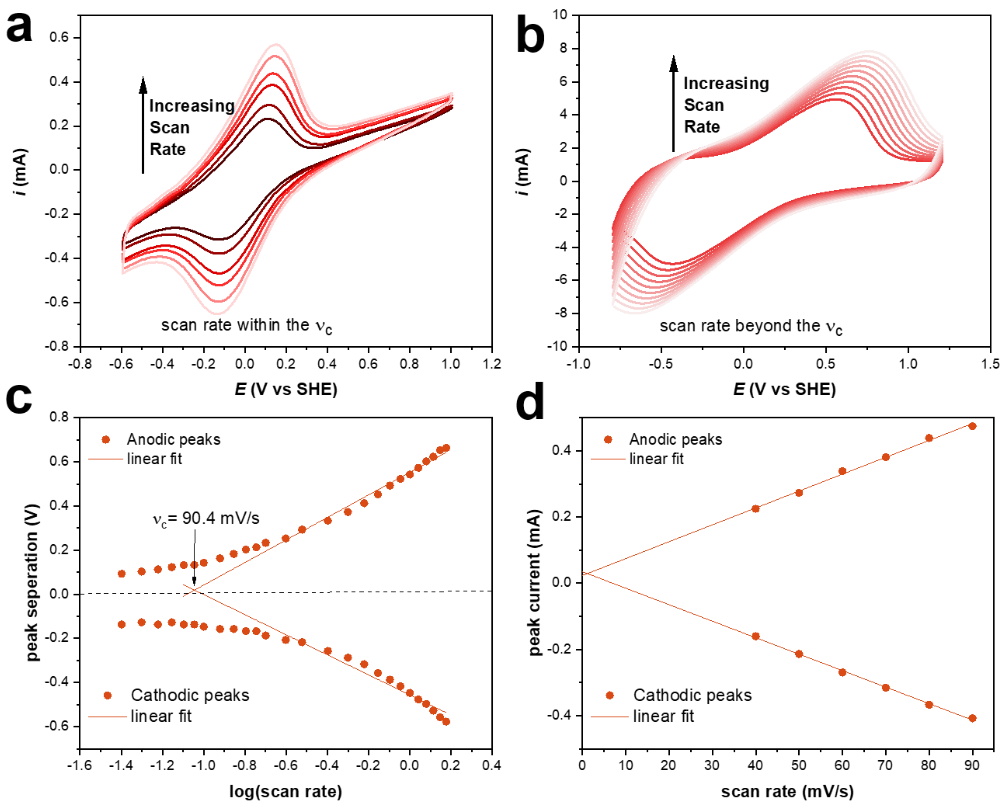

Figure 6: Electrochemical characterisation of Compound III on IO-mesoITO. **a,** Cyclic voltammetry of Compound III on IO-mesoITO surface within critical scan rate (40-90 mVs^{-1}); **b,** Cyclic voltammetry of Compound III on IO-mesoITO surface beyond critical scan rate (1000-1900 mVs^{-1}); **c,** Trumpet plot of Compound III derived from scan rate 20-2000 mVs^{-1}; **d,** Randles-Sevcik plot of Compound III on IO-mesoITO.

5 Electrochemical Kinetics Calculations (Compound II as an example)

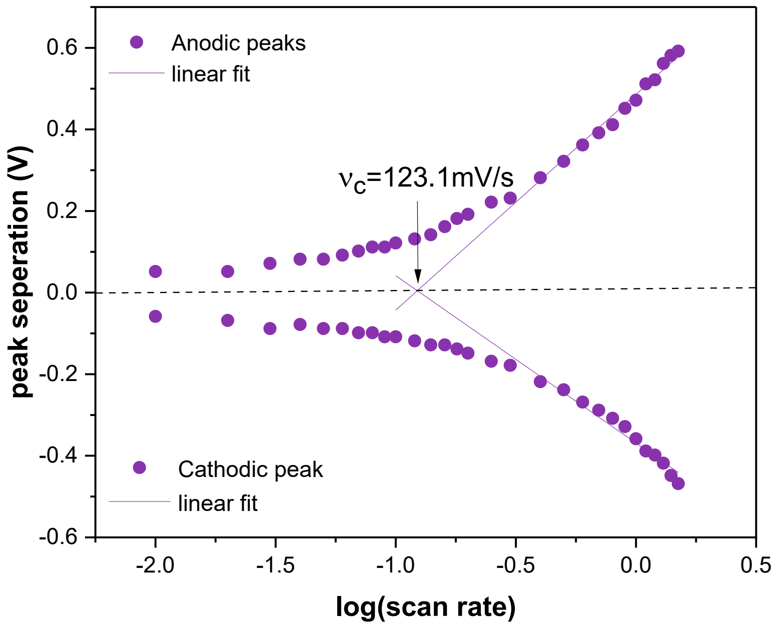

Figure 7: Trumpet plot of Compound II on IO-mesoITO.

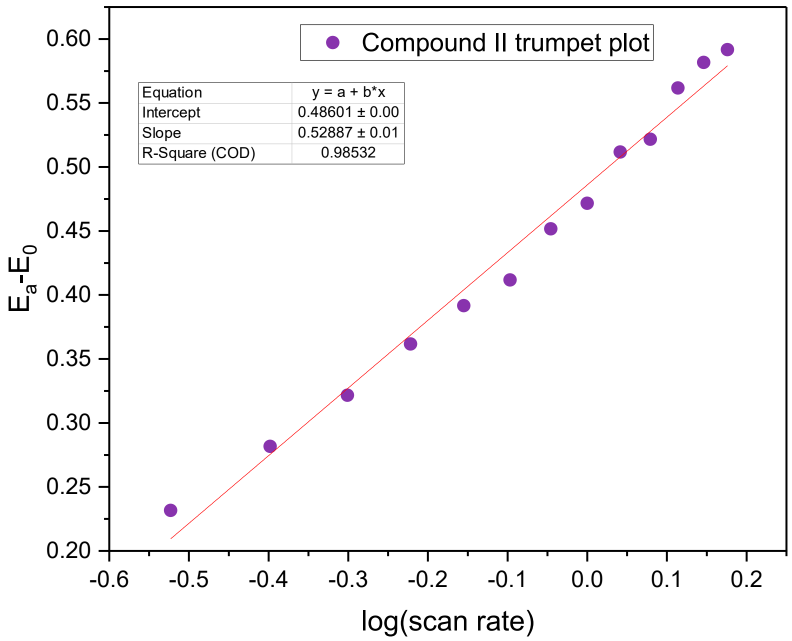

Figure 8: The selected region of trumpet plot for Compound II to calculate the $k_{app.}$

Rearrangement of the Laviron formalism:

$$E_{p,a} - E^{o'} = -\frac{2.3RT}{(1-\alpha)nF}\log\frac{(1-\alpha)nF}{RTk_{app\_A}}, \qquad \text{Eq. S1}$$

where $E_{p,a}$ is the potential of the anodic peak, $E^{o'}$ is the formal potential calculated by averaging the anodic and cathodic potentials at slow scan rates, v is the scan rate, α is the electron transfer coefficient, k_{app} is the apparent rate constant, R is the ideal gas constant, T is the absolute temperature, F is the Faraday constant, and n is the number of electrons transferred. n = 1; R = 8.3145 J/(mol·K); T = 298.15 K; F = 96,485.33 C/mol.

a) (1-α) is determined from the slope:

slope = $-\frac{2.3RT}{(1-\alpha)nF}$ = 0.528 $\Longrightarrow$ (1-α) = $\frac{2.3RT}{0.443nF}$ = 0.112

b) v_a is determined from the x-intercept:

$$\log(v_a) = -\frac{y\text{−}intercept}{slope} = -\frac{0.486}{0.528} \quad \Longrightarrow \quad v_a = 0.12V/s$$

Hence it follows that k_{app_a}:

$$k_{app\_a} = \frac{(1-\alpha)nFv_a}{RT} = \frac{0.112 * 96485 * 0.12}{8.31 * 298.15} = 0.523$$

6 Temperature dependence study

6.1 Influence of peak separation threshold on the β value

Since the β value is crucial for measuring the conductance decay of molecular wires, the β value we obtained here is based on the electron transfer rate rather than the conductance (as typically reported in the literature), with respect to molecular length. Given that the electron transfer rate could be slightly impacted by the ΔE threshold selection following the Laviron method, we sought to verify if the choice of the ΔE threshold affects the β value.

When determining the electron transfer rate using a trumpet plot, one crucial parameter is the peak separation threshold. This threshold dictates which region of data within the trumpet plot will be used for fitting, ultimately influencing the calculation of the electron transfer rate. Typically, a peak separation of ≥ 200 mV is used for fitting and calculation of electron transfer rate, though slight adjustments to the threshold may be made based on experimental data. While it is known that varying the peak separation threshold can lead to slight differences in the calculated electron transfer rate, it is essential to understand whether these variations affect the β value. Ensuring the robustness of the β value obtained from electrochemical methods is crucial. To address this, we used different peak separation thresholds (0.2, 0.3, 0.4, 0.5, 0.6) to calculate the electron transfer rate of four compounds (Figures 9-12) and plotted k_{app} as a function of molecular length (the β plot). The β value, determined by the slope of the β

plot, indicates that the choice of peak separation threshold does not impact the β value, as demonstrated in Figure 13.

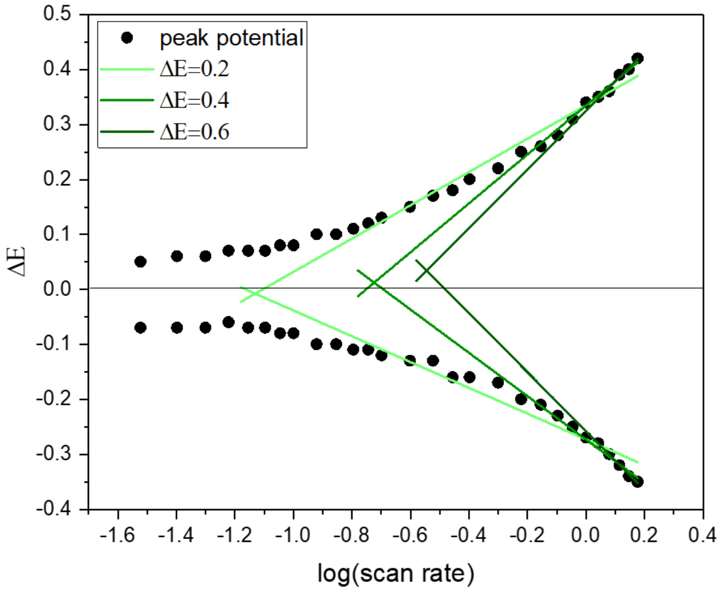

Figure 9: Trumpet plot for aminoferrocene on IO-mesoITO with fits to determine k_{app} based on different values for the ΔE threshold.

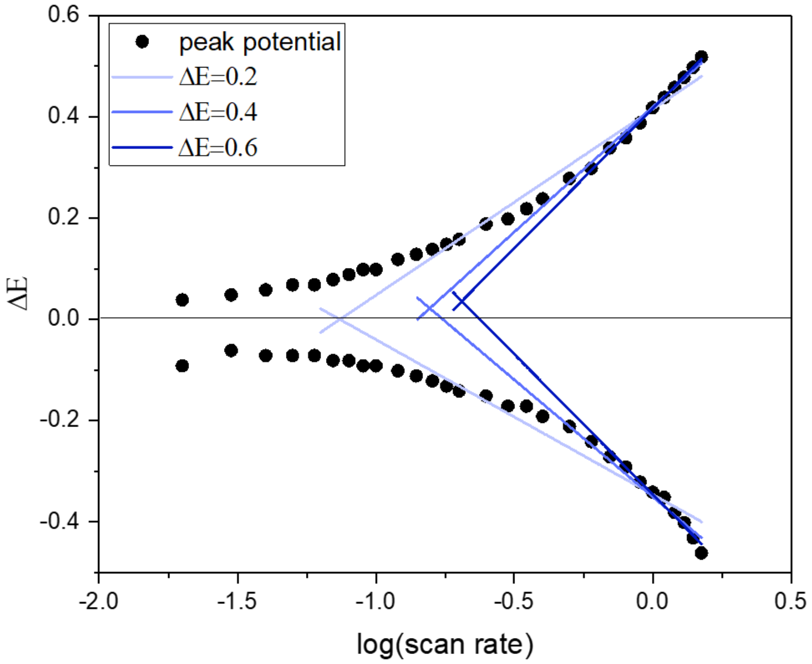

Figure 10: Trumpet plot for Compound I on IO-mesoITO with fits to determine k_{app} based on different values for the ΔE threshold.

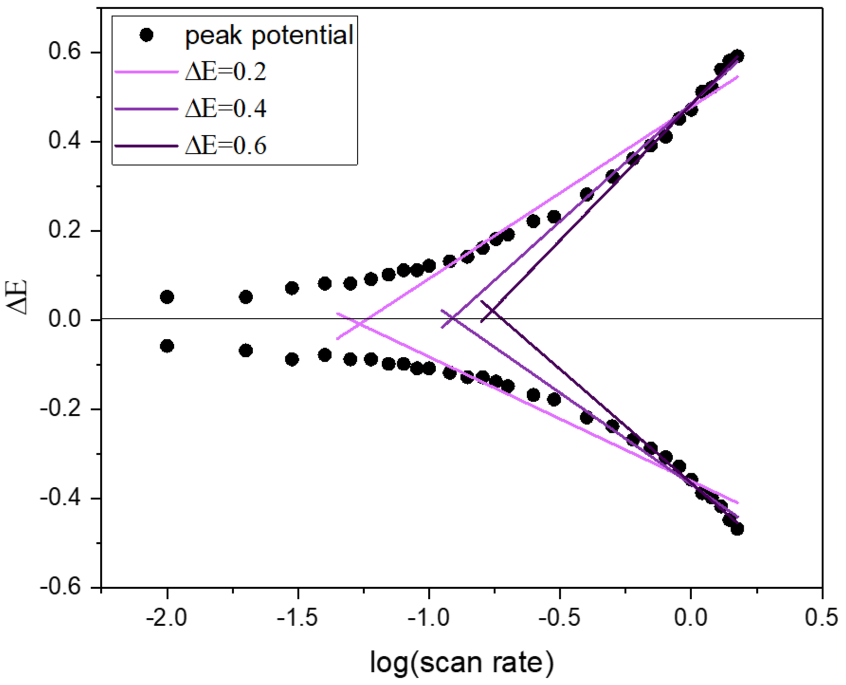

Figure 11: Trumpet plot for Compound II on IO-mesoITO with fits to determine k_{app} based on different values for the ΔE threshold.

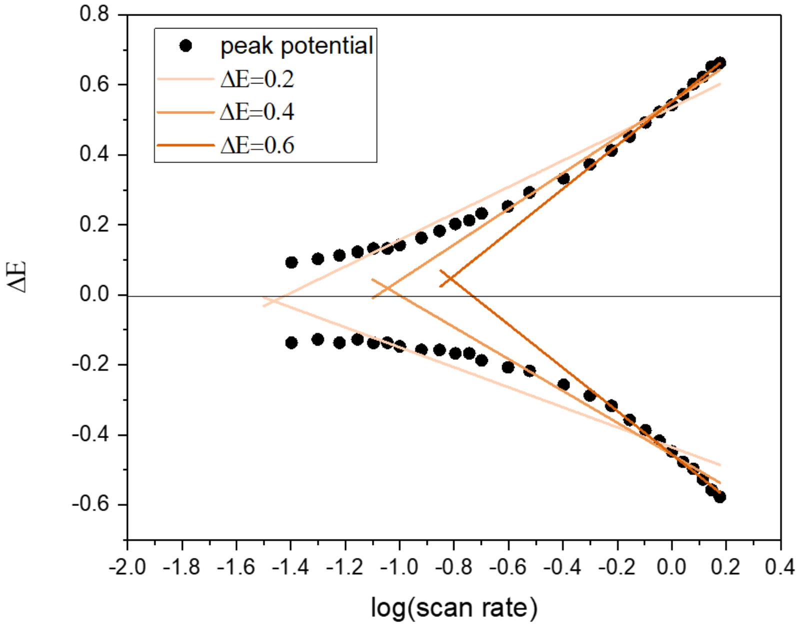

Figure 12: Trumpet plot for Compound III on IO-mesoITO with fits to determine k_{app} based on different values for the ΔE threshold.

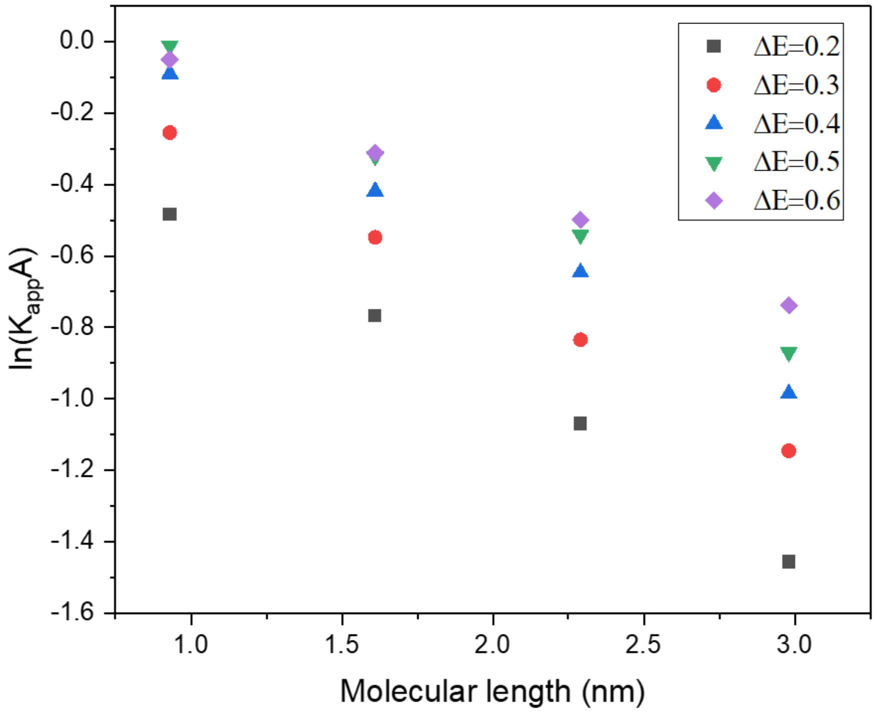

Figure 13: Investigation into the effect of the chosen ΔE threshold (Figures 9-12) on the β value.

6.2 Temperature-dependence study of electron transfer mechanism

There are several terms within the scientific literature for what is commonly referred to as “quantum tunneling”. Here we define quantum tunneling as the process of quantum particles passing through a potential energy barrier without first gaining the energy required to overcome this barrier. This process is sometimes referred to as “coherent tunneling” and particles that exhibit this behaviour are generally described as being within a “Sommerfeld regime” - a regime where quantum effects dictate ET properties with only a weak temperature dependence[1]. A key experimental feature of this quantum tunneling process, in addition to a lack of temperature dependence is also the lack of an activation energy. For clarity, we will refer to such processes simply as “tunneling”.

A second type of ET processes may occur at higher temperatures, where a stronger thermal dependence on ET rate is observed and the kinetics are described using activation energy in a more classical picture[2]. This regime is described as the “Arrhenius-like regime” and ET in this regime is sometimes referred to “incoherent tunneling”[3,4]. However, ET in this regime exhibits two- (or more) step kinetics, unlike the single-step kinetics of tunneling in the Sommerfeld regime described above, leading to this type of ET being known as “hopping”. Here, the electron traverses a

potential energy barrier, typically leading to the movement of charge and spatial relocation of the particle wavefunction (e.g. from an electrode to a molecular orbital of a substrate. With this in mind, we choose to describe these two different types of ET mechanisms in the Sommerfeld (weakly T dependent) and Arrhenius-like (strongly T dependent) regimes as simply tunneling or hopping, respectively[5].

The theory of electron transfer in electrochemical systems is well described by Marcus theory[6,7]:

$$k_{ET} \propto \frac{1}{\left(e^{\left[\frac{(E-E_i)F}{RT}\right]}\right)} * e^{[-\beta L]} * e^{\left[\frac{-\Delta G^{\ddagger}}{RT}\right]}, \qquad \text{Eq. S2}$$

where E is applied potential, E_i is the Fermi level energy of the specified metal electrode, β is the tunneling decay constant, L is the distance of electron transfer and $-\Delta G^{\ddagger}$ is the activation energy. The expression consists of three primary terms: the first exponential term describes the potential for occupation of the Fermi level within the electrode; the second exponential function describes the dependence of electron transfer rate on the distance; the third function is dependent on the activation energy. This theory builds the fundamental connection between electron transfer rate and length of wire, as described by the Wentzel-Kramers-Brillouin approximation for tunneling through a rectangular barrier:

$$\beta \propto \sqrt{2m\frac{|E_F - E_{MO}|}{\hbar^2}}. \qquad \text{Eq. S3}$$

Here m is the electron mass E_F is the Fermi level of the electrode, E_{MO} is the energy of a participating molecular orbital of a molecular wire and ħ is the reduced Planck constant. Eq S3 shows how alignment of the Fermi level of the electrode with the acceptor MO of the molecular wire decreases the attenuation value β[8]. For many systems exhibiting tunneling ET mechanisms, β > 0.2 A^{-1}, with the lowest values typically being associated with molecular wires comprised of conjugated π systems[9]. However, in certain systems (e.g. conjugated systems with porphyrin or alkyne groups) β ~ 0.01 A^{-1} – an order of magnitude smaller than what is usually observed for alkanethiols, oligothiophenes and oligophenylenes[10,11]. To explain the ET properties of systems with such low β values, their ET rate as a function of temperature is examined. Typically, tunneling is expected to exhibit ET rates independent of temperature and hopping is expected to exhibit thermal dependence. However, in certain systems at elevated temperatures, thermally-activated tunneling arising from Fermi level broadening can result in Arrhenius-like behaviour that can easily be misconstrued as hopping[12]. If incoherent tunneling (hopping) dominates, the temperature dependence from monomers to longer compounds is expected to exhibit a decreasing trend. A summary of the differences in properties between tunneling and hopping mechanisms is summarised in Table 1 below.

Table 1. Comparison of tunneling, thermally-activated tunneling and hopping ET mechanisms.

	**Tunneling**	**Thermally-activated tunneling**	**Hopping**
**Mechanism**	Non-zero probability of electron passing through a potential energy barrier without needing to overcome the energy barrier	Combines thermal excitation and quantum tunnelling	Electron transfer via intermediate localised states (molecular orbitals)
**Distance dependence**	Exponential decay in probability with increasing distance	Exponential decay in probability with increasing distance	Comparatively small distance dependence
**Temperature dependence**	Negligible at low T (Sommerfeld regime), significant at high T (Arrhenius-like)	Temperature-dependent conductivity	Temperature-dependent rate constant (Arrhenius)

The Arrhenius equation is given by:

$$k = Ae^{\frac{-E_a}{RT}}, \qquad \text{Eq. S4}$$

where k is the observed rate constant, A is the pre-exponential factor, E_a is the activation energy, R is the universal gas constant and T is temperature. To understand the ET mechanisms in molecular wires, temperature-dependence studies can be conducted to determine whether Arrhenius or Arrhenius-like behaviour is observed (Eq. S4 above). Below we report a systematic dataset for our compounds.

6.2.1 *Temperature-dependence study of Compound I on IO-mesoITO*

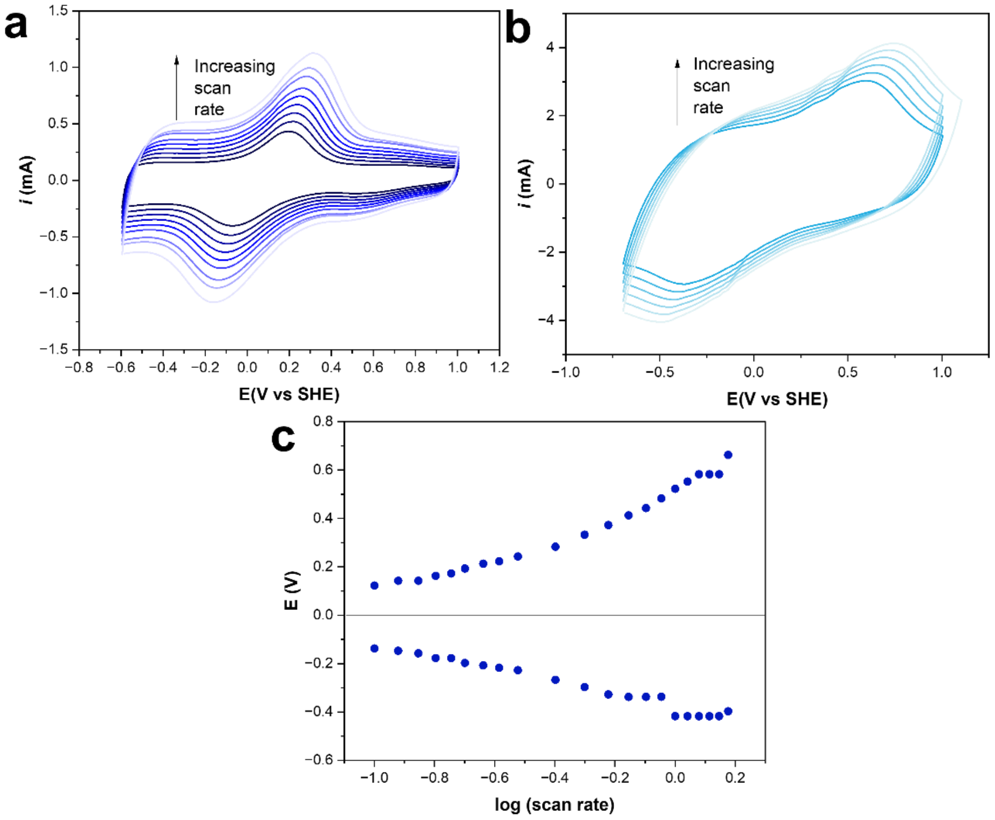

Figure 14. Electrochemical dataset of Compound I on IO-meso-ITO at 10°C. a, cyclic voltammograms of Compound I with scan rate 100-300 mVs^{-1} electrolyte: PBS buffer, pH7; **b,** cyclic voltammograms of Compound I with scan rate 1000-1500 mVs^{-1}; **c,** Trumpet plot of Compound I at 10°C.

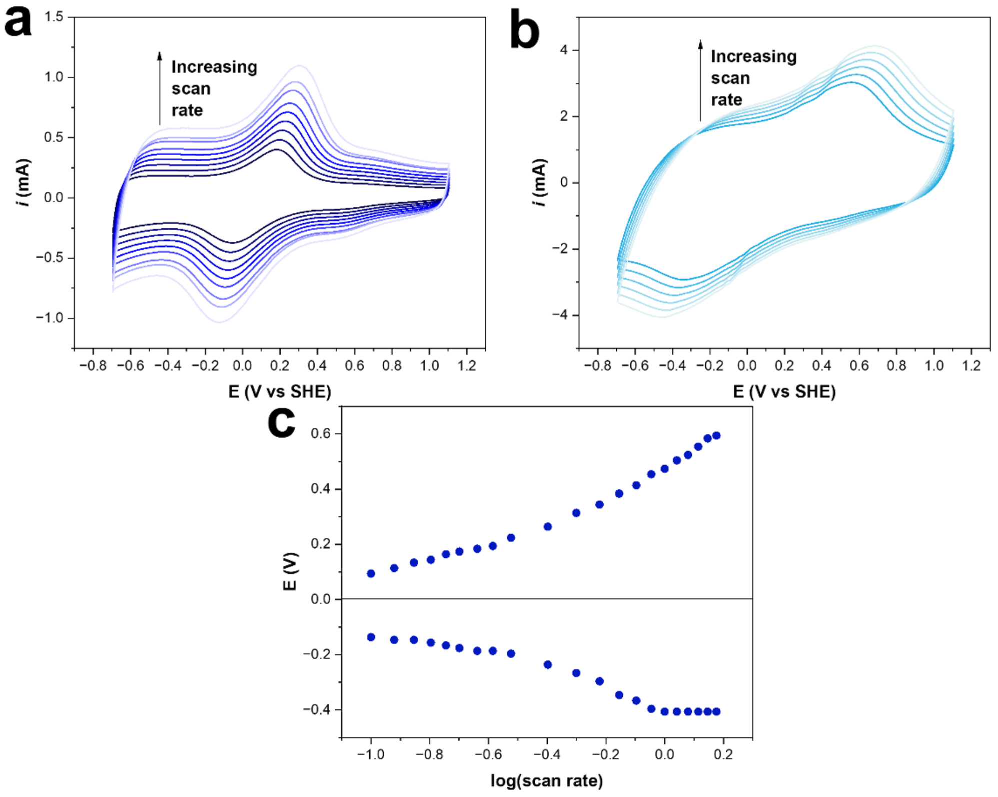

Figure 15: Electrochemical dataset of Compound I on IO-meso-ITO at 15°C. a, cyclic voltammograms of Compound I with scan rate 100-300 mVs^{-1}; electrolyte: PBS buffer, pH7; **b,** cyclic voltammograms of Compound I with scan rate 1000-1500 mVs^{-1}; **c,** Trumpet plot of Compound I at 15°C.

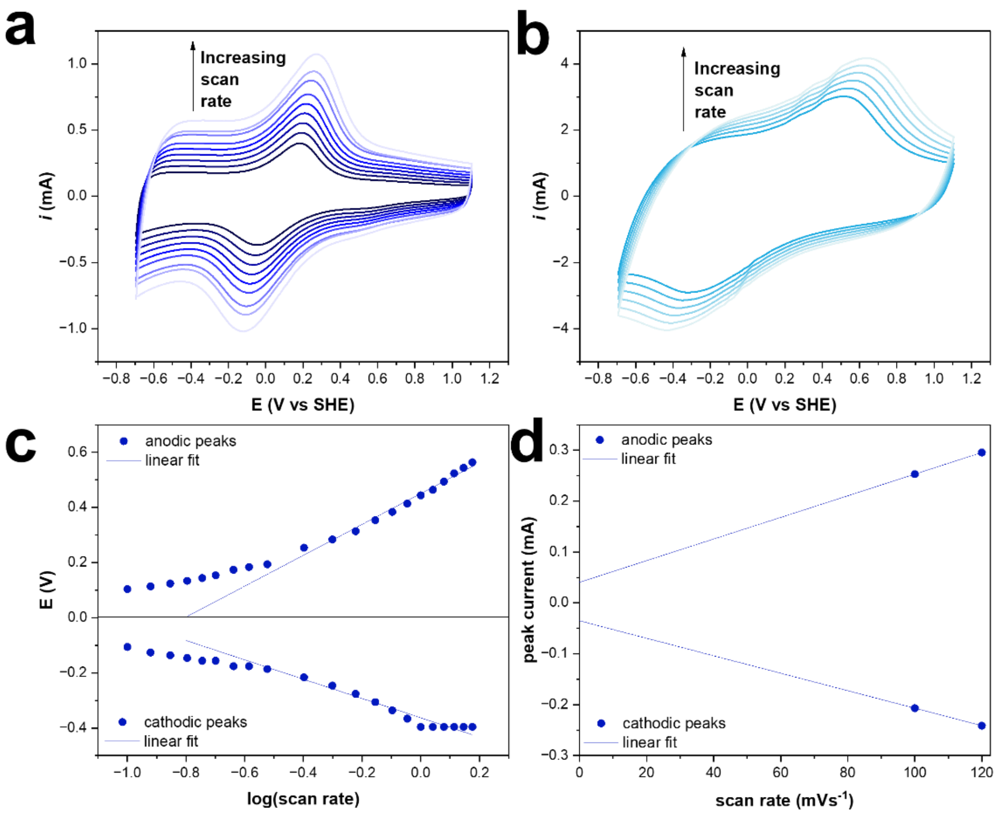

Figure 16: Electrochemical dataset of Compound I on IO-meso-ITO at 20°C. a, cyclic voltammograms of Compound I with scan rate 100-300 mVs^{-1}; electrolyte: PBS buffer, pH7; **b,** cyclic voltammograms of Compound I with scan rate 1000-1500 mVs^{-1}; **c,** Trumpet plot of Compound I at 20 °C. **d,** Randles-Sevcik plot of Compound I at 20°C.

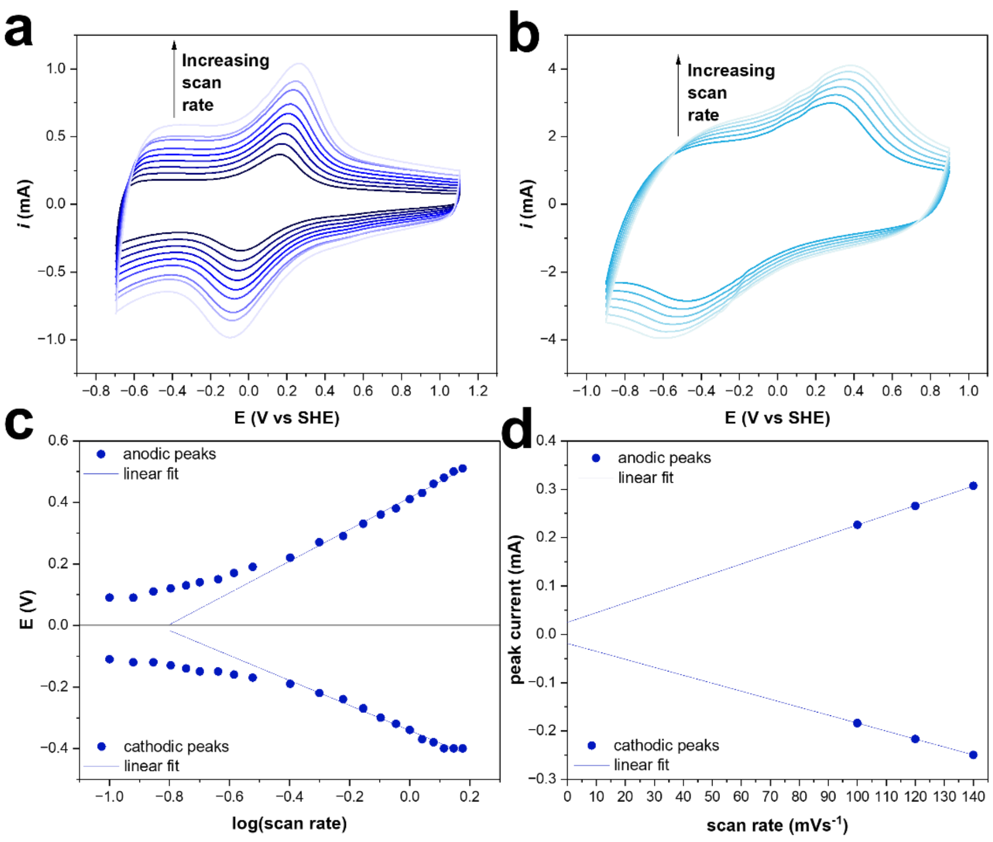

Figure 17: Electrochemical dataset of Compound I on IO-meso-ITO at 25°C. a, cyclic voltammograms of Compound I with scan rate 100-300 mVs^{-1}; electrolyte: PBS buffer, pH7; **b,** cyclic voltammograms of Compound I with scan rate 1000-1500 mVs^{-1}; **c,** Trumpet plot of Compound I at 25°C. **d,** Randles-Sevcik plot of Compound I at 25°C.

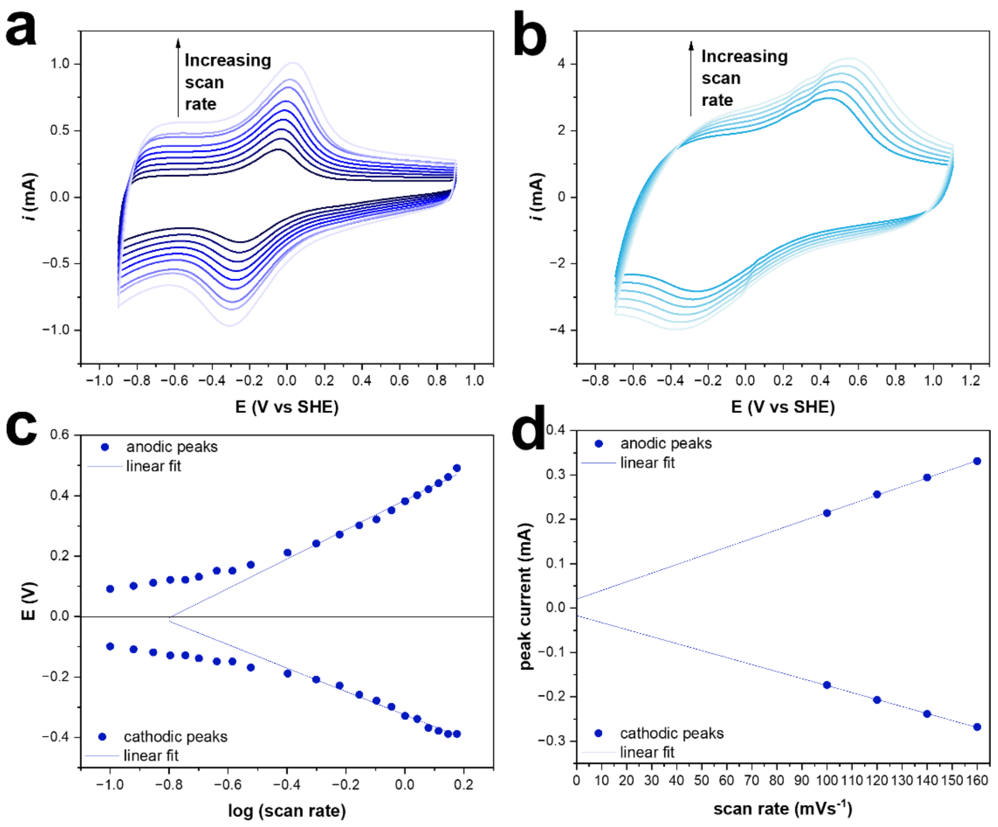

Figure 18: Electrochemical dataset of Compound I on IO-meso-ITO at 30°C. **a,** cyclic voltammograms of Compound I with scan rate 100-300 mVs^{-1}; electrolyte: PBS buffer, pH7; **b,** cyclic voltammograms of Compound I with scan rate 1000-1500 mVs^{-1}; **c,** Trumpet plot of Compound I at 30°C. **d,** Randles-Sevcik plot of Compound I at 30°C.

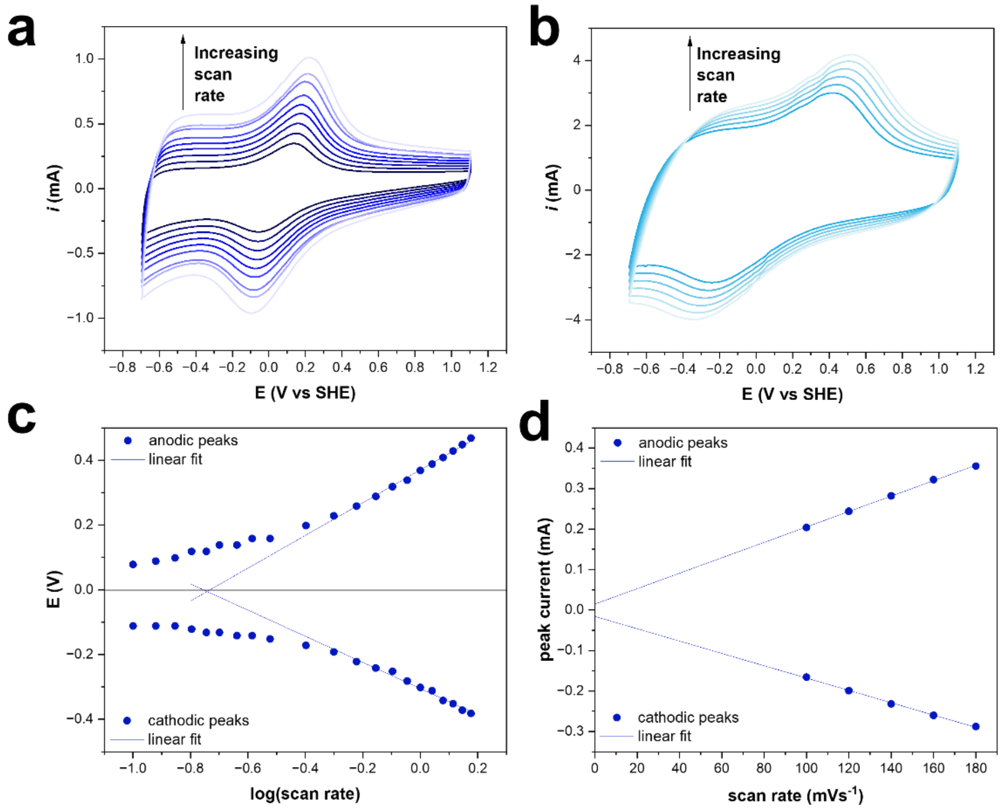

Figure 19: Electrochemical dataset of Compound I on IO-meso-ITO at 35°C. **a,** the cyclic voltammetry plot of Compound I with scan rate 100-300 mVs^{-1}; electrolyte: PBS buffer, pH7; **b,** the cyclic voltammetry plot of Compound I with scan rate 1000-1500 mVs^{-1}; **c,** Trumpet plot of Compound I at 35°C. **d,** Randles-Sevcik plot of Compound I at 35°C.

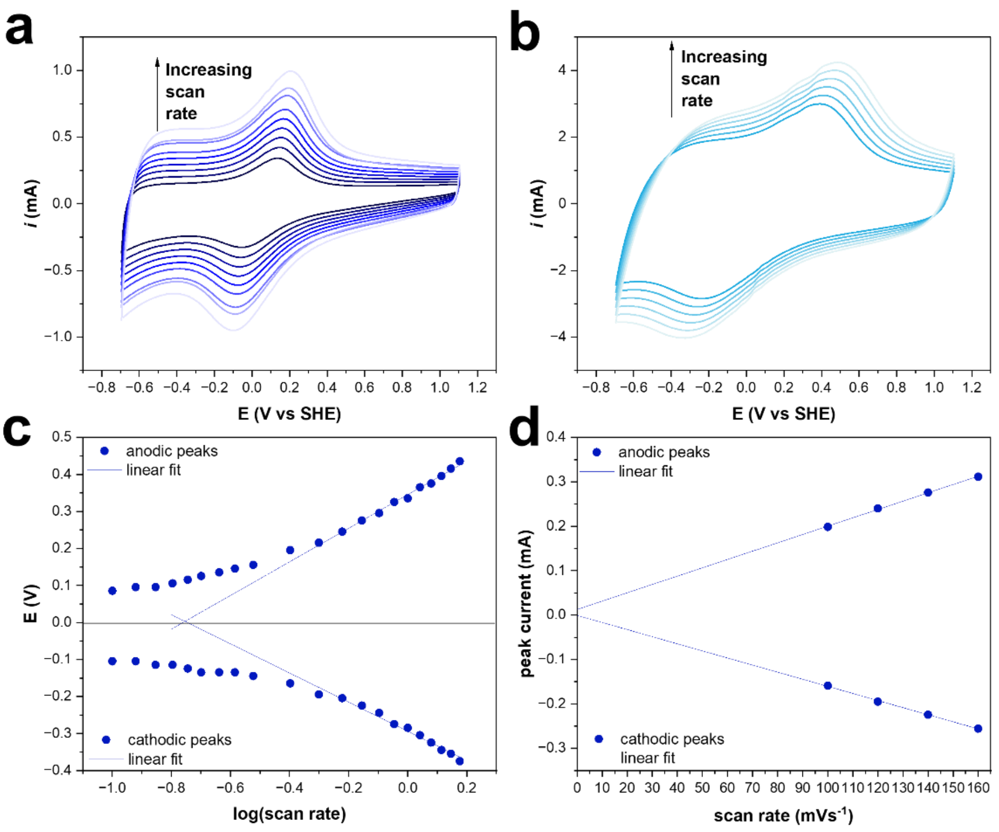

Figure 20: Electrochemical dataset of Compound I on IO-meso-ITO at 40°C. a, cyclic voltammograms of Compound I with scan rate 100-300 mVs^{-1}; electrolyte: PBS buffer, pH7; **b,** cyclic voltammograms of Compound I with scan rate 1000-1500 mVs^{-1}; **c,** Trumpet plot of Compound I at 40 °C. **d,** Randles-Sevcik plot of Compound I at 40°C.

Table 2: Temperature dependence study of Compound I on IO-mesoITO. α and 1-α are the electron transfer coefficients; k_{appA} and k_{appC} are the anodic and cathodic electron transfer rate constants, respectively.

Temperature(K)	E_0(V)	$E_{average}$(V)	v_c(mVs^{-1})	α	1-α	k_{appA}	k_{appC}
283	-0.14	-0.0995	79.2	0.11	0.212	0.464	0.355
288	-0.145	-0.1002	105.468	0.107	0.178	0.554	0.499
293	-0.135	-0.113	127.956	0.106	0.169	0.644	0.604
298	-0.131	-0.112	151.029	0.115	0.145	0.697	0.819
303	-0.142	-0.127	173.768	0.143	0.114	0.805	0.914
308	-0.148	-0.133	181.121	0.117	0.146	0.842	1.001
313	-0.156	-0.143	176.185	0.13	0.15	0.878	1.047

6.2.2 Temperature-dependence study of Compound I on S-ITO

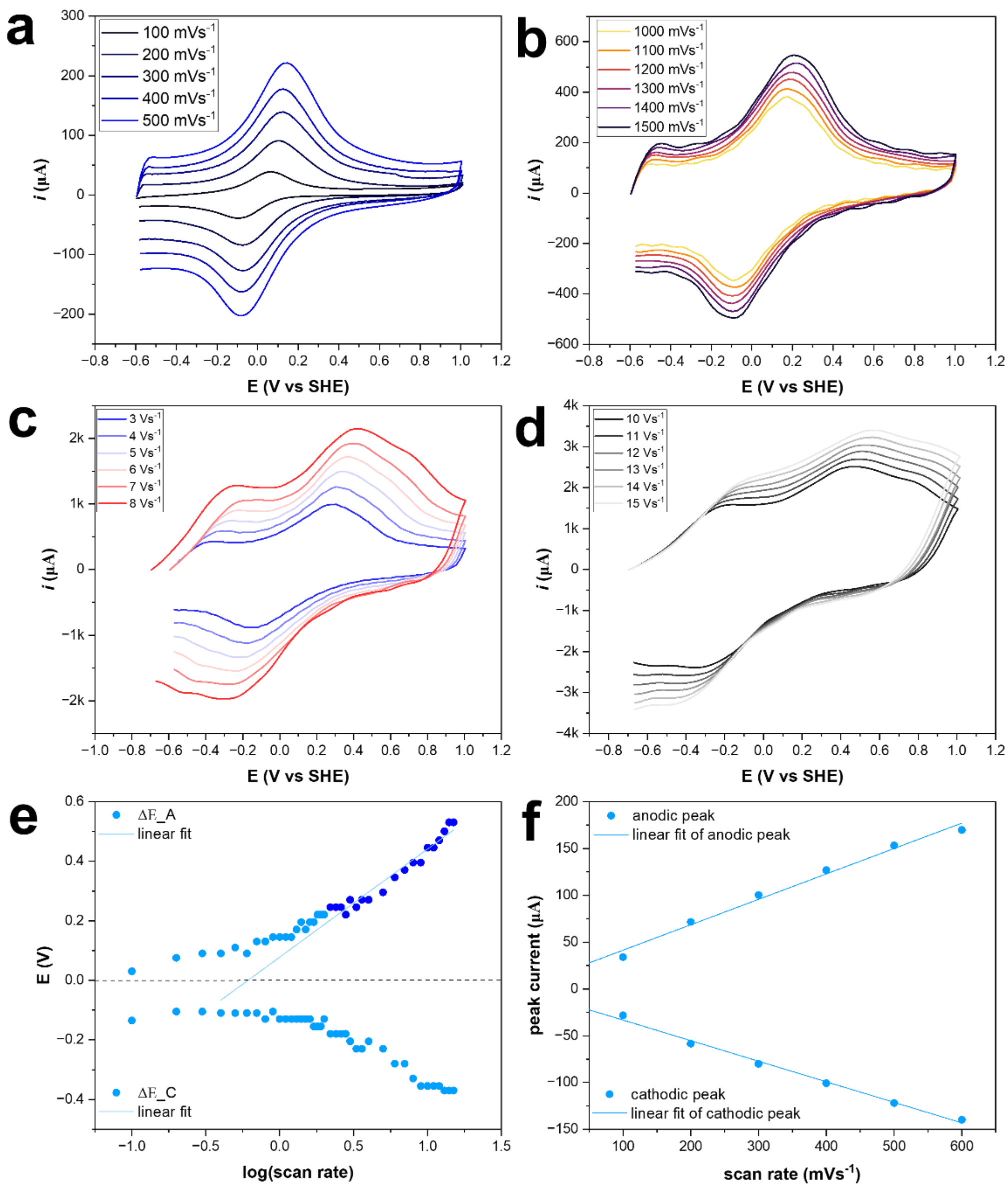

Figure 21: Electrochemical dataset of Compound I on S-ITO at 10°C. Cyclic voltammograms of Compound I on S-ITO with scan rates of **a,** 100-500 mVs^{-1}; **b,** 1000-1500 mVs^{-1}; **c,** 3000-8000 mVs^{-1}; **d,** 10000-15000 mVs^{-1}. **e,** Trumpet plot. Cathodic peak potentials were difficult to extract at high scan rates and therefore no fit is provided for the cathodic peaks. **f,** Randles-Sevcik plot. Electrolyte: PBS buffer, pH 7.

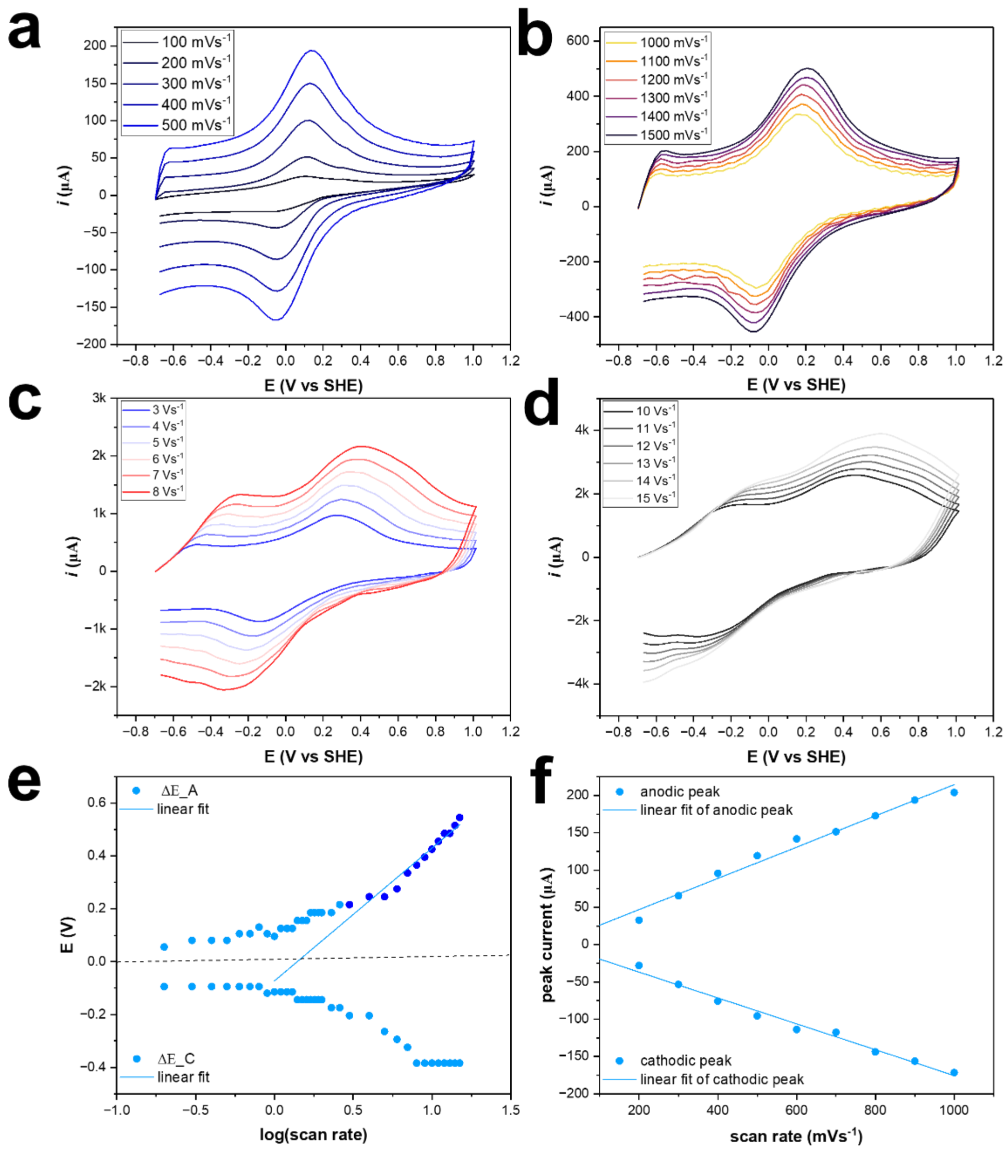

Figure 22: Electrochemical dataset of Compound I on S-ITO at 15°C. Cyclic voltammograms of Compound I on S-ITO with scan rates of **a,** 100-500 mVs^{-1};**b,** 1000-1500 mVs^{-1}; **c,** 3000-8000 mVs^{-1}; **d,** 10000-15000 mVs^{-1}. **e,** Trumpet plot. Cathodic peak potentials were difficult to extract at high scan rates and therefore no fit is provided for the cathodic peaks. **f,** Randles-Sevcik plot. Electrolyte: PBS buffer, pH 7.

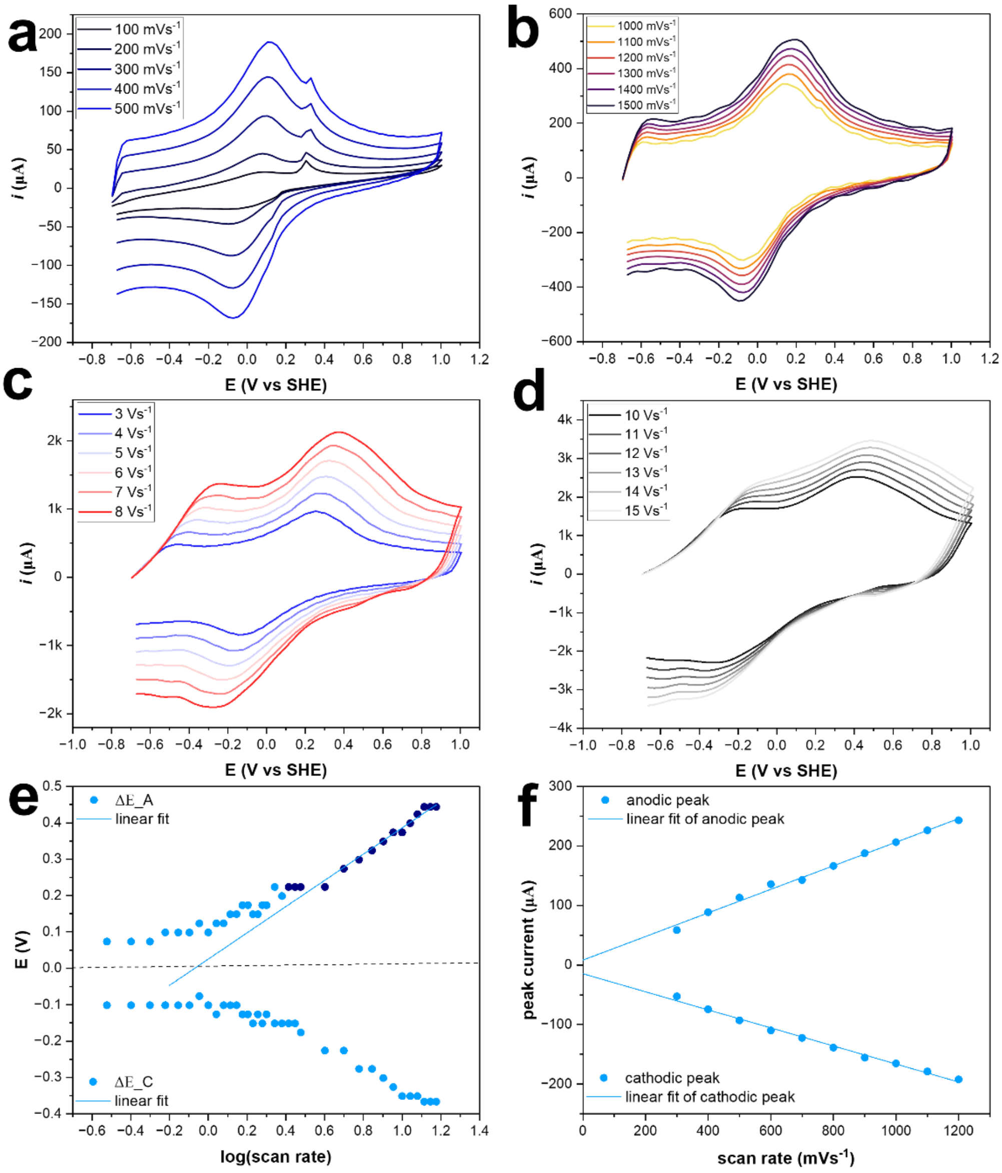

Figure 23: Electrochemical dataset of Compound I on S-ITO at 20°C. Cyclic voltammograms of Compound Ion S-ITO with scan rates of **a,** 100-500 mVs^{-1}; **b,** 1000-1500 mVs^{-1}; **c,** 3000-8000 mVs^{-1}; **d,** 10000-15000 mVs^{-1}; **e,** Trumpet plot, Cathodic peak potentials were difficult to extract at high scan rates and therefore no fit is provided for the cathodic peaks; **f,** Randles-Sevcik plot. Electrolyte: PBS buffer, pH 7.

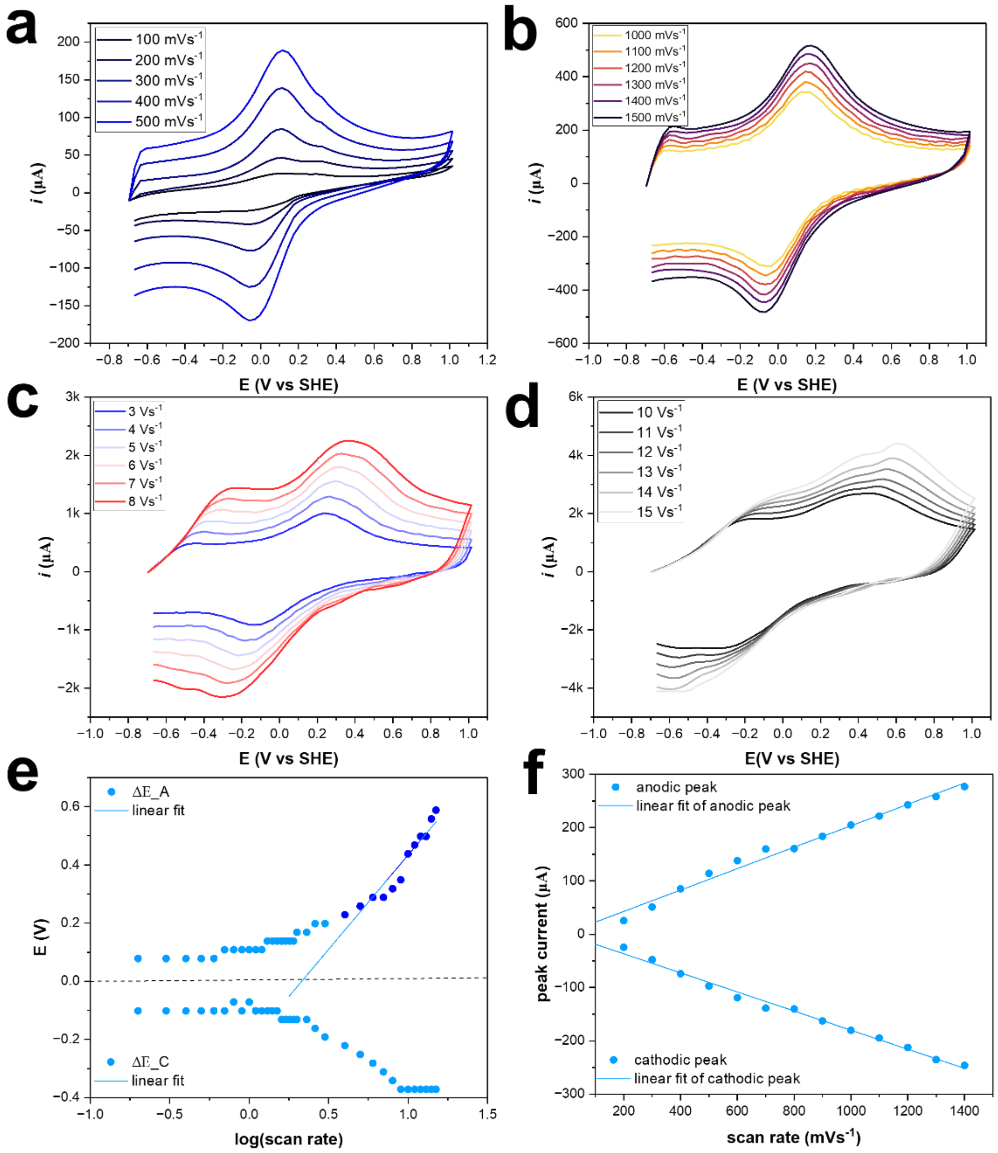

Figure 24: Electrochemical dataset of Compound I on S-ITO at 25°C. Cyclic voltammograms of Compound Ion S-ITO with scan rates of **a,** 100-500 mVs^{-1}; **b,** 1000-1500 mVs^{-1}; **c,** 3000-8000 mVs^{-1}; **d,** 10000-15000 mVs^{-1}; **e,** Trumpet plot, Cathodic peak potentials were difficult to extract at high scan rates and therefore no fit is provided for the cathodic peaks; **f,** Randles-Sevcik plot. Electrolyte: PBS buffer, pH 7.

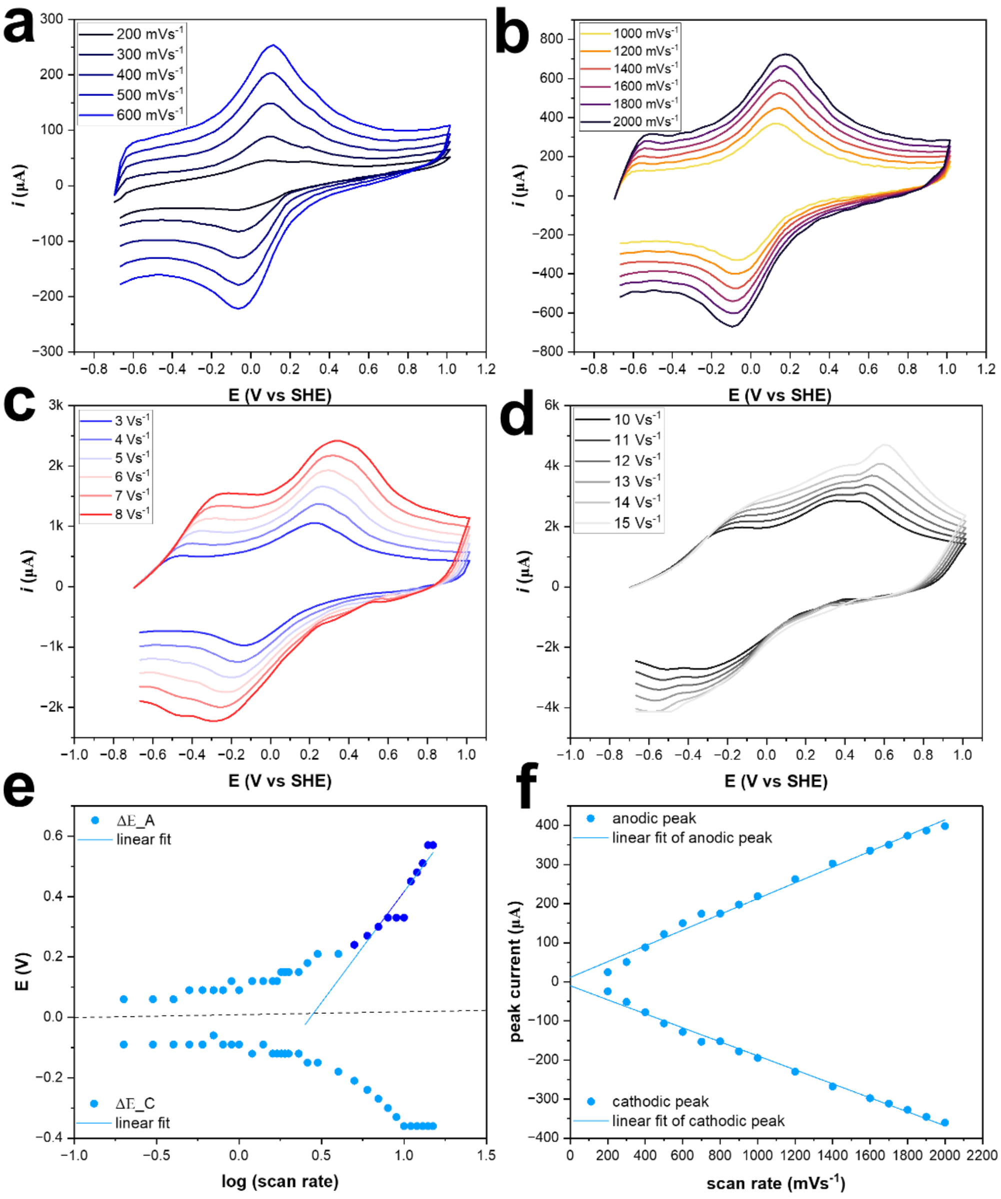

Figure 25: Electrochemical dataset of Compound I on S-ITO at 35°C. Cyclic voltammograms of Compound Ion S-ITO with scan rates of **a,** 100-500 mVs^{-1}; **b,** 1000-1500 mVs^{-1}; **c,** 3000-8000 mVs^{-1}; **d,** 10000-15000 mVs^{-1}; **e,** Trumpet plot; **f,** Randles-Sevcik plot. Electrolyte: PBS buffer, pH 7.

Table 3. Summary of the temperature dependence of kinetic parameters for Compound I on S-ITO. v_c is the critical scan rate; α and β are the electron transfer coefficients, and $\alpha + \beta = 1$ should be satisfied in an ideal scenario; k_{app} is the electron transfer rate and the electron transfer rate at the anode is used to represent the overall electron transfer rate.

T (K)	v_c (mVs^{-1})	k_{app} (s^{-1})	α	β
283	695	3.86	0.22	0.16
288	1037	6.38	0.20	0.11
293	1265	6.12	0.21	0.15
298	1389	7.59	0.21	0.10
308	2032	8.50	0.08	0.17

7 Surface Characterisation

7.1 Atomic Force Microscopy (AFM)

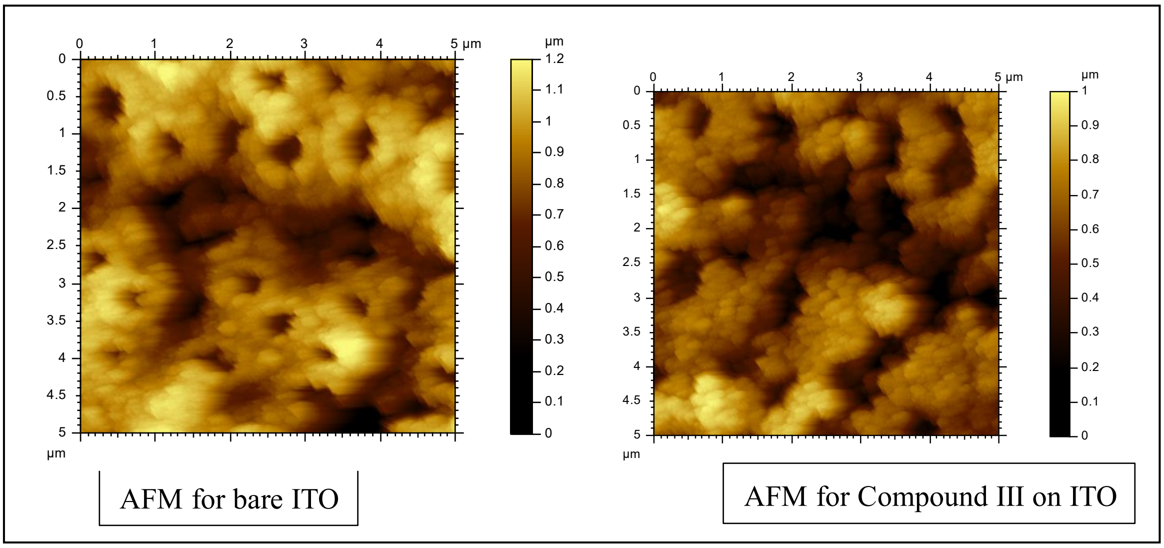

Figure 26: AFM images of bare IO-mesoITO (left) and Compound III (right) on IO-mesoITO.

7.2 *X-ray photoelectron spectroscopy (XPS)*

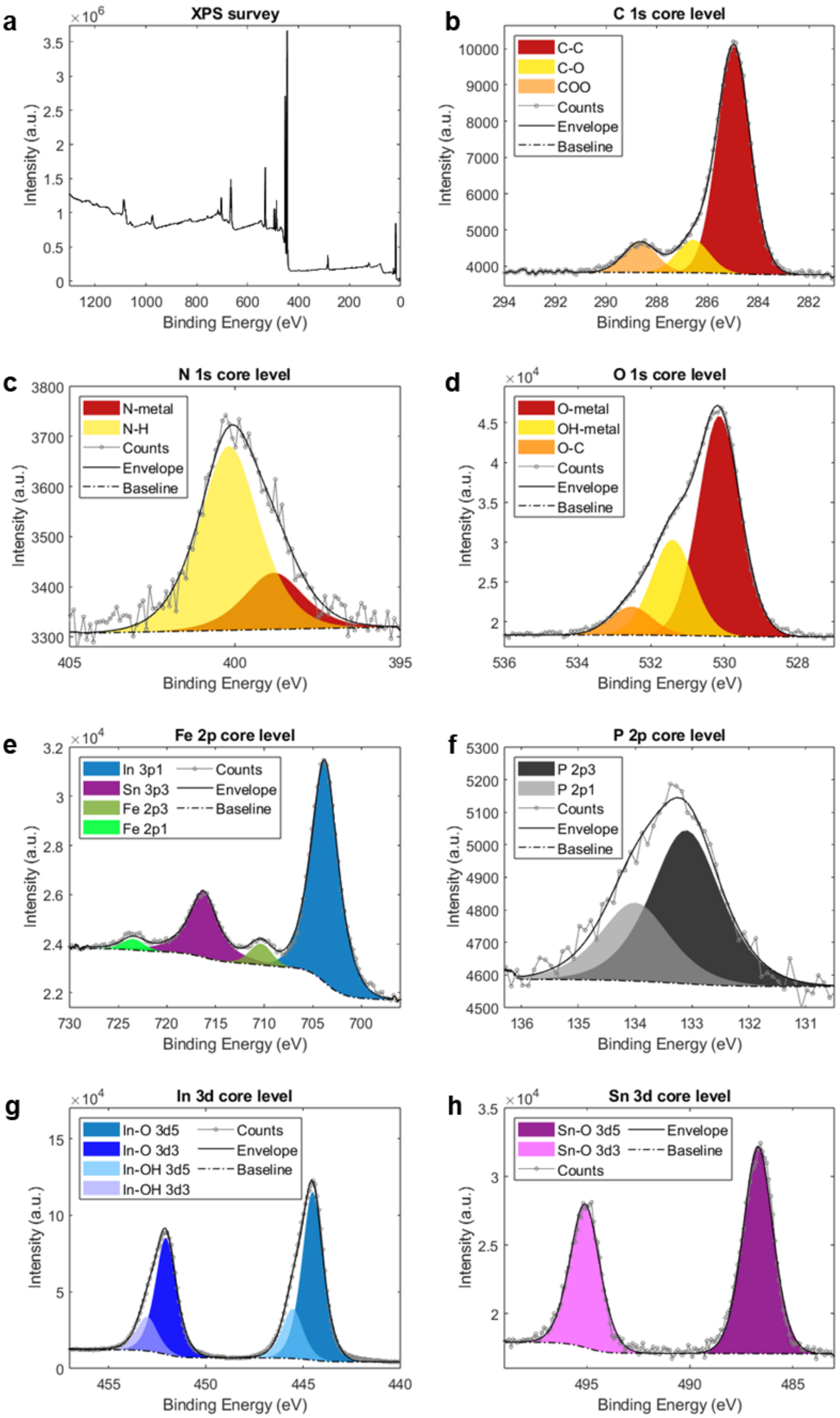

Figure 27: XPS spectra for aminoferrocene on IO-mesoITO. a) Full energy range survey spectrum; b) Sn 3d region; c) In 3d region; d) P 2p region; e) Fe 2p region; f) C 1s region; g) N 1s region; h) O 1s region.

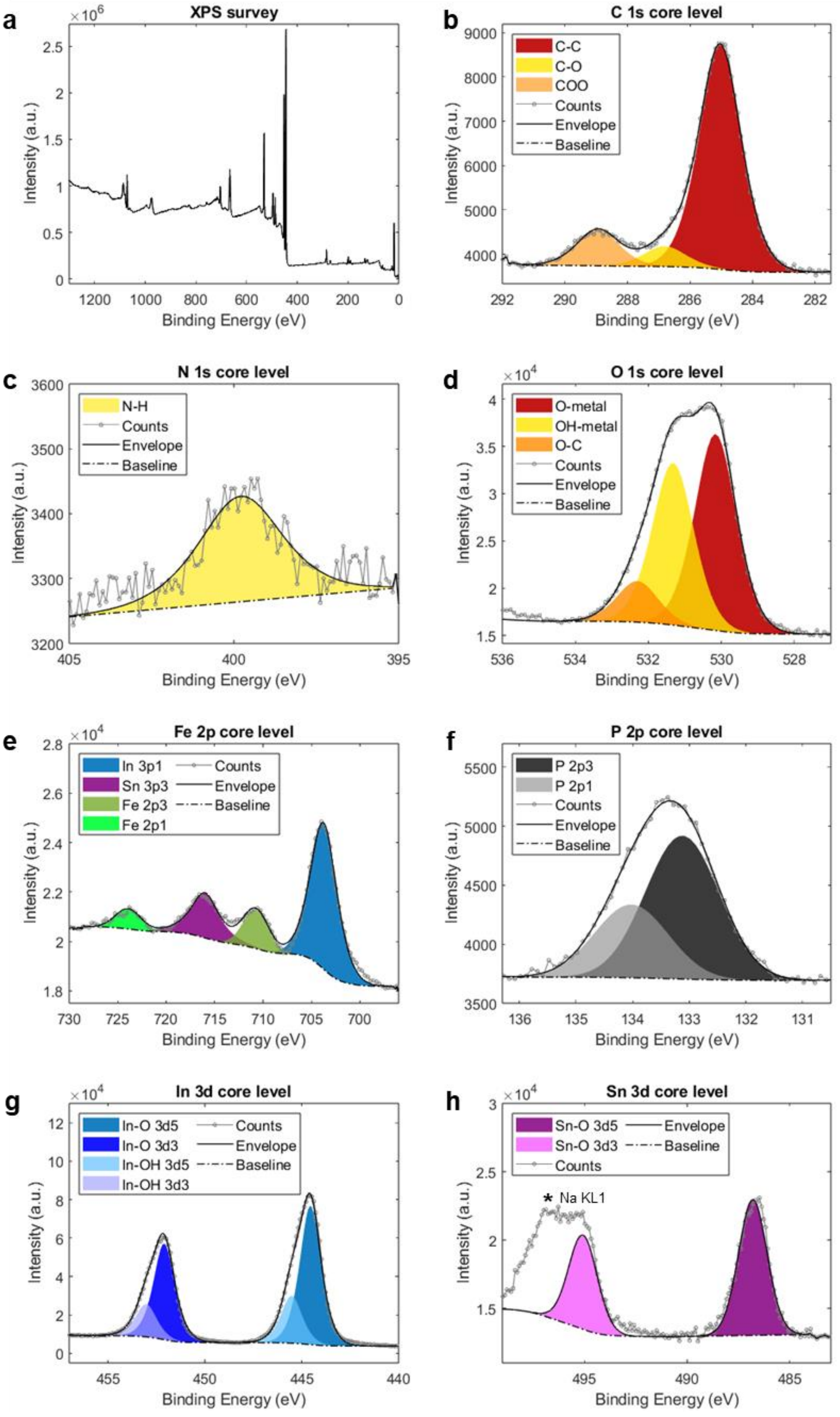

Figure 28: XPS spectra for Compound II on IO-mesoITO. a) Full energy range survey spectrum; b) Sn 3d region; c) In 3d region; d) P 2p region; e) Fe 2p region; f) C 1s region; g) N 1s region; h) O 1s region.

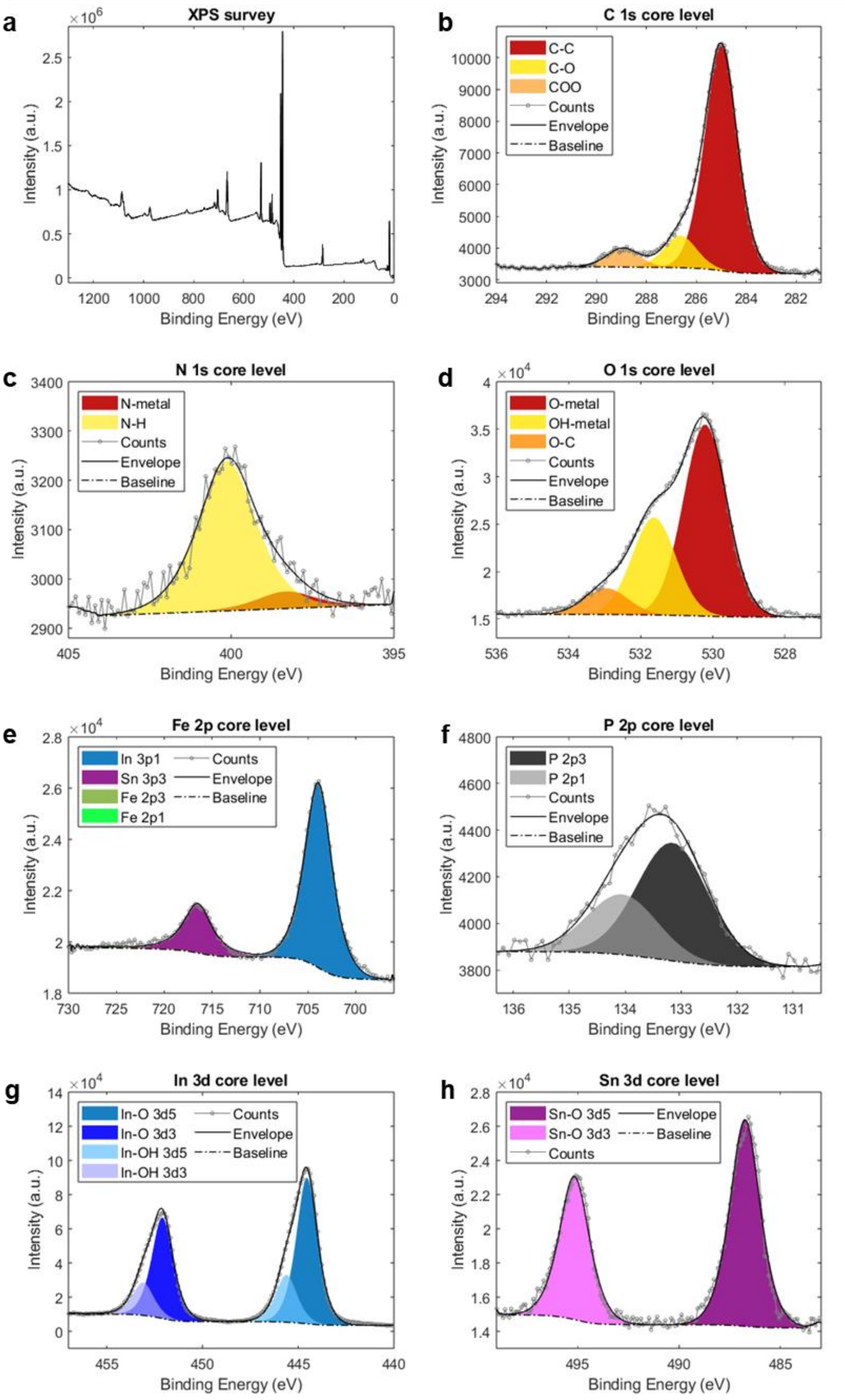

Figure 29: XPS spectra for blank IO-mesoITO electrode. a) Full energy range survey spectrum; b) Sn 3d region; c) In 3d region; d) P 2p region; e) Fe 2p region; f) C 1s region; g) N 1s region; h) O 1s region.

7.3 *BET (Brunauer-Emmett-Teller) measurements and surface coverage estimates*

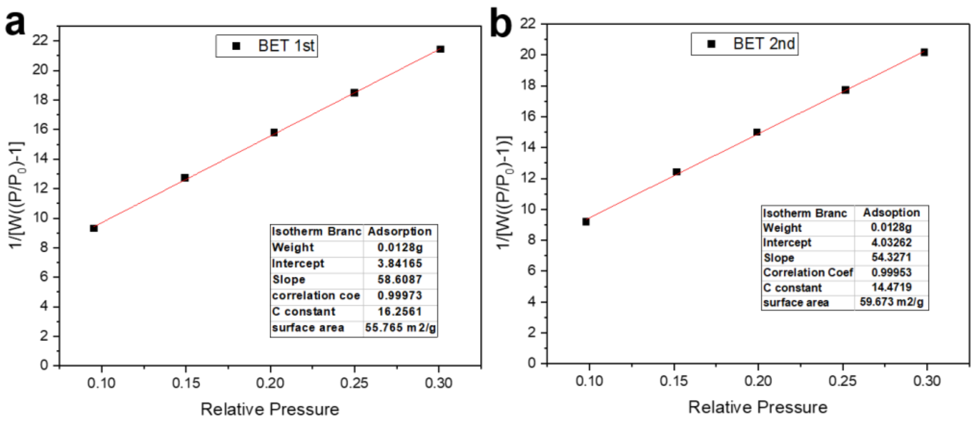

Figure 30: BET measurements used to determine the actual surface area in IO-meso ITO electrodes. (1) 1st measurement; (2) 2nd measurement.

Surface loadings were determined by integrating the peaks observed in the cyclic voltammograms to estimate the number of molecules (6.2 and 0.8 nmol for IO-mesoITO and S-ITO, respectively, see Extended Figure 1). For IO-mesoITO electrodes, the specific surface area (S = 57.701 m^2/g, average derived from two separate BET measurements as shown in Figure 30) combined with the weighed average mass of ITO on the electrode (0.00128 g) gave an estimate of the actual surface area (0.0739 m^2). For S-ITO electrodes, because the exact amount of the commercially supplied material loaded onto the substrate is difficult quantify, the surface area was estimated using its geometric dimensions (0.8*1.2 cm = 0.96 cm^2). Consequently, the surface coverage provided in Extended Figure 1a should be taken for guidance only. Surface coverages of Compound I on IO-mesoITO and S-ITO were therefore estimated to be 6.2 nmol * 6.022*10^{14} $nmol^{-1}$ / 0.0739*10^{18} nm^2 = 0.051 molecules/nm^2 and 0.8 nmol * 6.022 * 10^{14} $nmol^{-1}$ / 0.96*10^{14} nm^2 = 5.1 molecules/nm^2, respectively.

7.4 *Confocal microscopy*

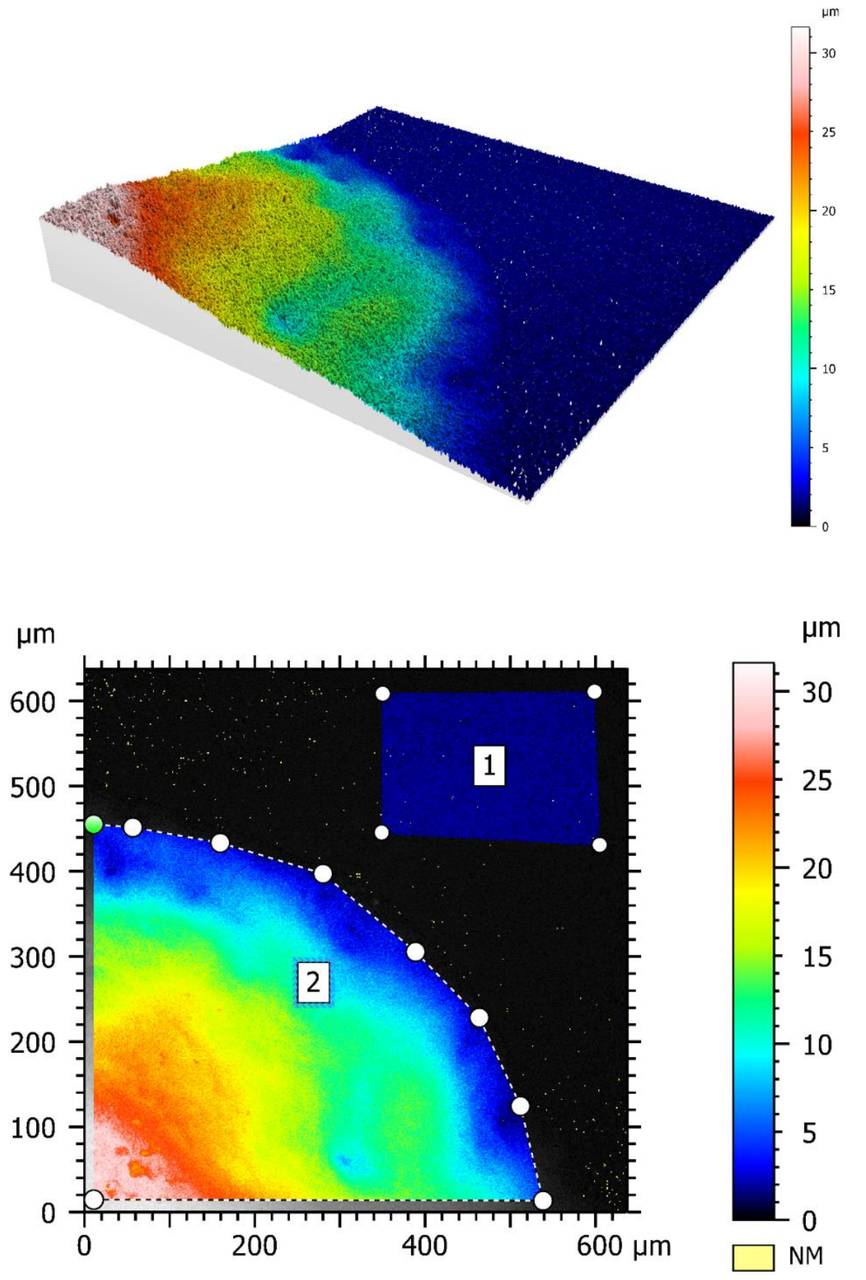

Differential parameters	P2 - P1	Unit	
Zmean(higher) - Zmean(lower)	11.34	µm	
Angle difference	3.397	°	

Plane parameters	Unit	Plane 1	Plane 2
Zmin	µm	0.000	0.000
ZMean	µm	1.607	12.95
Zmax	µm	3.667	30.97

Figure 31: Topography analysis of the IO-mesoITO electrode via confocal microscopy.

7.5 Kelvin Probe (KP) measurements

Table 4: KP measurements of different electrodes. Compounds I-III were anchored onto IO-mesoITO electrodes.

Sample	Work Function (eV)
IO-mesoITO	-4.6
Au	-4.9
Compound I	-4.43
Compound II	-4.31
Compound III	-4.37

8 Characterisation of molecular wires on S-ITO – contact angles and absorbance

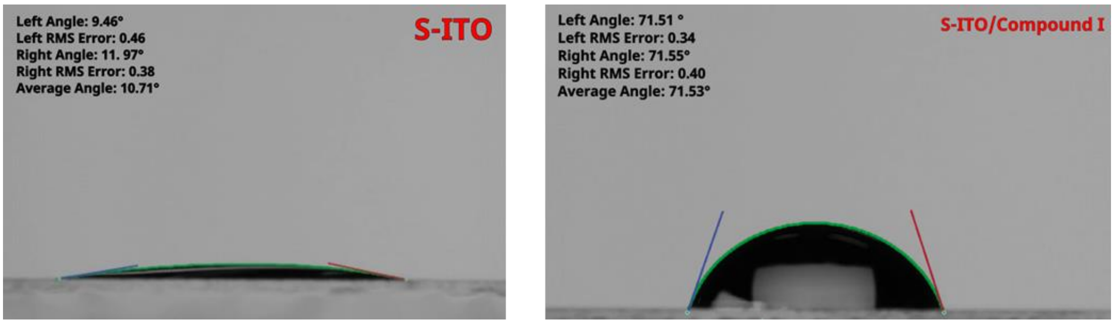

Figure 32: Water Contact angles on top of S-ITO (left) and S-ITO / Compound I films (right).

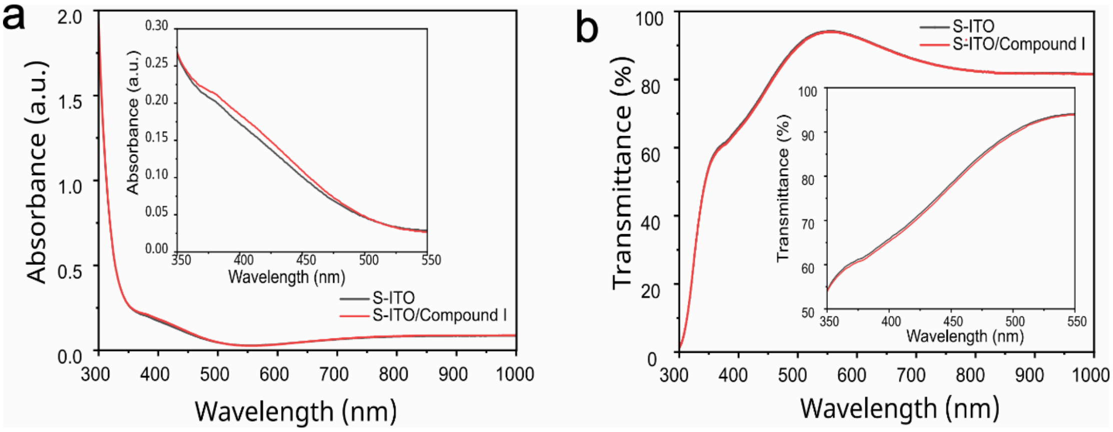

Figure 33: a, absorbance and **b,** transmittance spectra data of S-ITO and S-ITO / Compound I.

9 Characterisation of perovskite films deposited on S-ITO and S-ITO/Compound I

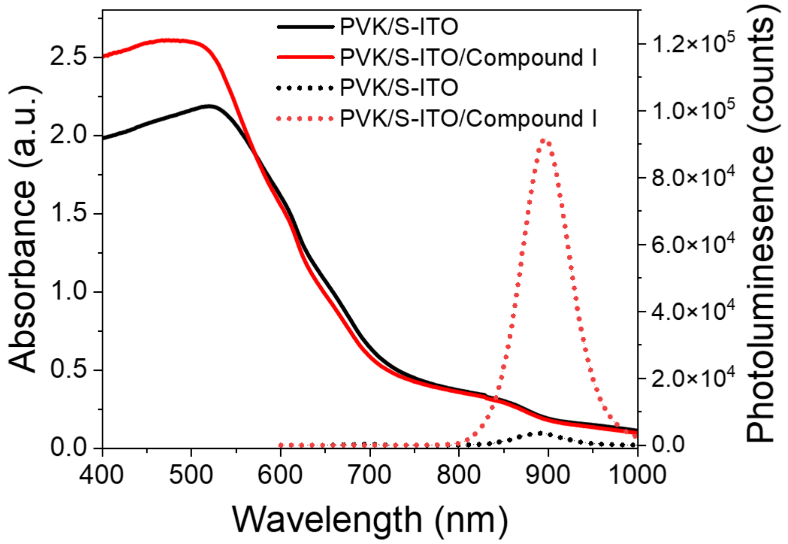

Figure 34: Optical spectroscopy; absorbance (solid) and emission (dotted) of $PEA_{0.2}FA_{0.8}SnI_3$ perovskite prepared on S-ITO substrates (black) and Compound I functionalised S-ITO (red).

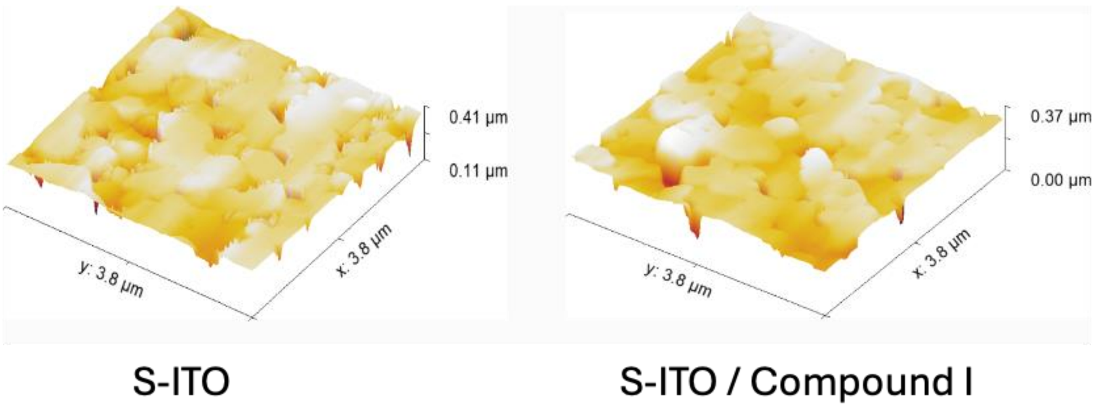

Figure 35: AFM topology of $(PEA)_{0.2}(FA)_{0.8}SnI_3$ perovskite films on different substrates (left on the S-ITO and right on the S-ITO/Compound I).

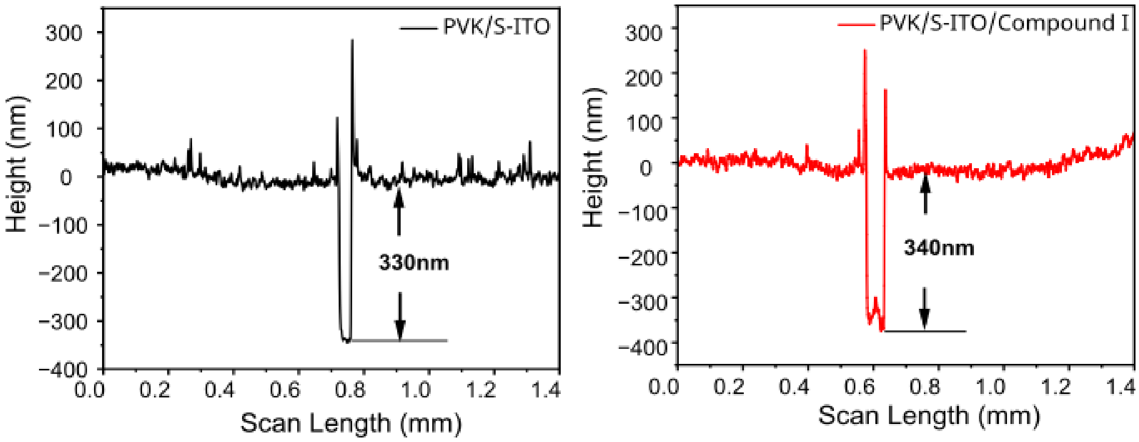

Figure 36: The thickness of $(PEA)_{0.2}(FA)_{0.8}SnI_3$ perovskite films on different substrates (left on the S-ITO and right on the S-ITO/Compound I) was measured via a profilometer.

10 Electronic characterisation of perovskite devices

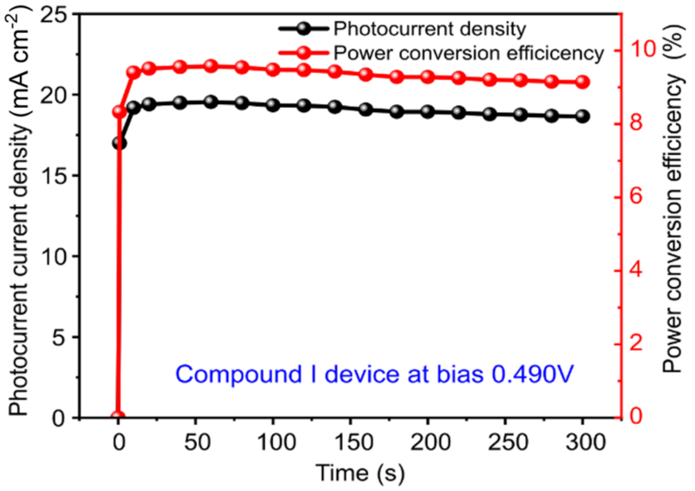

Figure 37: Stabilised power output measurements collected over 300 s.

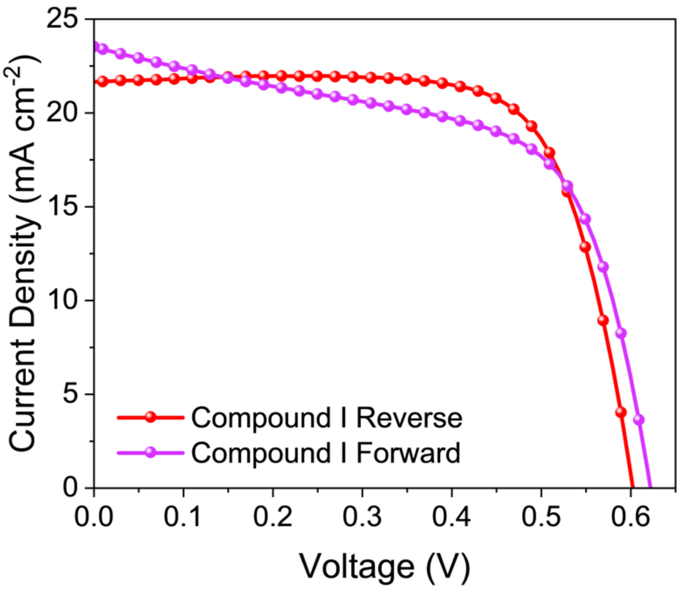

Figure 38: *J*-*V* data of forward and reverse scans of champion PSCs prepared using a Compound I interlayer.

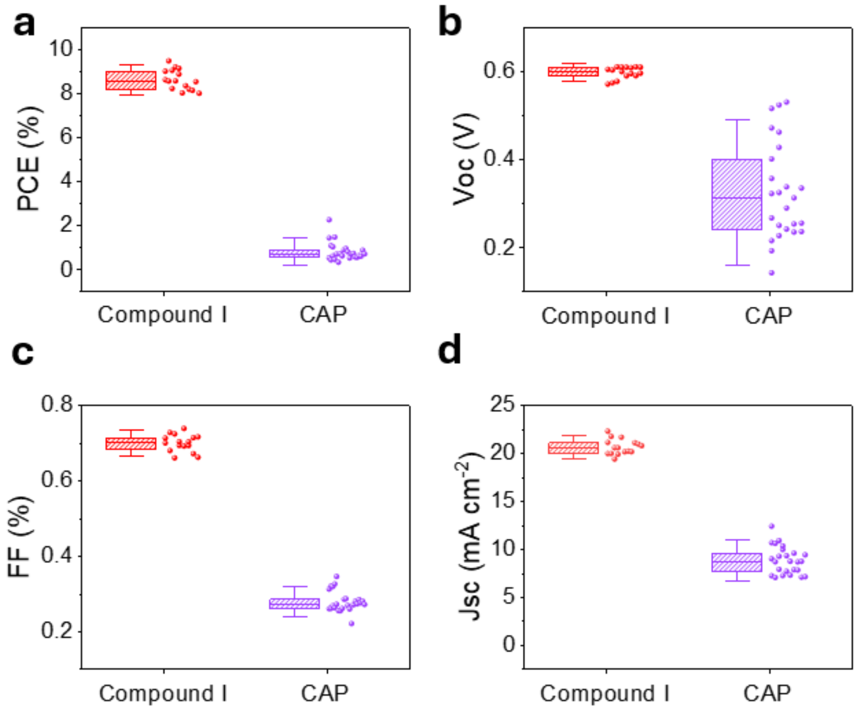

Figure 39: Device performance statistics for PSCs prepared with either Compound I or CAP molecule as an interlayer, **a** PCE **b**, *Voc* **c**, FF **d,** J_{sc} (derived from **16** devices with Compound I and 30 devices with CAP, where whiskers represent the minima and maxima; the bounds of the boxes represent the 25th and 75th percentiles; the centre is the statistical mean).

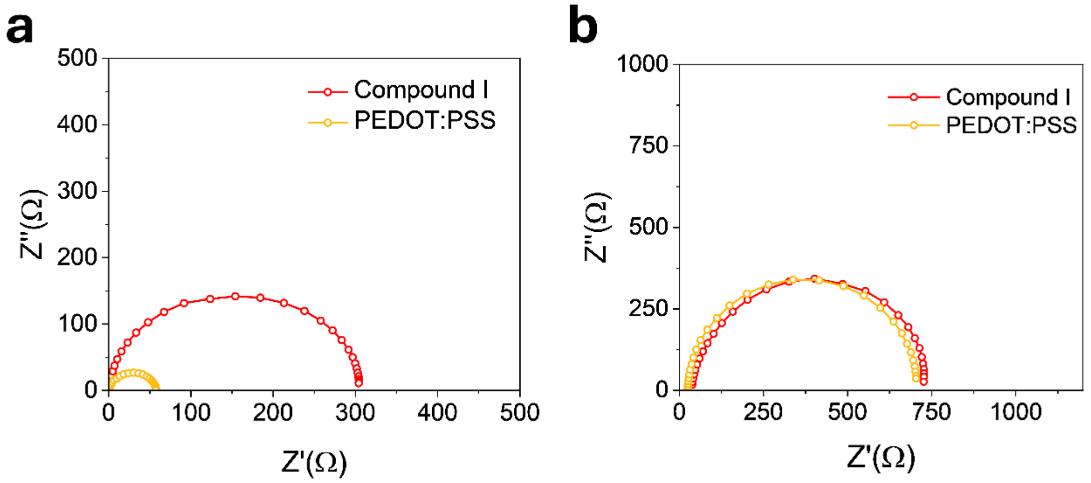

Figure 40: Electrochemical impedance spectroscopy (EIS) on Sn-PSCs under illumination prepared using Compound I (red) or PEDOT:PSS (yellow), a conventional polymeric HTL, under illumination and a DC bias of **a**, V_{OC}, **b**, 0.4 V corresponding to an operational voltage for hole transfer.

11 Quantum Chemical Calculations

11.1 Density Functional Theory

All density functional theory (DFT) and time-dependent (TD)DFT were performed with the ORCA electronic structure package 5.0[13,14]. Geometry optimization and vibrational frequency calculations were carried out at the BP86[15] and PBE0[16] level of theory, respectively using def2- variants of Ahlrichs' all-electron Gaussian triple-ζ valence polarized recontracted basis set (def2-TZVP)[17] on all atoms combined with D3BJ[18,19] dispersion correction and the AutoAux basis option for ORCA[20]. The calculations employed the resolution of identity (RI-J) algorithm for the computation of the Coulomb terms and the 'chain of spheres exchange' (COSX) algorithm for the calculation of the exchange terms[21] and a tight self-consistent field (SCF) convergence threshold was chosen via the "TightSCF" keyword. An increased grid was used during the SCF iterations (DefGrid2) and the conductor-like polarizable continuum model (CPCM) was used for charge compensation in all calculations of complexes carrying a net positive/negative charge[22]. Single-point energy DFT calculations used the B3LYP functional, ZORA-def2-TZVP basis set, AutoAux and the zeroth-order regular approximation (ZORA) to account for scalar relativistic effects[23,24]. Vibrational frequencies were calculated for all optimized structures and the absence of imaginary modes alongside six 0 cm^{-1} modes confirmed that minima were obtained in all cases.

11.2 Example Input Files

Geometry Optimisation

```
! UKS RIJK BP86 def2-TZVP AutoAux Freq TightSCF DefGrid2 SlowConv NormalPrint


%pal
nprocs 12
end

%maxcore 3000

%scf
MaxIter 700
End

*xyzfile charge spin-multiplicity xyz_filename.xyz
```

Single Point Energy

```
! UKS RIJCOSX B3LYP ZORA ZORA-def2-TZVP AutoAux TightSCF DefGrid2
SlowConv LargePrint
```

%pal
nprocs 12
end

%maxcore 3000

%scf
MaxIter 700
End

*xyzfile charge spin-multiplicity xyz_filename.xyz

Vibrational Frequencies

! UKS RIJCOSX B3LYP def2-TZVP AutoAux Freq TightSCF DefGrid2 SlowConv LargePrint

%pal
nprocs 12
end

%maxcore 3000

%scf
MaxIter 700
End

*xyzfile charge spin-multiplicity xyz_filename.xyz

11.3 XYZ coordinates for ORCA calculations

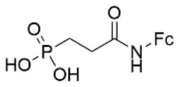

Charge = 0, Spin Multiplicity = 1

Fe	8.719871	8.138801	0.897250
N	7.322245	10.427561	-0.714248

C	7.220154	9.277792	0.085223
C	7.182548	9.329680	1.521823
C	7.009772	7.990778	2.003662
C	6.955167	7.114477	0.869391
C	7.091634	7.904204	-0.321802
C	10.343320	7.529939	-0.186600
C	10.478524	8.907750	0.192918
C	10.436254	8.978269	1.625727
C	10.277199	7.643571	2.132134
C	10.221071	6.748386	1.011797
H	7.261086	11.315430	-0.221145
H	7.273618	10.231860	2.121442
H	6.954873	7.694345	3.047516
H	6.851546	6.033105	0.900665
H	7.109451	7.545498	-1.343290
H	10.312070	7.148450	-1.203717
H	10.561361	9.752301	-0.486583
H	10.489472	9.885441	2.221905
H	10.189416	7.364388	3.178794
H	10.082122	5.671594	1.060243
O	7.745358	9.465200	-2.750482
C	7.602585	10.474875	-2.048842
C	7.758834	11.884085	-2.608896
H	7.329119	12.627555	-1.924692
H	8.841320	12.083848	-2.666347
C	7.135222	11.998621	-4.002673
H	6.040903	11.886682	-3.960288
H	7.518901	11.201112	-4.656692
O	9.068829	13.795030	-4.803462
P	7.469263	13.547022	-4.860638
O	6.944267	14.631177	-3.794773
H	7.034970	15.553466	-4.108312

O	6.897674	13.631846	-6.235170
H	9.520298	13.437779	-5.593653

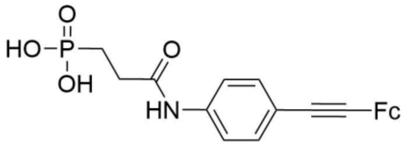

Charge = 0, Spin Multiplicity = 2

Fe	8.719871	8.138801	0.897250
N	7.322245	10.427561	-0.714248
C	7.220154	9.277792	0.085223
C	7.182548	9.329680	1.521823
C	7.009772	7.990778	2.003662
C	6.955167	7.114477	0.869391
C	7.091634	7.904204	-0.321802
C	10.343320	7.529939	-0.186600
C	10.478524	8.907750	0.192918
C	10.436254	8.978269	1.625727
C	10.277199	7.643571	2.132134
C	10.221071	6.748386	1.011797
H	7.261086	11.315430	-0.221145
H	7.273618	10.231860	2.121442
H	6.954873	7.694345	3.047516
H	6.851546	6.033105	0.900665
H	7.109451	7.545498	-1.343290
H	10.312070	7.148450	-1.203717
H	10.561361	9.752301	-0.486583
H	10.489472	9.885441	2.221905
H	10.189416	7.364388	3.178794
H	10.082122	5.671594	1.060243
O	7.745358	9.465200	-2.750482
C	7.602585	10.474875	-2.048842

C	7.758834	11.884085	-2.608896
H	7.329119	12.627555	-1.924692
H	8.841320	12.083848	-2.666347
C	7.135222	11.998621	-4.002673
H	6.040903	11.886682	-3.960288
H	7.518901	11.201112	-4.656692
O	9.068829	13.795030	-4.803462
P	7.469263	13.547022	-4.860638
O	6.944267	14.631177	-3.794773
H	7.034970	15.553466	-4.108312
O	6.897674	13.631846	-6.235170
H	9.520298	13.437779	-5.593653

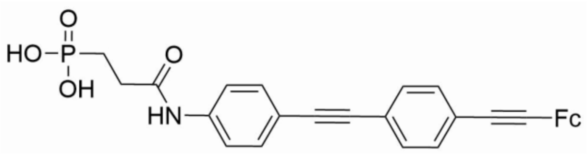

Charge = 0, Spin Multiplicity = 1

C	1.375460	12.059013	8.083344
C	-0.012061	11.938552	7.867656
H	-0.433048	12.235275	6.904752
C	-0.844218	11.445320	8.860989
H	-1.915080	11.355156	8.677298
C	-0.317389	11.053868	10.113887
C	1.073429	11.185831	10.321406
H	1.497449	10.891074	11.281946
C	1.912374	11.678341	9.327786
H	2.980276	11.768615	9.501705
C	-1.157127	10.537175	11.130537
C	-1.874268	10.081096	12.011149
C	-2.693750	9.544699	13.033011

C	-2.147108	9.212058	14.295612
H	-1.084599	9.378125	14.475926
C	-4.074673	9.322829	12.816398
H	-4.509184	9.574725	11.848465
C	-2.945586	8.679195	15.296566
C	-4.326321	8.453985	15.079180
C	-5.137691	7.903687	16.099581
C	-5.830390	7.411522	16.980345
C	-4.873209	8.788777	13.817085
H	-5.935346	8.621327	13.635978
H	-2.511008	8.426460	16.264194
Fe	-6.849078	4.861951	18.398499
C	-5.276049	3.548450	18.418913
C	-6.211859	6.546583	19.350912
C	-5.661771	3.846142	17.070241
C	-6.413030	2.984180	19.089729
C	-7.330600	5.985248	20.042891
C	-6.632041	6.856479	17.997731
C	-7.036139	3.470569	16.905843
C	-7.500812	2.935889	18.154749
C	-8.440032	5.924027	19.135187
C	-8.017542	6.446497	17.872767
H	-4.303169	3.742481	18.862427
H	-5.216595	6.716988	19.751141
H	-5.036449	4.322460	16.319317
H	-6.451949	2.674690	20.130730
H	-7.329769	5.637242	21.072320
H	-7.628822	3.593991	16.003456
H	-8.507197	2.582687	18.363107
H	-9.424249	5.520807	19.357711
H	-8.614446	6.529599	16.969033
N	2.146889	12.554941	7.021620

C	3.501528	12.766123	6.952931
H	1.621109	12.785055	6.180286
O	4.285245	12.523797	7.876806
C	3.975259	13.356018	5.629936
H	3.178291	13.330374	4.874983
H	4.211058	14.415348	5.820478
C	5.223699	12.626259	5.123864
H	4.989141	11.582696	4.860346
H	5.998014	12.597748	5.905374
P	5.993243	13.360247	3.661643
O	4.840537	13.550009	2.550712
O	7.211929	12.621275	3.225958
O	6.188220	14.933433	3.954908
H	6.971614	15.106845	4.512501
H	4.563539	12.699924	2.156559

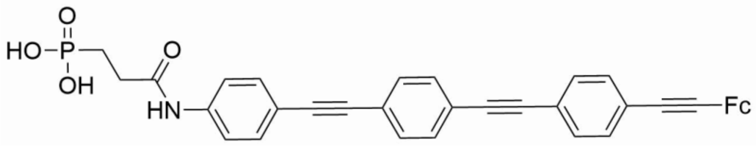

Charge = 0, Spin Multiplicity = 1

C	3.882773	13.466089	5.268963
C	2.497810	13.335197	5.041502
H	2.079184	13.654179	4.084687
C	1.665700	12.807318	6.016428
H	0.596639	12.711911	5.825176
C	2.189930	12.390010	7.262173
C	3.578529	12.527959	7.480335
H	4.000206	12.212106	8.435173
C	4.417507	13.055086	6.504884
H	5.483598	13.151538	6.686832
C	1.347767	11.848155	8.263080

C	0.625380	11.376362	9.130975
C	-4.195891	8.180705	15.084990
C	-3.646619	7.881302	16.354846
H	-2.586808	8.065522	16.533106
C	-5.572846	7.934355	14.868677
H	-6.007496	8.159644	13.894329
C	-4.440816	7.362058	17.365889
C	-5.819049	7.118215	17.151184
C	-6.628621	6.594450	18.186232
C	-7.322027	6.134182	19.083634
C	-6.366974	7.413850	15.879514
H	-7.426655	7.229512	15.700821
H	-4.006273	7.137349	18.340351
Fe	-8.371049	3.648055	20.593486
C	-6.818649	2.310267	20.638716
C	-7.698437	5.352828	21.483070
C	-7.221706	2.562877	19.285906
C	-7.953384	1.790536	21.348311
C	-8.820903	4.834545	22.201875
C	-8.124343	5.624167	20.123275
C	-8.604264	2.204147	19.157634
C	-9.056905	1.724896	20.433059
C	-9.938128	4.761220	21.304804
C	-9.517391	5.233280	20.022195
H	-5.835666	2.504571	21.059236
H	-6.697555	5.520837	21.870081
H	-6.601567	2.999509	18.507095
H	-7.980019	1.521328	22.400818
H	-8.817701	4.521953	23.242591
H	-9.209683	2.304266	18.260870
H	-10.065332	1.397003	20.670710
H	-10.926886	4.381843	21.548088

H	-10.120240	5.296099	19.120809
N	4.653194	14.007076	4.229924
C	6.002193	14.261167	4.184273
H	4.130451	14.256585	3.391996
O	6.785524	13.994941	5.100877
C	6.455767	14.947664	2.900578
H	5.725067	14.795402	2.094827
H	6.477608	16.029462	3.113501
C	7.845099	14.469595	2.473593
H	7.821549	13.416277	2.153533
H	8.548664	14.534206	3.317666
P	8.583210	15.410429	1.118150
O	7.475287	15.538955	-0.046953
O	9.911972	14.880022	0.701440
O	8.547484	16.965260	1.544997
H	9.282254	17.193499	2.147356
H	7.341019	14.695416	-0.522156
C	-3.384760	8.718060	14.058104
C	-2.677879	9.185295	13.174781
C	-1.854537	9.728621	12.160880
H	-0.041540	9.641435	13.346646
C	-0.469717	9.920385	12.383436
C	0.339091	10.457128	11.393372
H	1.404316	10.600436	11.576555
C	-0.202122	10.826287	10.138439
C	-1.586669	10.633605	9.915629
H	-2.015231	10.913383	8.952831
C	-2.395530	10.095552	10.905385
H	-3.460744	9.952207	10.722093

12 References

1. Bâldea, I. Exact Analytic Formula for Conductance Predicting a Tunable Sommerfeld–Arrhenius Thermal Transition within a Single-Step Tunneling Mechanism in Molecular Junctions Subject to Mechanical Stretching. *Adv Theory Simul* **5**, 2200158 (2022).
2. Jortner, J. Temperature dependent activation energy for electron transfer between biological molecules. *J Chem Phys* **64**, 4860–4867 (1976).
3. Bâldea, I. Can tunneling current in molecular junctions be so strongly temperature dependent to challenge a hopping mechanism? Analytical formulas answer this question and provide important insight into large area junctions. *Phys. Chem. Chem. Phys.* **26**, 6540–6556 (2024).
4. Nijhuis, C. A. *et al.* Control over Molecular Orbital Gating and Marcus Inverted Charge Transport in Molecular Junctions with Conjugated Molecular Wires. *Adv Electron Mater* **9**, 2200637 (2023).
5. Lloveras, V. *et al.* Tunneling versus Hopping in Mixed-Valence Oligo-p-phenylenevinylene Polychlorinated Bis(triphenylmethyl) Radical Anions. *J Am Chem Soc* **133**, 5818–5833 (2011).
6. Marcus, R. A. On the Theory of Oxidation-Reduction Reactions Involving Electron Transfer. I. *J Chem Phys* **24**, 966–978 (1956).
7. Marcus, R. A. & Sutin, N. Electron transfers in chemistry and biology. *Biochim. Biophys. Acta, Bioenerg.* **811**, 265–322 (1985).
8. Lee, H. J., Cho, S. J., Kang, H., He, X. & Yoon, H. J. Achieving Ultralow, Zero, and Inverted Tunneling Attenuation Coefficients in Molecular Wires with Extended Conjugation. *Small* **17**, 2005711 (2021).
9. Wang, G., Kim, Y., Choe, M., Kim, T.-W. & Lee, T. A New Approach for Molecular Electronic Junctions with a Multilayer Graphene Electrode. *Adv. Mater.* **23**, 755–760 (2011).
10. Sun, C.-Y. *et al.* Highly Stable Crystalline Catalysts Based on a Microporous Metal−Organic Framework and Polyoxometalates. *J Am Chem Soc* **131**, 1883–1888 (2009).
11. Sedghi, G. *et al.* Single Molecule Conductance of Porphyrin Wires with Ultralow Attenuation. *J Am Chem Soc* **130**, 8582–8583 (2008).
12. Bâldea, I. Can tunneling current in molecular junctions be so strongly temperature dependent to challenge a hopping mechanism? Analytical formulas answer this question and provide important insight into large area junctions. *Phys. Chem. Chem. Phys.* **26**, 6540–6556 (2024).
13. Neese, F. Software update: The ORCA program system—Version 5.0. *WIREs Comput. Mol. Sci.* **12**, e1606 (2022).

14. Neese, F. The ORCA program system. *WIREs Comput. Mol. Sci.* **2**, 73–78 (2012).

15. Lee, C., Yang, W. & Parr, R. G. Development of the Colle-Salvetti correlation-energy formula into a functional of the electron density. *Phys Rev B* **37**, 785–789 (1988).

16. Perdew, J. P., Ernzerhof, M. & Burke, K. Rationale for mixing exact exchange with density functional approximations. *J Chem Phys* **105**, 9982–9985 (1996).

17. Weigend, F. & Ahlrichs, R. Balanced basis sets of split valence, triple zeta valence and quadruple zeta valence quality for H to Rn: Design and assessment of accuracy. *Phys. Chem. Chem. Phys.* **7**, 3297–3305 (2005).

18. Grimme, S., Ehrlich, S. & Goerigk, L. Effect of the damping function in dispersion corrected density functional theory. *J Comput Chem* **32**, 1456–1465 (2011).

19. Grimme, S., Antony, J., Ehrlich, S. & Krieg, H. A consistent and accurate ab initio parametrization of density functional dispersion correction (DFT-D) for the 94 elements H-Pu. *J Chem Phys* **132**, 154104 (2010).

20. Stoychev, G. L., Auer, A. A. & Neese, F. Automatic Generation of Auxiliary Basis Sets. *J Chem Theory Comput* **13**, 554–562 (2017).

21. Neese, F., Wennmohs, F., Hansen, A. & Becker, U. Efficient, approximate and parallel Hartree–Fock and hybrid DFT calculations. A 'chain-of-spheres' algorithm for the Hartree–Fock exchange. *Chem Phys* **356**, 98–109 (2009).

22. Klamt, A. & Schüürmann, G. COSMO: a new approach to dielectric screening in solvents with explicit expressions for the screening energy and its gradient. *J. Chem. Soc., Perkin Trans. 2* 799–805 (1993).

23. Pantazis, D. A., Chen, X.-Y., Landis, C. R. & Neese, F. All-Electron Scalar Relativistic Basis Sets for Third-Row Transition Metal Atoms. *J Chem Theory Comput* **4**, 908–919 (2008).

24. van Lenthe, E., Wormer, P. E. S. & van der Avoird, A. Density functional calculations of molecular g-tensors in the zero-order regular approximation for relativistic effects. *J Chem Phys* **107**, 2488–2498 (1997).
