# Supplementary material for: Electron hopping in conjugated molecular wires with application to solar cells
Source: Nat Chem. 2026 Feb 9;18(4):756–64. doi: 10.1038/s41557-025-02034-0 (PMC13061622; doi:10.1038/s41557-025-02034-0)
Supplement: Supplementary file 2 — Reporting Summary [file 41557_2025_2034_MOESM2_ESM.pdf]

nature research

Corresponding author(s):

☐ Initial submission ☐ Revised version ☑ Final submission

Solar Cells Reporting Summary

Nature Portfolio wishes to improve the reproducibility of the work that we publish. This form is intended for publication with all accepted papers reporting the characterization of photovoltaic devices and provides structure for consistency and transparency in reporting. Some list items might not apply to an individual manuscript, but all fields must be completed for clarity.

For further information on Nature Research policies, including our data availability policy, see Authors & Referees.

Please check the following details are reported in the manuscript, and provide a brief description or explanation where applicable.

Area of the tested solar cells — ☑ Yes ☐ No

Report the area of the tested solar cells.

0.045cm^2

Method used to determine the device area — ☑ Yes ☐ No

Provide a description of the method and state where this information can be found in the text.

Methods section.

2. Current-voltage characterization

Current density-voltage (J-V) plots in both forward and backward direction — ☑ Yes ☐ No

Yes

Voltage scan conditions — ☑ Yes ☐ No

Provide a description of the measurement conditions (e.g. scan direction, speed, dwell times).

a scan rate of 50 mV/s in forward and reverse bias

Test environment — ☑ Yes ☐ No

Provide a description of the test conditions (e.g. characterization temperature, atmosphere, humidity).

ambient conditions / N2

Protocol for preconditioning of the device before its characterization — ☐ Yes ☑ No

Provide a description of the protocol.

Stability of the J-V characteristic — ☑ Yes ☐ No

Provide a description of the method used. The stability of the J-V characteristic can be verified with time evolution of the maximum power point or with the photocurrent at maximum power point; see ref. 5 for details.

stabilized power output measured for 300 seconds

Description of the unusual behaviour observed during the characterization — ☐ Yes ☑ No

Provide a description of hysteresis or any other unusual behaviour observed during the characterization.

not relevant to our study

Related experimental data — ☐ Yes ☑ No

Provide a description of the related experimental data.

not relevant to our study

External quantum efficiency (EQE) or incident photons to current efficiency (IPCE) — ☑ Yes ☐ No

Provide a description of the technique used.

in methods

A comparison between the integrated response under the standard reference spectrum and the response measure under the simulator — ☑ Yes ☐ No

in methods

For tandem solar cells, the bias illumination and bias voltage used for each subcell

☐ Yes ☑ No

Provide a description of the measurement conditions.

Not relevant to our study.

5. Calibration

Light source and reference cell or sensor used for the characterization

☑ Yes ☐ No

Provide a description of the light source and reference cell or sensor.

Current-voltage (J-V) characteristics were performed under 1 sun (AM 1.5G) illumination using a Keithley 2400 source meter with a xenon lamp (Newport).

Confirmation that the reference cell was calibrated and certified

☐ Yes ☑ No

Identify the independent certification laboratory.

Not relevant to our study.

Calculation of spectral mismatch between the reference cell and the devices under test

☐ Yes ☑ No

Provide a value of the spectral mismatch and/or a description of how it has been taken into account in the measurements.

Not relevant to our study.

6. Mask/aperture

Size of the mask/aperture used during testing

☐ Yes ☑ No

Report the size of the mask/aperature.

Not relevant to our study.

Variation of the measured short-circuit current density with the mask/aperture area

☐ Yes ☑ No

Report the difference in the short-circuit current density values measured with the mask and aperture area.

Not relevant to our study.

7. Performance certification

Identity of the independent certification laboratory that confirmed the photovoltaic performance

☐ Yes ☑ No

Identify the independent certification laboratory.

Not relevant to our study.

A copy of any certificate(s)

☐ Yes ☑ No

Certificate copies should be provided in the Supplementary information. Please state the supplementary item number.

Not relevant to our study.

8. Statistics

Number of solar cells tested

☑ Yes ☐ No

Report how many solar cells have been tested, specifying the number of individual substrates.

see methods

Statistical analysis of the device performance

☑ Yes ☐ No

see methods

9. Long-term stability analysis

Type of analysis, bias conditions and environmental conditions

☐ Yes ☑ No

Provide a description of the type of analysis, bias conditions and environmental conditions (e.g. illumination type, temperature, atmosphere humidity, encapsulation method, preconditioning temperature, bias) for each long-term stability analysis carried out; see ref. 7 and 8 for details.

1. Shrotriya, V. *et al*. Accurate measurement and characterization of organic solar cells. *Adv. Funct. Mater.* **16**, 2016–2023 (2006).
2. Dennler, G. *et al*. The value of values. *Mat. Today* **10**, 56 (2007).
3. Cravino, A., Schilinsky, P. & Brabec, C. J. Characterization of organic solar cells: the importance of device layout. *Adv. Funct. Mater.* **17**, 3906–3910 (2007).
4. Reese, M. O. *et al*. Consensus stability testing protocols for organic photovoltaic materials and devices. *Sol. Energ. Mat. Sol. C* **95**, 1253–1267 (2011).
5. Snaith H. .J. The perils of solar cell efficiency measurements. *Nat. Photon*. **6**, 337–340 (2012).
6. Luber, E. J. & Buriak, J. M. Reporting performance in organic photovoltaic devices. *ACS Nano* **7**, 4708–4714 (2013).
7. Snaith, H. J. *et al*. Anomalous hysteresis in perovskite solar cells. *J. Phys. Chem. Lett.* **5**, 1511–1515 (2014).
8. Grätzel M. The light and shade of perovskite solar cells. *Nat. Mat*. **13**, 838–842 (2014).
9. Zimmermann E. *et al*. Erroneous efficiency reports harm organic solar cell research. *Nat. Photon*. **8**, 669–672 (2014).
10. Beard M.C., Luther J.M. & Nozik A.J. The promise and challenge of nanostructured solar cells. *Nat. Nanotech*. **9**, 951–954 (2014).
11. Timmreck, R. *et al*. Characterization of tandem organic solar cells. *Nat. Photon.* **9**, 478–479 (2015).

A number of international committees develop industry standards on the characterization of photovoltaic technologies (for example ASTM-E44 and IEC-TC 82), which can provide guidance for academic research.

This checklist template is licensed under a Creative Commons Attribution 4.0 International License, which permits use, sharing, adaptation, distribution and reproduction in any medium or format, as long as you give appropriate credit to the original author(s) and the source, provide a link to the Creative Commons license, and indicate if changes were made. The images or other third party material in this article are included in the article's Creative Commons license, unless indicated otherwise in a credit line to the material. If material is not included in the article's Creative Commons license and your intended use is not permitted by statutory regulation or exceeds the permitted use, you will need to obtain permission directly from the copyright holder. To view a copy of this license, visit http://creativecommons.org/licenses/by/4.0/
