# Supplementary figures and images for: Electron hopping in conjugated molecular wires with application to solar cells
Source: Nat Chem. 2026 Feb 9;18(4):756–64. doi: 10.1038/s41557-025-02034-0 (PMC13061622; doi:10.1038/s41557-025-02034-0)

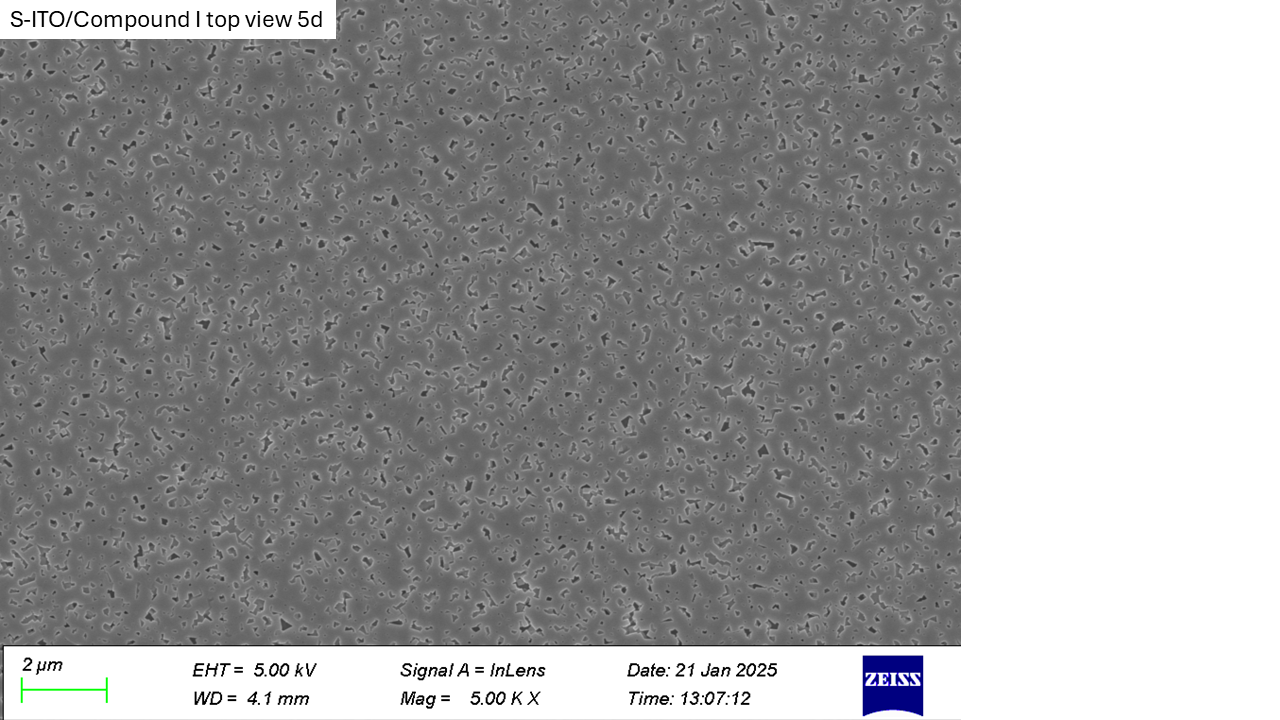

Supplement: Supplementary file 7 — The excel file for the energy levels of compounds I–III and aminoferrocene. [file 41557_2025_2034_MOESM7_ESM.zip › S-ITO_Compound_I_topview_5d.TIF]

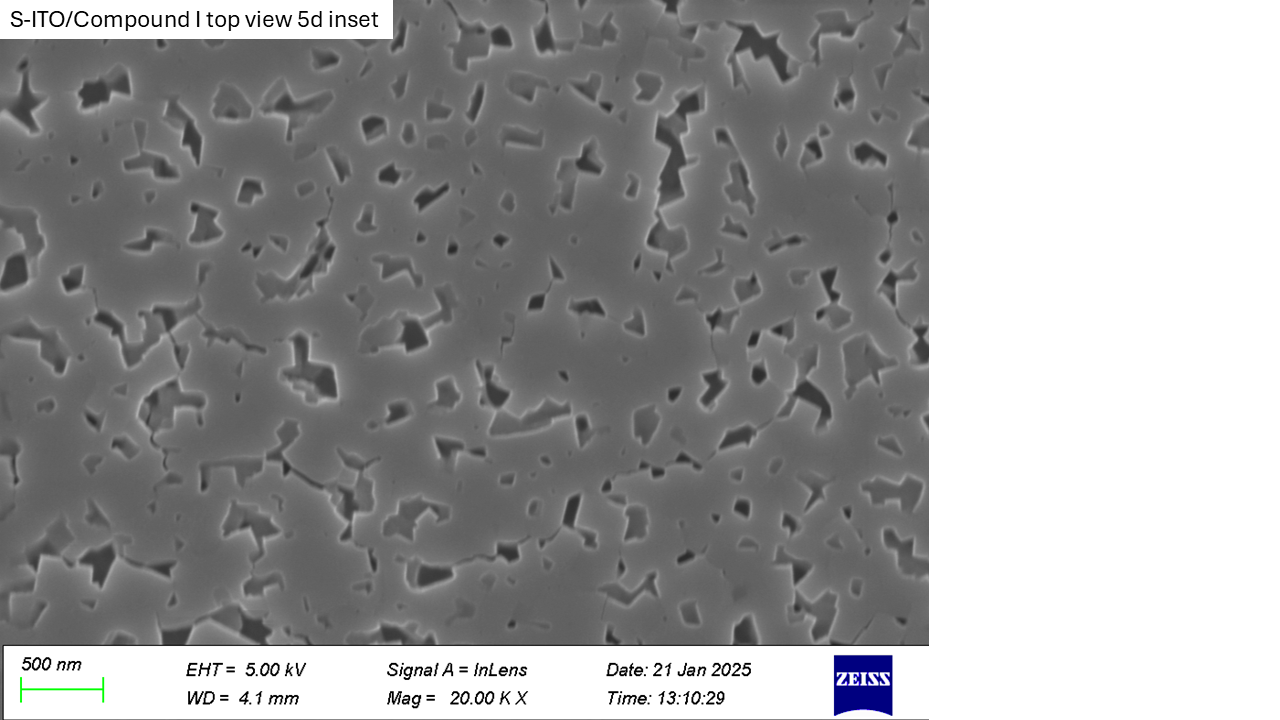

Supplement: Supplementary file 7 — The excel file for the energy levels of compounds I–III and aminoferrocene. [file 41557_2025_2034_MOESM7_ESM.zip › S-ITO_Compound_I_top_view_inset_5d.TIF]

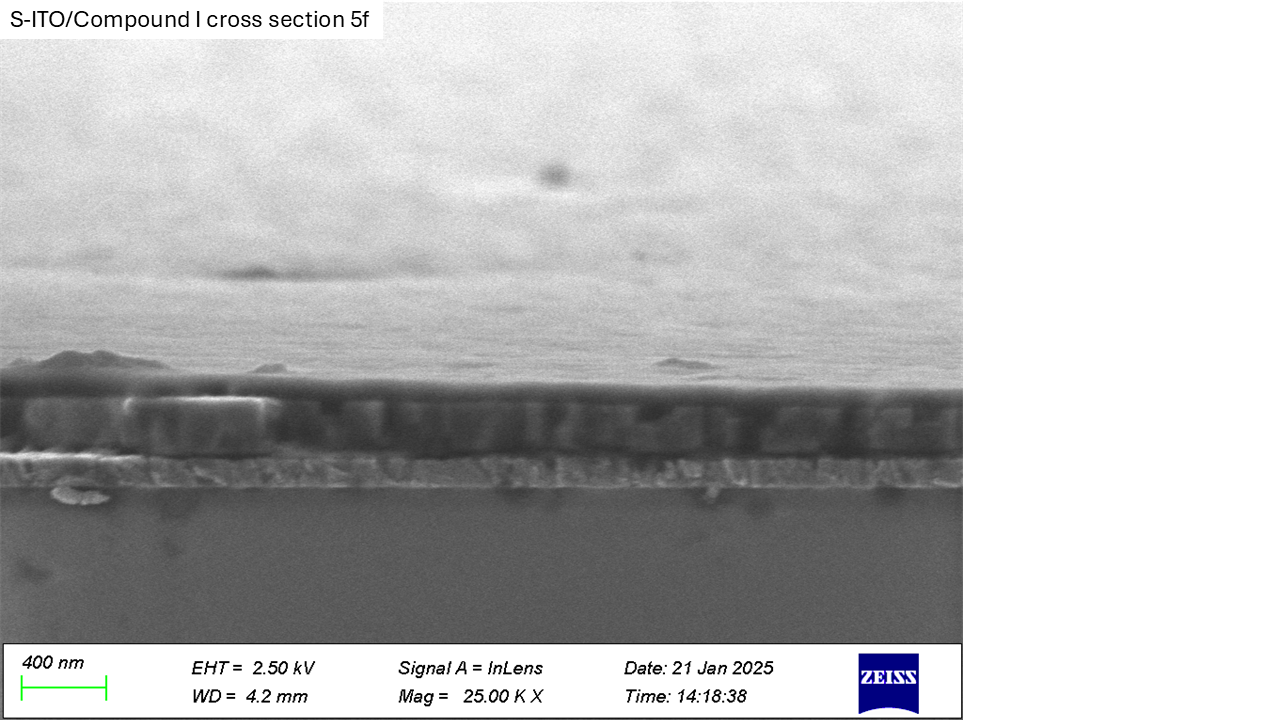

Supplement: Supplementary file 7 — The excel file for the energy levels of compounds I–III and aminoferrocene. [file 41557_2025_2034_MOESM7_ESM.zip › S-ITO_Compound_I_Cross_section_5f.TIF]

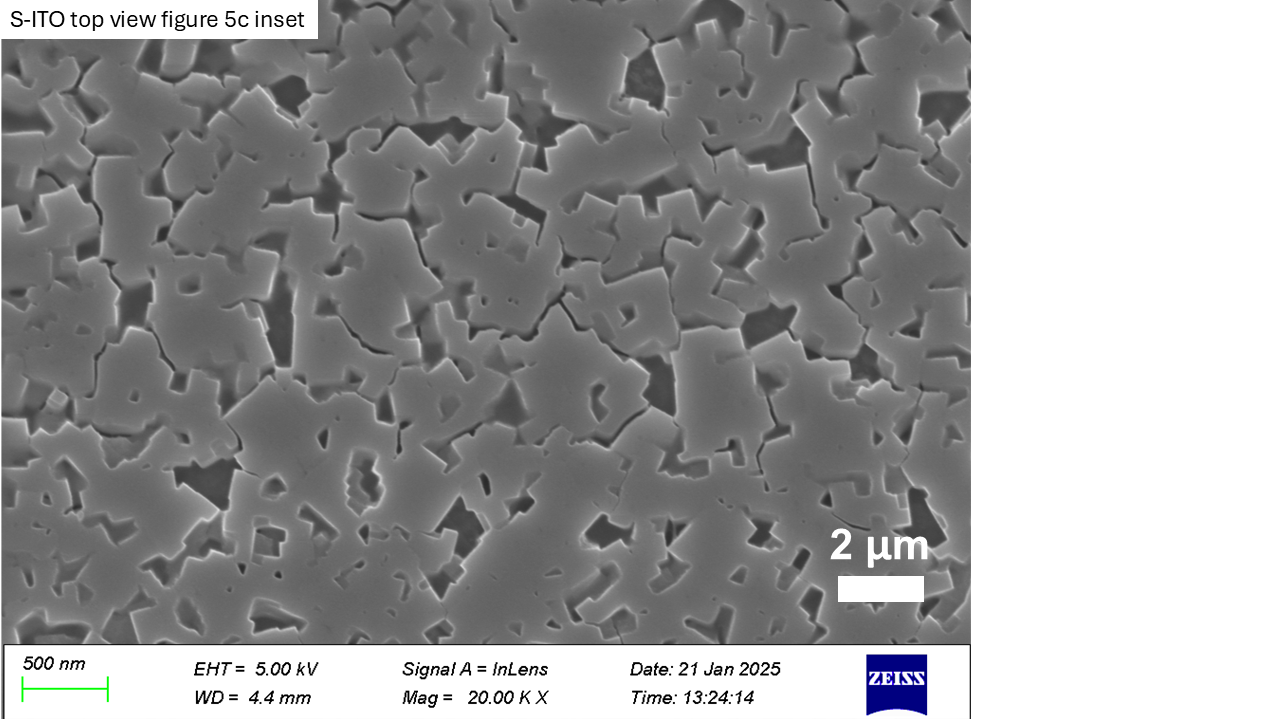

Supplement: Supplementary file 7 — The excel file for the energy levels of compounds I–III and aminoferrocene. [file 41557_2025_2034_MOESM7_ESM.zip › S-ITO_topview_inset_5c.TIF]

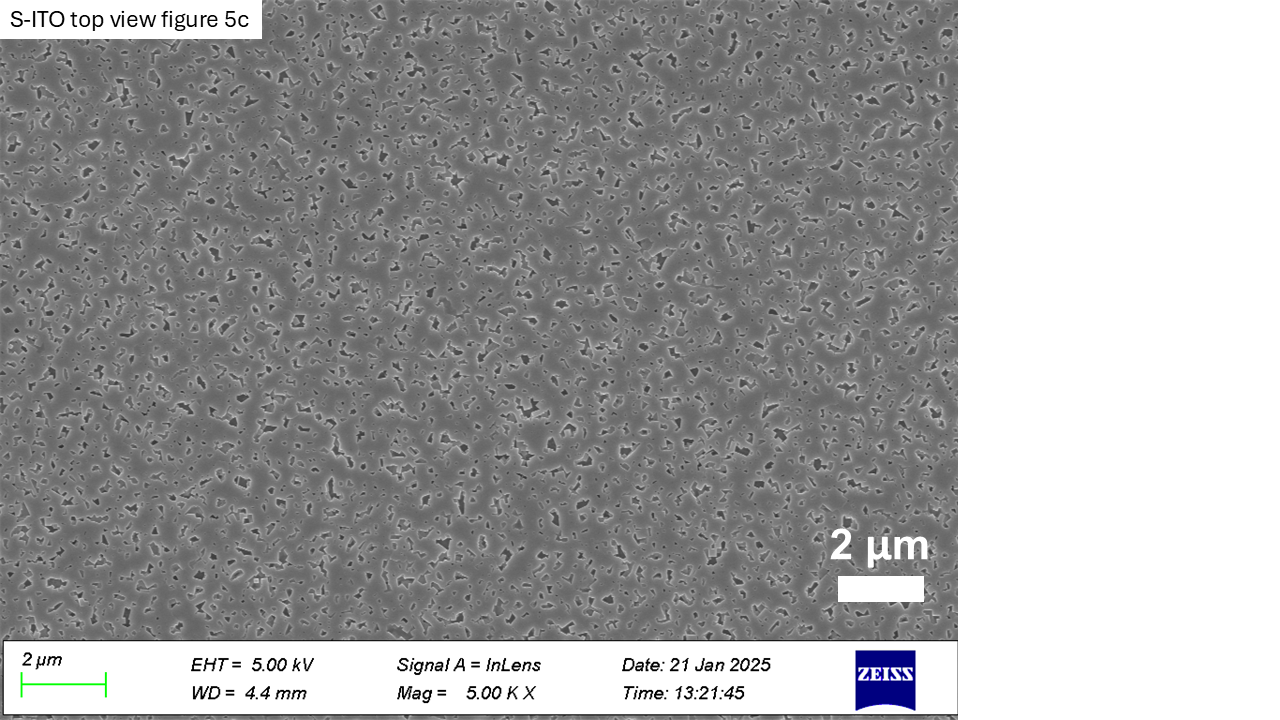

Supplement: Supplementary file 7 — The excel file for the energy levels of compounds I–III and aminoferrocene. [file 41557_2025_2034_MOESM7_ESM.zip › S-ITO_topview_5c.TIF]

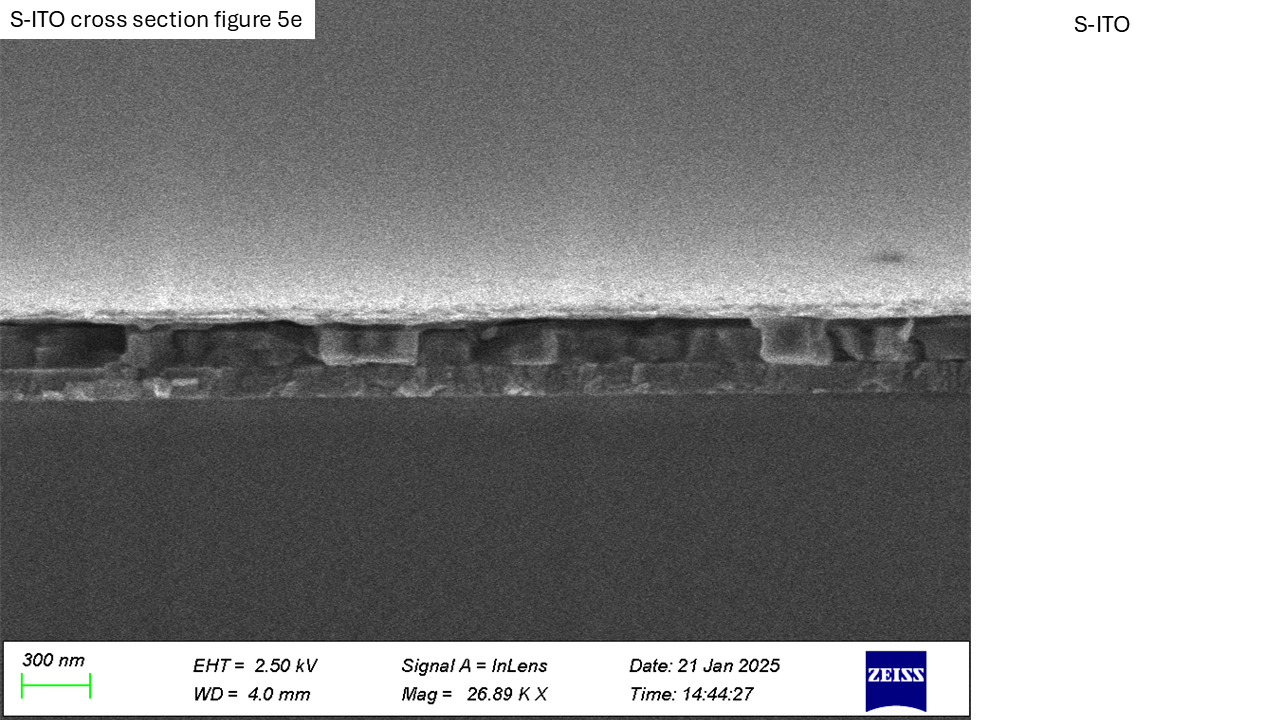

Supplement: Supplementary file 7 — The excel file for the energy levels of compounds I–III and aminoferrocene. [file 41557_2025_2034_MOESM7_ESM.zip › S-ITO_cross_section_5e.TIF]

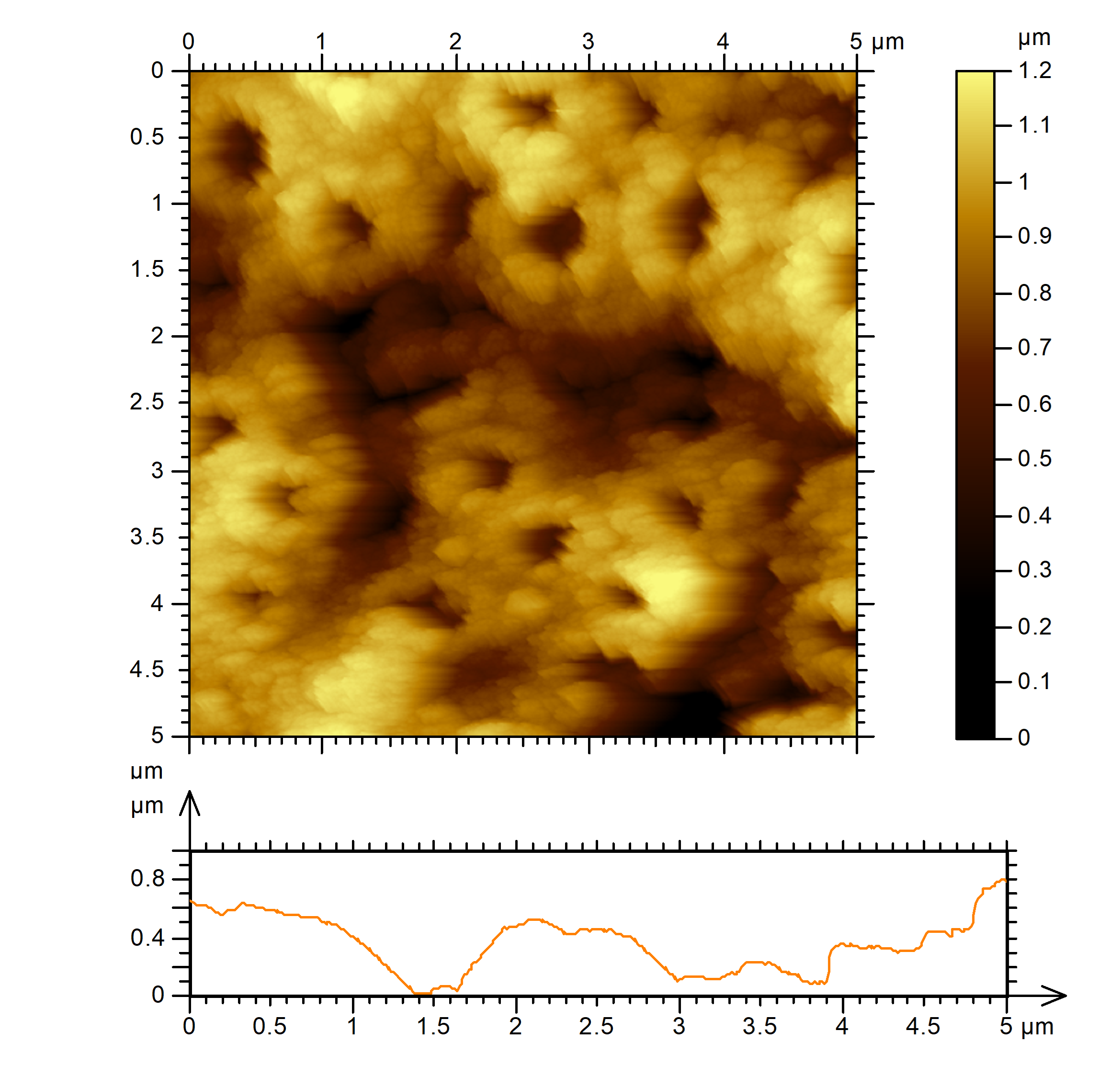

Supplement: Supplementary file 13 — Separate TIF files are given for each unprocessed SEM image of the top view (including inset) and cross section of the S-ITO and S-ITO/compound I images. 5g_XRD is the raw XRD data for the diffraction patterns of perovskite grown on S-ITO and S-ITO/compound I. [file 41557_2025_2034_MOESM13_ESM.zip › IO-mesoITO_AFM_source_data.tif]
